# Genome-Wide Identification, Characterization and Expression Analysis of the *TCP* Gene Family in *Prunus mume*

**DOI:** 10.3389/fpls.2016.01301

**Published:** 2016-08-31

**Authors:** Yuzhen Zhou, Zongda Xu, Kai Zhao, Weiru Yang, Tangren Cheng, Jia Wang, Qixiang Zhang

**Affiliations:** ^1^Beijing Key Laboratory of Ornamental Plants Germplasm Innovation and Molecular Breeding, National Engineering Research Center for Floriculture, Beijing Laboratory of Urban and Rural Ecological Environment, Key Laboratory of Genetics and Breeding in Forest Trees and Ornamental Plants of Ministry of Education, School of Landscape Architecture, Beijing Forestry UniversityBeijing, China; ^2^College of Forestry, Shandong Agricultural UniversityTai’an, China

**Keywords:** Genome-wide, *TCP* genes, expression analysis, *Prunus mume*, flower development

## Abstract

TCP proteins, belonging to a plant-specific transcription factors family, are known to have great functions in plant development, especially flower and leaf development. However, there is little information about this gene family in *Prunus mume*, which is widely cultivated in China as an ornamental and fruit tree. Here a genome-wide analysis of *TCP* genes was performed to explore their evolution in *P. mume*. Nineteen *PmTCPs* were identified and three of them contained putative miR319 target sites. Phylogenetic and comprehensive bioinformatics analyses of these genes revealed that different types of *TCP* genes had undergone different evolutionary processes and the genes in the same clade had similar chromosomal location, gene structure, and conserved domains. Expression analysis of these *PmTCPs* indicated that there were diverse expression patterns among different clades. Most *TCP* genes were predominantly expressed in flower, leaf, and stem, and showed high expression levels in the different stages of flower bud differentiation, especially in petal formation stage and gametophyte development. Genes in TCP-P subfamily had main roles in both flower development and gametophyte development. The CIN genes in double petal cultivars might have key roles in the formation of petal, while they were correlated with gametophyte development in the single petal cultivar. The CYC/TB1 type genes were highly detected in the formation of petal and pistil. The less-complex flower types of *P. mume* might result from the fact that there were only two CYC type genes present in *P. mume* and a lack of CYC2 genes to control the identity of flower types. These results lay the foundation for further study on the functions of *TCP* genes during flower development.

## Introduction

*Prunus mume* Sieb. et Zucc. (Rosaceae, Prunoideae) has been cultivated in China for over 3,000 years for its prominent ornamental and economic value. This species has acquired favorable ornamental characteristics with various types of flowers, colorful corollas, pleasing fragrance, and early blooming in the *Prunus* genus (Zhang et al., 2012; Xu Z. et al., 2014). The flower development plays a vital role in ornamental value. In *P. mume*, the studies of the MADS-box gene family and SBP-box gene family have been performed to gain insights into flower development (Xu Z. et al., 2014; Xu et al., 2015). Nevertheless, the molecular regulation of its flower development are still almost unknown. The *TCP* genes have been involved in different aspects of plant growth, including: flower development, flower symmetry, shoot branching, leaf development, leaf morphogenesis, leaf senescence, male and female gametophyte development, and circadian clock (Palatnik et al., 2003; Li et al., 2005; Aguilar-Martinez et al., 2007; Busch and Zachgo, 2007; Koyama et al., 2007, 2010; Poza-Carrion et al., 2007; Efroni et al., 2008; Schommer et al., 2008; Nag et al., 2009; Giraud et al., 2010; Sarvepalli and Nath, 2011; Yang et al., 2012). The genome-wide analysis of *TCP* gene in *P. mume* is important to learn more about the molecular mechanisms of flower development.

The *TCP* genes encode conserved sequences of about 60 amino acids designated as the TCP domain. This domain may be important for activation or repression of transcription and is involved in protein–protein interactions (Manassero et al., 2013). A basic helix-loop-helix (bHLH) structure formed from the TCP domain is quite different from the HLH structure which is a DNA-binding domain of the transcription factors both in plants and animals (Cubas et al., 1999). The name of TCP was derived from the four first finding members of the *TCP* gene family, *Teosinte Branched 1* (*TB1*) from *Zea mays*, *CYCLOIDEA* (*CYC*) from *Antirrhinum majus*, and the two *Oryza sativa* genes, *PROLIFERATING CELL FACTORS 1* and *2* (*PCF1* and *PCF2*). So far, many *TCP* genes have been identified in various plant species. For example, there are 24 *TCP* genes in *Arabidopsis*, 28 *TCP* genes in *O. sativa*, 30 *TCP* genes in *Solanum lycopersicum*, 52 *TCP* genes in *Malus domestica*, and so on (Martin-Trillo and Cubas, 2010; Parapunova et al., 2014; Xu R. et al., 2014). Based on the differences of the TCP domains, the TCP proteins are divided into two subfamilies (Classes I and II). In the Class I, biochemical characterization of TCP proteins has been studied mainly in rice (*O. sativa*). PCF1 and PCF2 are identified as DNA-binding proteins that have the ability to bind specifically to *PROLIFERATING CELL NUCLEAR ANTIGEN* (*PCNA*) promoter (Kosugi and Ohashi, 1997). These genes have been suggested to promote plant proliferation and growth, based on their expression in meristematic tissues. Most single mutants in class I have mild or no phenotypic defects (Takeda et al., 2006; Herve et al., 2009). In contrast, the class II genes are proposed to have a role in preventing growth and proliferation by observing the single and multiple mutants in their phenotypes (Hubbard et al., 2002; Aguilar-Martinez et al., 2007; Kim et al., 2008; Martin-Trillo and Cubas, 2010). The Class II is further subdivided into two clades: CIN clade and CYC/TB1 clade. The CIN clade genes exemplified by *CINCINNATA* (*CIN*) of *A. majus* participate in lateral organ development, and the CYC/TB1 clade genes basically have functions in flower development or lateral shoot development (Martin-Trillo and Cubas, 2010). An R domain is acquired in most members of the CYC/TB1 clade, but little members of the CIN clade contain the R domain (Cubas et al., 1999). Because a glutamic acid-cysteine-glutamic acid stretch has been found between the TCP and R domains in the subset of CYC/TB1 genes, CYC/TB1 clade is also called ECE clade (Howarth and Donoghue, 2006). Phylogenetic analyses reveal that the CYC/TB1 clade can be classified into three types of genes which have expanded by duplication in the groups: CYC1, CYC2, and CYC3. In *Arabidopsis*, there are no CYC2 genes which have a primary role in the development of floral dorsoventral zygomorphy (asymmetry). Genetic studies in *Arabidopsis* also suggest that CYC1 genes have retained the TB1 function to control the shoot branching. The CYC3 includes *Arabidopsis* BRANCHED1, expressed both in flower primordia and branches, indicating the unclear functions in the flower development (Aguilar-Martinez et al., 2007; Poza-Carrion et al., 2007).

MicroRNAs are a kind of non-coding small RNAs that regulate gene expression by binding target mRNAs, which causes translational inhibition or cleavage. In the CIN clade, there are some microRNA miR319 target genes that have been analyzed so far. MiR159 and miR319 have a common ancestor and about 17 identical nucleotides. These miRNAs interact with each other and have overlapping roles in regulating floral development (Hong and Jackson, 2015). To identify networks controlled by TCPs or miR319, microarray analysis of transgenic plants with different levels of these transcription factors have been executed (Palatnik et al., 2003; Schommer et al., 2008, 2012, 2014). The expression levels of miR319-targeted *TCP* genes were decreased by miR319 activity, while other *TCP* genes, which lack miR319 target sites, were unaffected. An excess of cell expansion that generated a super compound organ in tomato (*S. lycopersicum*) or a crinkled simple leaf in *Arabidopsis* and *A. majus* was caused by low TCP activity or high levels of miR319 (Nath et al., 2003; Palatnik et al., 2003; Ori et al., 2007). Current evidences indicated that these TCP transcription factors were regulated by miR319 which guides them to cleavage. This pathway supported the importance of the control of plant growth and development.

In this study, we first identified 19 *TCP* genes in *P. mume*, and then carried on comprehensive bioinformatics analyses of phylogeny, gene structure, conserved domains, chromosomal location and miRNA target sites. Finally, transcriptome sequencing (RNA-seq) was employed to study the expression patterns of these genes in various organs. In order to clarify their functions in floral organ development, the real-time quantitative RT-PCR were also performed at different developmental stages of flower bud differentiation. This study presents the first genome-wide analysis of the *TCP* gene family in *Prunus* species. The results of this study provide the foundation for further functional analyses of *TCP* genes in *P. mume* and other *Prunus* species. These results will also broaden our insight into the roles of *TCP* genes in regulating flower development and flower organ determination.

## Materials and Methods

### Identification and Annotation of *P. mume TCP* Genes

*Prunus mume* genome sequences were acquired from the *P. mume* genome database^1^. To identify *TCP* genes in *P. mume*, TCP domain HMM profile^2^ (PF03634) was used as a query to perform HMMER searches against the *P. mume* genome database. As recommended by the HMMER user’s guide, an *e*-value threshold of 0.1 was carried out in these searches (Finn et al., 2011). All obtained sequences were put in InterPro^3^ to assure the existence of the TCP domain. Based on the results from InterPro, the sequences which did not include the TCP domain were eliminated. The names of *P. mume TCP* genes were assigned according to their scores for complete sequences out of HMMER searches. *TCP* genes in *Arabidopsis thaliana* were downloaded from TAIR^4^ using the identifiers reported by Martin-Trillo and Cubas (2010).

### Phylogenetic and Gene Structure Analysis

To study the phylogenetic relationships between *TCP* genes in *P. mume* and other species, the protein sequences of the identified *P. mume TCP* genes, 24 *A. thaliana TCP* genes, a Teosinte Branched1 (TB1) from *Z. mays*, a CYCLOIDEA (CYC) from *A. majus*, and the PROLIFERATING CELL FACTORS 1 and 2 (PCF1 and PCF2) from *O. sativa* (Supplementary Data 3), were used to generate the phylogenetic tree. First, multiple sequence alignments were performed using Clustal X2.0 (Supplementary Figure 3) (Larkin et al., 2007). Subsequently, phylogenetic trees were constructed by MEGA7.1 with the Maximum-likelihood (ML) method. These analyses were performed with default parameters except the bootstrap analysis which was employed using 1,000 replicates. The genomic sequences and structural information of *P. mume TCP* genes were downloaded from the *P. mume* genome database (Supplementary Data 1 and 2). Diagrams of exon-intron structures were obtained using Gene Structure Display Server 2.0^5^.

### Conserved Domains and Motif Analysis

All protein sequences of *PmTCPs* were submitted to the MEME online tool^6^ to identify conserved motif and structural divergences. These sequences were analyzed in MEME with the following parameters: repetition number, any; maximum motif number, 20; maximum motif width, 60; minimum motif width, 6. To annotate the identified motifs, Pfam and SMART^7^ were used. Multiple sequence alignment was carried out with DNAMAN. Protein sequence logo was created by the Weblogo online tool^8^.

### Chromosomal Location and miR159/miR319 Target Site Prediction

On the basis of positional information in the *P. mume* genome project^9^, locations of the *TCP* genes on the *P. mume* chromosomes were assessed using MapDraw and Photoshop software. To predict miR159 and miR319 target sites, full-length *PmTCPs* nucleotide sequences were analyzed using the psRNATarget online application^10^.

### Expression Analysis

Transcriptome sequencing (RNA-seq) was performed to study expression profiles of *PmTCPs*. Total RNA was extracted from five organs of *P. mume*: roots, stems, leaves, flowers, and fruits. The sequencing and assembly was executed at the Beijing Genomics Institute. Genesis software was used to standardize the expression data and estimate hierarchical clustering (Sturn et al., 2002).

### Real-Time Quantitative RT-PCR

The expressions of 19 *PmTCPs* in flower buds at different development stages were examined using real-time RT-PCR. The materials of the gene expression pattern analysis in different periods of flower bud development were taken from the bud of ‘Jiang Mei,’ ‘Sanlun Yudie,’ and ‘Subai Taige.’ Two samples of basic consistent appearance were taken in every 5–7 days. One was sampled to define the development stages of the flower bud using paraffin section method; the other one was quickly frozen in liquid nitrogen and used for the extraction of RNA. Total RNA was extracted from flower buds at eight development stages (S1, S2, S3, S4, S5, S6, S7, and S8) using Trizol reagent (Invitrogen, USA) according to the manufacturer’s instructions. And potentially residual genomic DNA was removed from RNA samples using RNase-free DNase (Promega, USA). To synthesis fist-strand cDNA from 2 μg total RNA, the TIANScript First Strand cDNA Synthesis Kit (Tiangen, China) was used following the manufacturer’s instructions. Real-time RT-PCR was completed with PikoReal real-time PCR system (Thermo Fisher Scientific, Germany) and reactions were performed in a 10 μl volume including 0.5 μl of cDNA, 200 nM of each primer (Supplementary Table 1) and 5 μl of SYBR Premix Ex*Taq* II (Takara, China). The reactions were completed in the following conditions: 30 s at 95°C, 40 cycles of 5 s at 95°C and 30 s at 60°C, 30 s at 60°C, end in 20°C and the temperature of melting curve was set from 60 to 95°C rising at 0.2°C/s. The amplification efficiency of each primer pair was investigated by melting curve analysis. All experiments were performed with three biological duplications, and each duplication was repeated in triplicate. With the protein phosphatase 2A (*PP2A1*) gene of *P. mume* as the reference gene (Wang et al., 2014a), the relative expression levels were calculated with the 2^-ΔΔCt^ method.

## Results

### Identification and Chromosomal Location of *TCP* Genes in *P. mume*

To identify the *TCP* genes in *P. mume*, HMMER searches were employed to search against the protein database of *P. mume* using TCP domain HMM profile PF03634 as the search query. Through the search, a total of 19 sequences were obtained. The Interpro analysis revealed that each of the 19 protein sequences contained the TCP domain, indicating that there were at least 19 *TCP* genes in the *P. mume* genome (**Table 1**). In these genes, four *TCP* genes were found possessing the R domain. As shown in **Table 1**, these TCP proteins varied in their length, molecular weight, and theoretical isoelectric point. The protein length ranged from 229 to 516 amino acids, the molecular weight from 24.93 to 55.70 kDa and the theoretical isoelectric point varied from 5.38 to 9.51 pH.

**Table 1:** Inventory and characteristics of the *TCP* genes identified in *Prunus Mume.*

Name	Gene ID	Locus	Strand	Length^a^	MW(kDa)^b^	pI^c^	Introns
*PmTCP01*	Pm029002	scaffold205:763920:765428	-	502	55.70	8.16	0
*PmTCP02*	Pm015499	Pa4:19881270:19882614	-	417	46.79	8.48	0
*PmTCP03*	Pm016133	Pa4:23296564:23298211	-	280	29.58	9.33	1
*PmTCP04*	Pm006518	Pa2:17274760:17276031	+	423	44.71	7.00	0
*PmTCP05*	Pm003922	Pa2:1205292:1206842	-	516	53.42	7.21	0
*PmTCP06*	Pm007054	Pa2:21100313:21101122	+	269	28.72	9.51	0
*PmTCP07*	Pm030202	scaffold475:648769:650115	+	448	51.01	6.42	0
*PmTCP08*	Pm018100	Pa5:16193275:16194165	-	296	31.68	7.15	0
*PmTCP09*	Pm010440	Pa3:4466020:4467519	-	499	54.19	7.29	0
*PmTCP10*	Pm023748	Pa7:7860942:7862067	-	332	34.39	5.38	1
*PmTCP11*	Pm029788	scaffold327:681062:682143	+	335	37.09	7.30	1
*PmTCP12*	Pm019399	Pa5:23705247:23706395	-	382	39.69	6.25	0
*PmTCP13*	Pm010118	Pa3:2653873:2654736	-	287	32.61	6.42	0
*PmTCP14*	Pm030138	scaffold475:312338:313027	-	229	24.93	5.98	0
*PmTCP15*	Pm011369	Pa3:10626210:10627529	-	439	47.64	6.79	0
*PmTCP16*	Pm016049	Pa4:22847778:22848908	+	376	41.40	6.59	0
*PmTCP17*	Pm024946	Pa7:15188102:15189265	-	387	42.93	6.95	0
*PmTCP18*	Pm024934	Pa7:15092655:15095383	+	376	40.36	7.46	1
*PmTCP19*	Pm028040	scaffold103:259784:262394	+	463	50.63	6.04	4

*^a^The length of P. mume TCP proteins. ^b^Molecular weight. ^c^Theoretical isoelectric point.*

**FIGURE 1:**
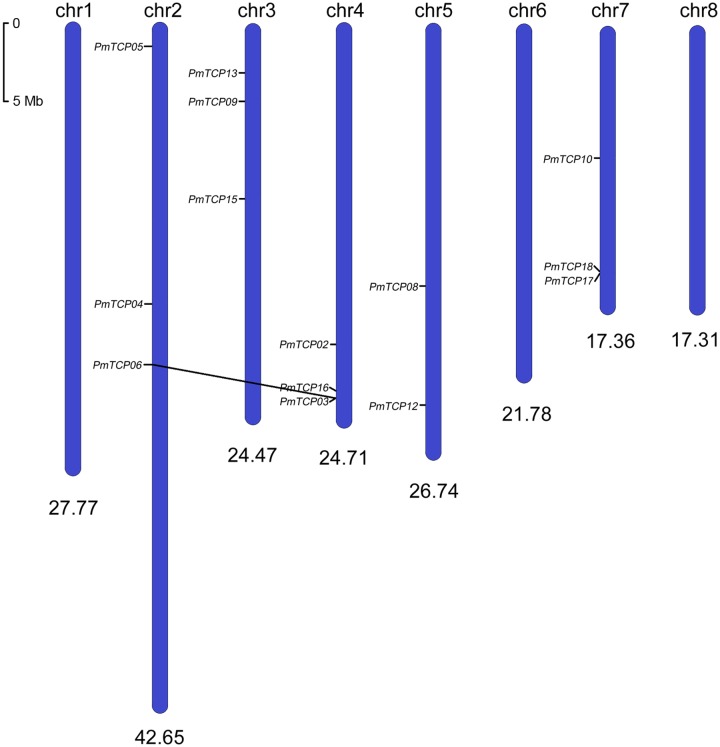
**Chromosomal locations of *TCP* genes on *Prunus mume* chromosomes.** The *scale* showed a 5-Mb chromosomal distance. Segmentally duplicated genes (*PmTCP03* and *PmTCP06*) were linked by *black lines.* There were another five *PmTCPs* that could not be clearly located on the chromosomes, but could be identified on scaffolds.

Based on the genomic data, we located 14 *PmTCPs* on chromosomes (**Figure 1**), but five genes were found on the anchored scaffolds. The *TCP* genes were unevenly distributed over the *P. mume* genome. No genes were located on Chr 1, Chr 6, and Chr 8. Whereas, two genes were located on Chr 5. Furthermore, the Chr 2, Chr 3, Chr 4, and Chr 7 chromosomes each had three genes. Two *P. mume TCP* genes, *PmTCP03*, and *PmTCP06*, were located in duplicated regions. None of the *TCP* genes was tandemly duplicated.

### Phylogenetic and Gene Structure Analysis

**FIGURE 2:**
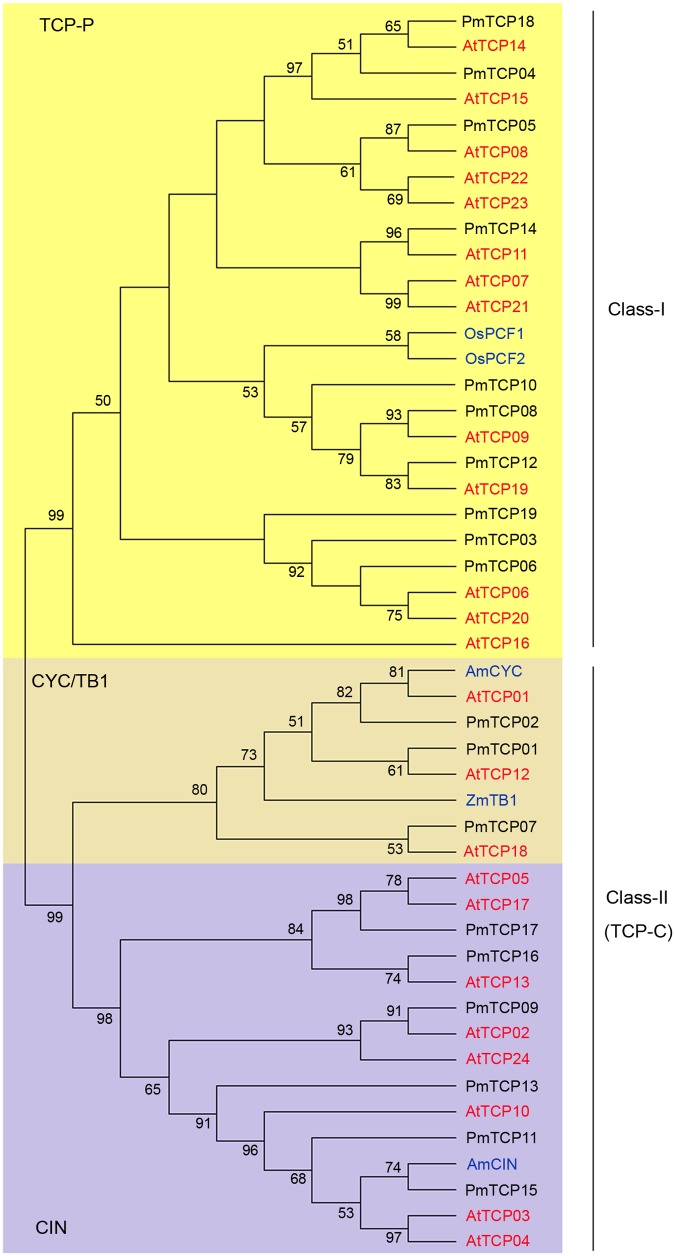
**Phylogenetic tree of *TCP* genes.** The tree was constructed using MEGA7.1with Maximum-likelihood (ML) method. Multiple sequence alignment of the TCP proteins was applied using clustal X2.0. Numbers above branches represented bootstrap value, and the bootstrap values which below 50 would not be shown.

According to multiple sequence alignments of 19 *PmTCPs*, 24 *AtTCPs*, 2 *OsPCFs*, 1 *AmCIN*, 1 *AmCYC*, and 1 *ZmTB1*, two individual subfamilies (Classes I and II genes) were generated using MEGA7.1 with the ML method. Class I is named TCP-P class or PCF class, and Class II is named TCP-C class. The class II genes were further divided into two lineages: CYC/TB1 and CIN. There were 10 *PmTCPs* in class I and 9 *PmTCPs* in class II (**Figure 2**). The *OsPCF1* and *OsPCF2* were found in TCP-P subfamily. Genes of *P. mume* in both class I and class II virtually had counterparts in *Arabidopsis*. These genes first grouped with their homologous genes in *Arabidopsis*. In regard to class II, three of them were CYC/TB1 type genes, and the others were CIN type genes. *AmCYC* and *ZmTB1* were found in CYC/TB1 group. In the CYC subgroup, there were only two *P. mume* genes (*PmTCP01* and *PmTCP02*). *PmTCP01* was gathered with *AtTCP12* identified as CYC3 type gene and *PmTCP02* was gathered with *AtTCP01* identified as CYC1 type gene. The other gene, *PmTCP07*, was grouped with *AtTCP18* identified as BRC1 which was the closest homolog of TB1. In CIN subgroup, *AmCIN* were found grouped with six other *PmTCP*s. The phylogenetic tree suggested that some genes may have undergone species-specific evolutionary processes.

**FIGURE 3:**
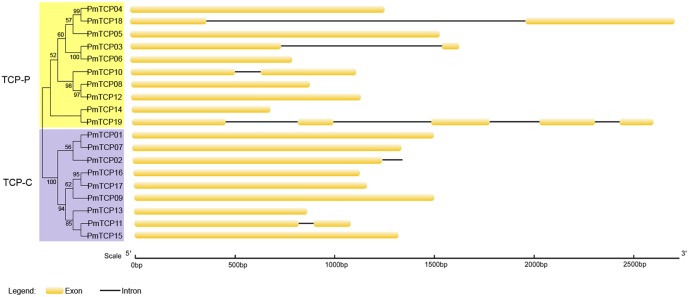
**Exon-intron structures of *P. mume TCP* genes.** Phylogenetic tree was generated based on the protein sequences of *PmTCPs*. Exons and introns of *P. mume* were indicated, respectively, by *yellow rounded rectangles* and *black lines*. The *scale* was referred to the lengths of the genes, exons and introns.

Introns, and especially UTR introns, in *TCP* genes may influence their expression levels (Chung et al., 2006). Based on the genome sequences and corresponding coding sequences of *TCP* genes in *P. mume*, we found that no TCP-C type genes, except *PmTCP02* and *PmTCP11*, contained introns (**Figure 3**). The *PmTCP02* intron was in the 3′ UTR, revealing that it might have a regulation role in the gene expression. Sequences of TCP-P genes contained more introns than those of TCP-C genes in *P. mume*. In the TCP-P genes, the numbers of introns ranged from zero to four. Six of them had no introns, three had an intron, and one had four introns. In conclusion, the exon-intron structure showed itself to be highly simple in *P. mume*.

### Conserved Domains and Motif Analysis

**FIGURE 4:**
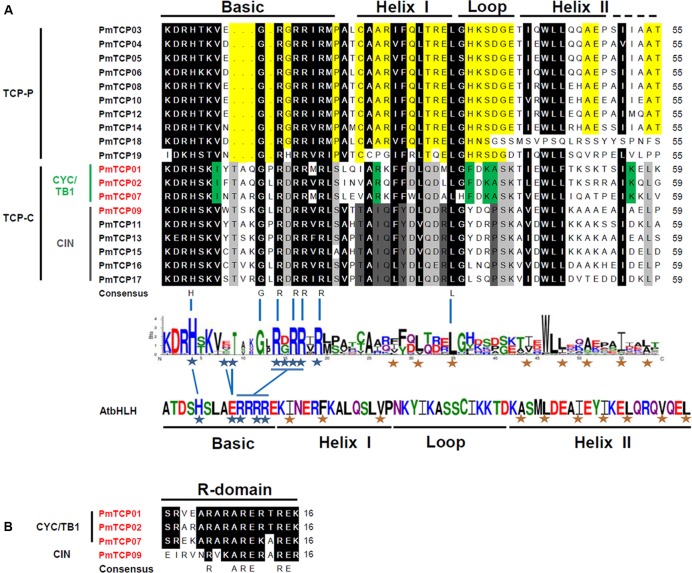
**Multiple sequence alignment of TCP proteins in *P. mume*. (A)** Multiple sequence alignment and protein sequence logo of the TCP domain. Multiple sequence alignment was carried out with DNAMAN. The sequence logo was created in Weblogo online software. The height of the *stack* suggested the sequence preservation at that position. Black boxes showed the conserved amino acids in two TCP subfamily; yellow, conserved in TCP-P; gray, conserved in TCP-C; green, conserved in CYC/TB1 clade; dark-gray, conserved in CIN clade. The residues required for DNA binding were shown with blue asterisks, and hydrophobic residues putatively participated in helix–helix interactions were shown with red asterisks (Manassero et al., 2013). **(B)** Multiple sequence alignment of the R domain.

The conserved domains of TCP protein were examined by Interpro searches and aligned by ClustalX 2.0. Alignment of the TCP domains as well as the dendrogram suggested that the 19 TCP proteins in *P. mume* could be divided into two subfamilies. These *PmTCPs* all contained the TCP domain which is involved in dimerization and DNA binding. The TCP domain in *P. mume* consisted of 55 or 59 amino acids and participated in a basic-Helix-Loop-Helix (bHLH) structure (**Figure 4A**). The hydrophobic residues required for helix–helix interactions in bHLH proteins were conserved in the TCP domain (Manassero et al., 2013). Within the TCP domain, there were several putative residues involved in DNA binding located in the basic region and several putative hydrophobic residues located in Helixes I and II (**Figure 4A**). In the basic region, the TCP-C type proteins contained a 4-amino-acid insertion. The compositions of the loop, Helixes I and II, were quite different between TCP-P and TCP-C. There were four *PmTCPs* containing an R domain (**Figure 4B**). These *PmTCPs* were all classified as TCP-C type genes. Among them, *PmTCP01*, *PmTCP02*, and *PmTCP07* were CYC/TB1 type genes, and *PmTCP09* was a CIN type gene.

**FIGURE 5:**
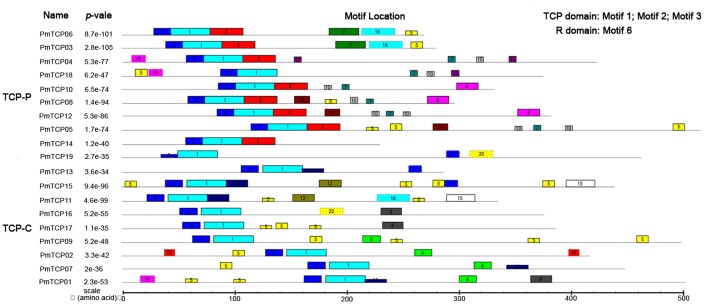
**Motif distribution of *TCP* genes in *P. mume*.** Twenty motifs were indicated using MEME online tool. A *colored block* with a number was consistent with each motif (color figure online). The lengths and positions of motifs in the protein sequences were identified by the lengths and positions of the blocks.

To identify conserved motif and evaluate structural divergences, MEME online tool was used to analyze the motif compositions of the 19 TCP proteins in *P. mume*. Among the *PmTCPs*, the number of motifs varied from three to eight. Three motifs, motif 1, motif 2, and motif 3, were specific to the TCP-domain. As shown in **Figure 5**, motifs 1 and 2 were present in all sequences. Motif 3 was unique to TCP-P subfamily. Outside the TCP domain, there were other conserved motifs specific to clades, such as motif 6 identified as the R motif. Members of the same clade usually had similar motif composition. The duplicated genes, *PmTCP03* and *PmTCP06* belonging to TCP-P subfamily, showed the same motif composition and distribution. Motifs 3, 4, 7, 9, 10, 11, and 14 were unique to TCP-P subfamily, while motifs 6, 8, 12, 13, 15, and 18 were restricted to TCP-C subfamily. Some motifs, such as motif 5, were shared in two different classes.

### MiR159/miR319 Target Site Prediction

**FIGURE 6:**
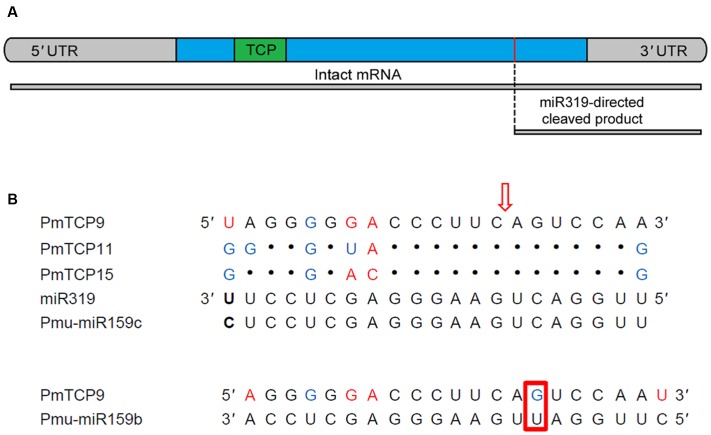
**The miR319 binding sites of *TCP* gene in *P. mume*. (A)** The *TCP* gene structure showed the coding region (blue), the TCP domain (green), the miR319 binding site (red). The putative miR319-directed cleaved product was represented by the short fragment. **(B)** Alignment of miR319 complementary sequences with *P. mume TCP* genes. Mismatches, G-U wobbles and the different nucleotide between Pmu-miR159c and miR319 were represented, respectively, by Red, blue, and black (bold). The direction of the red arrows was indicated the miR319 recognition site in the *TCP* genes. In the red rectangular region was the changed nucleotide of miR319 shifted relative to miR159.

In *P. mume*, three miR159 family members (Pmu-miR159a-c) and only one miR319 (Pmu-miR319) had been identified (Wang et al., 2014b). A result of one miR159 target site (*PmTCP9*) was obtained from analysis in the psRNATarget online software with the full-length of *PmTCPs* nucleotide sequences and Pmu-miR159b sequence. However, three putative miR319-targeted *TCP* genes (*PmTCP9*, *PmTCP11*, and *PmTCP15*) were identified based on the published miRNAs (Supplementary Table S2) in the psRNATarget online application. These three miR319 target sites were all located in the coding regions (**Figure 6A**). And all miR319 targeted genes belonged to CIN clade. Similarly, there were five *TCP* genes containing miR319 binding sites (*AtTCP2*, *AtTCP3*, *AtTCP4*, *AtTCP10*, and *AtTCP24*) in *Arabidopsis*, and all of them were CIN family members. This phenomenon revealed that the miR319 target sites were retained during the evolution and diversification of plants.

The members of miR319 and miR159 were very similar but encoded by different genes and precursors (Schommer et al., 2012). Sometimes they were grouped in one family, whereas others considered them separately (Meyers et al., 2008). In *P. mume*, the Pmu-miR159c sequence was virtually identical to miR319 aside from one differing base located at position 1 (**Figure 6B**). Thus, we considered Pmu-miR159c as a member of miR319 family in *P. mume*. Multiple sequence alignments of the reverse miR319 sequence and three *P. mume TCP* genes showed that the *PmTCPs* containing sequences are complementary to the miR319 sequence (**Figure 6B**). Though a mismatch of miR319 and mRNA came up to six bases, the regulation of the expression of TCP transcription factor through miR319 was considered to be feasible (Schommer et al., 2012).

### Expression Profiles of *P. mume TCP* Genes in Five Organs

**FIGURE 7:**
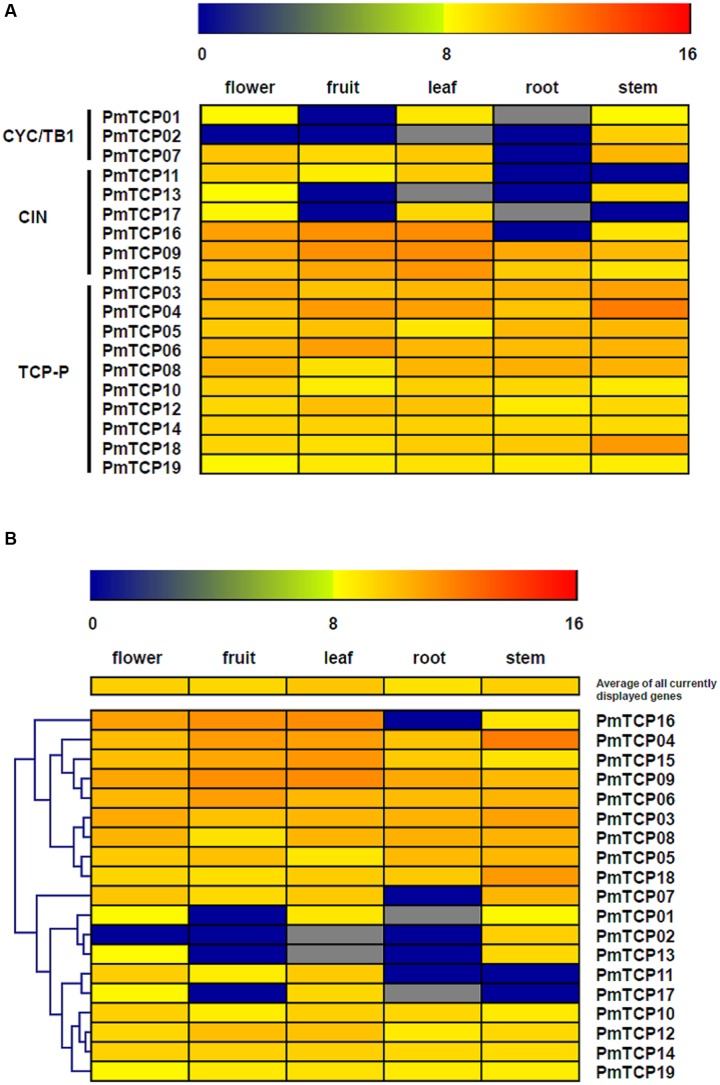
**Expression patterns of *P. mume TCP* genes in five tissues.** Transcriptome sequencing (RNA-seq) was performed to investigate expression profiles of *TCP* genes. The RPKM-normalized log2-transformed counts were represented by the *color scale*. *Blue* suggests low expression levels and *red* suggests high levels. **(A)** Expression profiles related to subfamilies; **(B)** hierarchical-clustering analysis of gene expression profiles.

According to previous studies, the *TCP* genes had key roles in different aspects of plant development. In this study, the next-generation transcriptome sequencing was performed to investigate the expression profiles of *P. mume TCP* genes in different organs and determine their function in organ development. As shown in **Figure 7**, the expression levels of *TCP* genes varied in five organs; flowers, leaves, and stems had the most numbers of expressed genes and the highest expression level. The expression of two genes could not be discovered in leaves and roots, both of which genes belong to TCP-C subfamily. The other 15 genes were expressed in all five organs, indicating that these genes might play roles in the development of diverse organs.

The expression patterns of the different type genes were shown in **Figure 7A**. In general, the TCP-P type genes had wider expression domains and higher expression levels than did TCP-C type genes. All TCP-P genes were expressed in all five organs. This result implied that the genes in TCP-P subfamily played multiple developmental roles in different organs. However, most of TCP-C type genes showed organ-specific expression modes. In TCP-C subfamily, the CIN genes usually had specific prevailing-expression organs, but some of them were expressed in five organs. Furthermore, the genes belonging to CYC/TB1 clade had developmental functions in specific organs. All CYC/TB1 genes were either mildly or not at all expressed in roots, implying these genes might not participate in root development.

A hierarchical clustering was shown in **Figure 7B** and performed to do a further analysis of the organ development in which *TCP* genes could be involved. The *TCP* genes in *P. mume* could be grouped into two clusters according to their expression patterns. Cluster 1 contained the genes whose transcripts were detected in all five organs; but the genes in Cluster 2 did not always have the same expression profiles. The genes belonging to cluster 1 could be divided into two expression groups. One gene formed one group, exhibiting high expression levels in flower, fruit, leaf, and stem. The other group was composed of eight genes and exhibited even expression levels in all organs. Cluster 2 included four expression groups. The first group presented high expression levels in all organs except roots. There were three genes in the second group showing high expression levels in stem and flower. The genes in the third group were strongly expressed in flower and leaf, but their transcripts could not be detected at all in root and stem. Differing from these three groups, the fourth group did not show an organ-specific expression pattern and had relatively stable expression levels in all five organs. The analysis of hierarchical clustering in *TCP* genes showed that some groups from different clusters had similar expression profiles. For example, two clusters had a group of genes which were expressed in all organs.

### Expression Analysis of *PmTCP*s during Flower Bud Development and in Different Flower Type Cultivars

To elucidate their roles in flower development, the quantitative RT-PCR were conducted to confirm the relative expression levels of 19 *PmTCPs* during flower bud development. Based on the paraffin section analyses (Supplementary Figure 1), the flower bud differentiation of *P. mume* was divided into eight stages (S1–S8): undifferentiation (S1), flower primordium formation (S2), sepal initiation (S3), petal initiation (S4), stamen initiation (S5), pistil initiation (S6), ovule development (S7), anther development (S8). Samples were taken from the flower buds of ‘Jiang Mei’, ‘Sanlun Yudie,’ and ‘Subai Taige’ (Supplementary Figure 2). The flower type of ‘Jiang Mei’ is single, while ‘Sanlun Yudie’ and ‘Subai Taige’ are double. ‘Subai Taige’ has a variant pistil which might form a defective flower. All 19 *PmTCPs* were detected during flower bud differentiation in three *P. mume* cultivars, suggesting that most of them play key roles in flower development.

**FIGURE 8:**
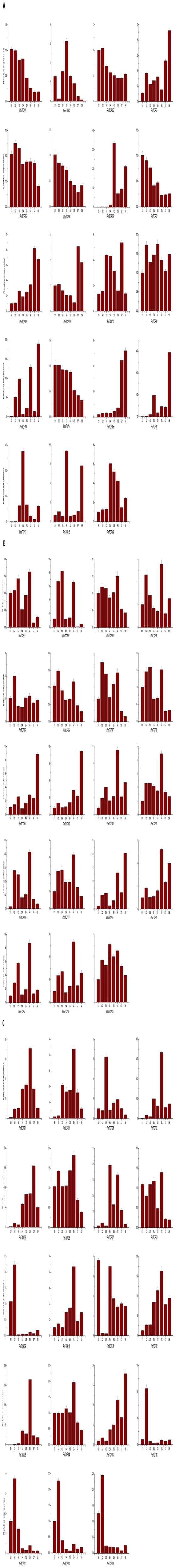
**Expression profiles of *TCP* genes in three *P. mume* cultivars (A–C successively were ‘Jiang Mei’, ‘Sanlun Yudie,’ and ‘Subai Taige’) during flower bud development**. S1–S8 indicate development stages of flower bud. The expression levels of the *TCP* genes were gained by quantitative real-time RT-PCR. The reference gene was the *Protein Phosphatase 2A* (*PP2A1*) gene of *P. mume*. All real-time RT-PCR experiments were employed with three biological duplications, and each duplication was repeated in triplicate. The standard deviation of the results of three technical replicates was shown by the *error bars*.

In ‘Jiang Mei’ (**Figure 8A**), different types of *TCP* genes were quite variously expressed in different stages of flower development. The CYC1 type gene, *PmTCP02*, was significantly expressed in petal initiation. The transcript of *PmTCP07* was highly detected in the stages of stamen initiation and anther development. This result suggests that *PmTCP07*, a homolog of TB1, might participate in regulating stamen development. The CIN type genes were strongly expressed in pistil initiation, ovule and anther development. The TCP-P subfamily, according to the expression, could be divided into two groups. The genes in group one were almost uniformly expressed in eight stages, but some of them seemed to have an unconspicuous function in flower primordium formation and sepal initiation. Others had specific expression in ovule and anther development, or petal initiation.

Compared to ‘Jiang Mei,’ there were also many *TCP* genes with different functions expressed in flower development in ‘Sanlun Yudie’ (**Figure 8B**). The CYC/TB1 type genes, *PmTCP01*, *PmTCP02*, and *PmTCP07*, were predominantly expressed in flower primordium formation, sepal initiation and pistil initiation. There were other genes in the TCP-P subfamily which showed expression primarily in flower primordium formation and pistil initiation as well. The transcripts of CIN type genes were mostly detected in pistil initiation and anther development. Briefly, in ‘Sanlun Yudie,’ the majority of *TCP* genes in *P. mume* were highly expressed in flower primordium formation, pistil initiation and anther development.

In ‘Subai Taige,’ the expression patterns of *TCP* genes were distinct with the other two cultivars (**Figure 8C**). In CYC/TB1 clade, the genes showed high transcript levels in petal initiation and pistil initiation. The CIN type genes were divided into two groups according to their quite diverse expression profiles. One group of genes had high expression levels in flower primordium formation or petal initiation. The others were highly expressed in pistil initiation or anther development. In TCP-P subfamily, there were two kinds of expression patterns as well. Some genes had high expression levels in flower primordium formation and sepal initiation. The others were specifically expressed in pistil initiation.

## Discussion

### *TCP* Genes in *P. mume* and Their Evolution

The TCP transcription factors are ancient plant-specific proteins. Although they have not been found in the unicellular algae, they are present in pluricellular green algae, moss, ferns, and lycophyte (Martin-Trillo and Cubas, 2010). There are five to six *TCP* genes in these plants. Due to the evolution, duplication and diversification of *TCP* genes, the gymnosperms and angiosperms have larger TCP families comprising tens of members. In this study, we identified 19 *TCP* genes in *P. mume* genome.

The analysis of the phylogenetic tree between *Arabidopsis* and *P. mume* supported the earlier described division of *TCP* genes into two subfamilies. According to that division, *PmTCPs* were classified as TCP-P genes and TCP-C genes. Two types of *TCP* genes were distributed regularly on five chromosomes (**Figure 1**). Two TCP-P type genes, *PmTCP03* and *PmTCP06*, were located in duplicated regions. The phylogenetic analysis indicated that the lower number of *P. mume TCP* genes was the result of fewer gene duplication events in *P. mume*. Only for *AtTCP16* can a close homolog not be found in *P. mume*. A similar phenomena was also observed in tomato (Parapunova et al., 2014). The alignment of the TCP domains in *P. mume* showed a similar result to that of the dendrogram. High similarity of proteins within the same clade suggested that gene duplications had occurred after the split of the two classes. Compared with the analysis of the conserved domains, the exon-intron structures of *P. mume TCP* genes exhibitted high conservatism. Generally, genes within a clade shared a similar gene structure, chromosomal location, and motif distribution. However, there were variations in gene structure, motif composition, and miR319/159 target site location among the different clades. Therefore, the classification and evolution of *P. mume TCP* genes might be related to their structural divergence and diversification.

### Expression Patterns of *P. mume TCP* Genes

The expression profile of a gene is always relative to its function (Xu et al., 2015). In this study, we elucidated the expression patterns of 19 *PmTCPs* in five organs by RNA-seq and detailed expression profiles in flower development using real-time RT-PCR. The results showed that these genes had distinct expression profiles in diverse organs, suggesting that *TCP* genes in *P. mume* might be correlated with the development of various organs. We observed a divergence in expression patterns between two subfamilies of genes. In TCP-C subfamily, more than two-thirds of genes had a specific-organ expression. However, all TCP-P genes and some CIN genes were detected in all organs and predominantly expressed in flower, leaf, and stem. The duplicated genes (*PmTCP03* and *PmTCP06*), belonging to TCP-P subfamily, showed a similar expression profile. The expression patterns of some genes were similar to their orthologs in *Arabidopsis*. For instance, eight CIN type genes in *Arabidopsis* (*AtTCP2, AtTCP3, AtTCP4, AtTCP5, AtTCP10, AtTCP13, AtTCP17*, and *AtTCP24*) exhibited high transcript levels in leaf and were responsible for the regulation of leaf growth (Palatnik et al., 2003; Ori et al., 2007). Similarly, the CIN type genes in *P. mume* except *PmTCP13* were expressed strongly in leaf. These genes were also highly expressed in flower. According to RNA-seq analyses, the flower contained almost all *PmTCPs* transcripts, indicating that *TCP* genes in *P. mume* had function in floral organ development.

The *PmTCPs* were expressed during flower bud differentiation, implying that some *P. mume TCP* genes play roles in the different stages of flower bud development. The TCP-C type genes, whether in CYC/TB1 clade or in CIN clade, were involved in the development of petal and pistil. For all three cultivars, the CYC/TB1 type genes were highly expressed in petal initiation or pistil initiation, indicating that they controlled the formation of petal and pistil. In *Antirrhinum*, CYC and DICH have been found to regulate dorsoventral asymmetry and are highly expressed in developing stamens (Gaudin et al., 2000; Galego and Almeida, 2002). The genes in CYC2 subclade take part in the formation of floral zygomorphy (Yang et al., 2012). Furthermore, CYC genes may be related to a change of petal size in *Arabidopsis* (Costa et al., 2005). The less-complex flower types of *P. mume* might result from the fact that there were only two CYC type genes present in *P. mume* and a lack of CYC2 genes to control the identity of flower types.

In the single petal cultivar, ‘Jiang Mei,’ the CIN type genes were highly expressed in the stages of pistil initiation, ovule and anther development, implying that these genes were correlated with gametophyte development. In other species, such as *Arabidopsis*, *AtTCP4* which belongs to CIN clade was found to be involved in pollen development (Sarvepalli and Nath, 2011). In the other two cultivars which were all double type, the CIN type genes were predominantly expressed in flower primordium formation and petal initiation. This expression pattern indicated that the CIN genes in double petal cultivars might have more important roles in the formation of petal, which is quite different to their roles in the single petal cultivar.

Similarly, the expression patterns of the TCP-P subfamily genes were different in two flower types of *P. mume*. For the single petal cultivar, the TCP-P type genes might play a key role in flower primordium formation or petal initiation. For the double petal cultivars, most of these genes were strongly expressed in flower primordium formation and pistil initiation. During pistil initiation development, the inner whorl of petals of ‘Sanlun Yudie’ were continual to form a double flower (online resource 3). These results revealed that *PmTCPs* in double petal cultivars might participate in the regulation of the petal formation for their predominant expression in the stages of petal initiation and pistil initiation. In conclusion, genes in TCP-P subfamily have a main role in flower development and gametophyte development of *P. mume*. In *Arabidopsis*, *AtTCP11* and *AtTCP16* participate in gametogenesis as well (Takeda et al., 2006; Viola et al., 2011).

In ‘Subai Taige,’ some genes expression was continued in order to form the petals in pistil primordium for its variable pistils. Contrasting with the other two cultivars, *TCP* genes in ‘Subai Taige’ had particularly specific expression patterns in flower primordium formation, but were hardly expressed in ovule and anther development. The variant of pistil which was replaced by a defective flower in ‘Subai Taige’ might be due to the lack of high expression levels of some *PmTCPs* in ovule and anther development, such as *PmTCP09*.

### Different Expression Patterns of miR319-Targeted and -Non-targeted *TCP* Genes in *P. mume*

Previous studies revealed that over half of the miRNA targets are transcription factors, for instance: TCP, MYB, SBP, AP2, and so on (Rhoades et al., 2002; Aukerman and Sakai, 2003; Martin-Trillo and Cubas, 2010; Xu et al., 2015). Concerning *P. mume TCP* genes, three of them (*PmTCP09*, *PmTCP11*, and *PmTCP15*) contained putative miR319 target sites and one of them had a putative miR159 target site. They were all located in the coding region. In *P. mume*, the miR319 had two differences with respect to miR159: an additional base at their 5′ end but one less base at 3′ end; and a change of C for U at position 6 so that miR319 sequences were shifted one nucleotide relative to miR159 (**Figure 4B**). The same was true for *Arabidopsis*. MiR319 and miR159 in *Arabidopsis* had very similar sequences but regulated different genes. MiR159 did not target the TCPs due to specific sequence requirements (Palatnik et al., 2007). Though miR159 cannot bind TCP transcripts, it has an overlapping role with miR319 in controlling floral development and interacts with each other (Hong and Jackson, 2015). Current studies indicate that some TCP transcription factors were regulated by miR319. The TCP mRNA fragments are generated by a miR319a-guided cleavage and confirmed *in vivo* (Palatnik et al., 2003). In *P. mume*, the miR319-directed cleaved products were predicted in miR319-regulated TCPs (**Figure 6A**).

The miR319s are involved in multiple aspects of plant development such as flower, leaf and gametophyte development (Hong and Jackson, 2015). The miR319-targeted genes in *P. mume*, except *PmTCP11*, were ubiquitously expressed in the five organs examined. All three genes were predominantly expressed in flower, leaf, and fruit, and *PmTCP09* was particularly significant. *PmTCP09* had both miR319 and miR159 target sites.

In plants, the miR319 family participates in the control of flower development with the miR319-TCP model. The defects in petal and stamen development were observed in a loss-of-function miR319 mutant (Nag et al., 2009). Similar to miR159, overexpression of miR319 causes stamen defects and male sterility (Palatnik et al., 2007). During flower bud differentiation, the transcripts that contain a miR319 target site as well as miR319-non-targeted *TCP* genes were all detected in three *P. mume* cultivars. These three miR319 target genes were all involved in petal formation and gametophyte development. In different flower types of *P. mume*, the main phenotype differences occurred in petal initiation, pistil initiation and ovule development. Accordingly, miR319-TCPs seemed to have an important role in the formation of the different flower types in *P. mume*. Nevertheless, the development of flower and gametophyte were controlled not only by simple miR319-targeted genes, but also by different types of *PmTCPs* which lacked a miR319 target site. For instance, *PmTCP04* and *PmTCP10* which were TCP-P type genes and *PmTCP13* which was a CIN type gene were all highly expressed in pistil initiation, ovule and anther development. Therefore, *TCP* genes in *P. mume* might have a coefficient and interactional relationship in the development of flower and gametophyte.

## Conclusion

In this study, we first executed the genome-wide analyses of *TCP* gene family in *P. mume*. Based on phylogenetic analysis, we then concluded that the lower number of *P. mume TCP* genes was the result of less gene duplication events in *P. mume*. Only one segmental duplication event was found in the *P. mume* TCP family. Among the same type of *TCP* genes, the gene structure exhibitted high conservatism. These findings set a foundation for future research on the evolution of *TCP* genes in plants. The expression analyses showed that most of *PmTCPs* were involved in flower development, leaf development and stem development. Otherwise, almost all *PmTCPs* played key roles in the different stages of flower bud development, especially in petal formation and gametophyte development. Compared to the expression patterns of the miR319-targeted and non-targeted genes, *TCP* genes in *P. mume* might have a coefficient and interactional relationship in flower development. Perhaps the most interesting questions which arise from this research are what kinds of interactions take place between different types of *PmTCPs*, and how TCP-target genes are regulated. Further study is needed to answer these questions and provide an improved understanding of the regulatory network related to key *TCP* genes.

## Author Contributions

YZ and ZX contributed equally to this work. YZ, ZX, and QZ designed the whole experiments. YZ wrote the manuscript. YZ, ZX, KZ, WY, TC, and JW analyzed the data. All authors read and approved the final manuscript.

## Conflict of Interest Statement

The authors declare that the research was conducted in the absence of any commercial or financial relationships that could be construed as a potential conflict of interest.

## Footnotes

1 http://prunusmumegenome.bjfu.edu.cn/

2 http://pfam.Janelia.org/

3 http://www.ebi.ac.uk/interpro/

4 https://www.arabidopsis.org/

5 http://gsds.cbi.pku.edu.cn/

6 http://meme-suite.org/tools/meme

7 http://smart.embl-heidelberg.de/

8 http://weblogo.threeplusone.com/

9 http://prunusmumegenome.bjfu.edu.cn/

10 http://plantgrn.noble.org/psRNATarget/?function
