# Supplementary material for: Genome-Wide Identification, Characterization and Expression Analysis of the TCP Gene Family in Prunus mume
Source: Front Plant Sci. 2016 Aug 31;7:1301. doi: 10.3389/fpls.2016.01301 (PMC5005400; doi:10.3389/fpls.2016.01301)
Supplement: Supplementary file 1 [file Table_1.PDF]

Supplementary Material

Genome-wide identification, characterization and expression analysis of the *TCP* gene family in *Prunus mume*

Yuzhen Zhou[1], Zongda Xu[1,2], Kai Zhao[1], Weiru Yang[1], Tangren Cheng[1], Jia Wang[1], Qixiang Zhang[1]*

[1]Beijing Key Laboratory of Ornamental Plants Germplasm Innovation & Molecular Breeding, National Engineering Research Center for Floriculture, College of Landscape Architecture, Beijing Forestry University, Beijing, China
[2]College of forestry, Shandong Agricultural University, Tai'an, Shandong, China

*** Correspondence:** Qixiang Zhang, zqxbjfu@126.com

1 Supplementary Figures and Tables

1.1 Supplementary Figures

Supplementary Figure 1. Flower bud differentiation of *P. mume*. The flower bud development was divided into eight stages (S1-8): undifferentiation (S1), flower primordium formation (S2), sepal initiation (S3), petal initiation (S4), stamen initiation (S5), pistil initiation (S6), ovule development (S7), anther development (S8). The letters had different meanings. FP: Flower primordiu; SeP: Sepal primordium; Se: Sepal; PeP: Petal primordium; Pe: Petal; StP: Stamen primordium; St: Stamen; CaP: Carpel primordium; Ca: Carpel; Sty: Style; An: Anther; F: Filament; Ova: Ovary; Ovu: Ovule; Po: Pollen.

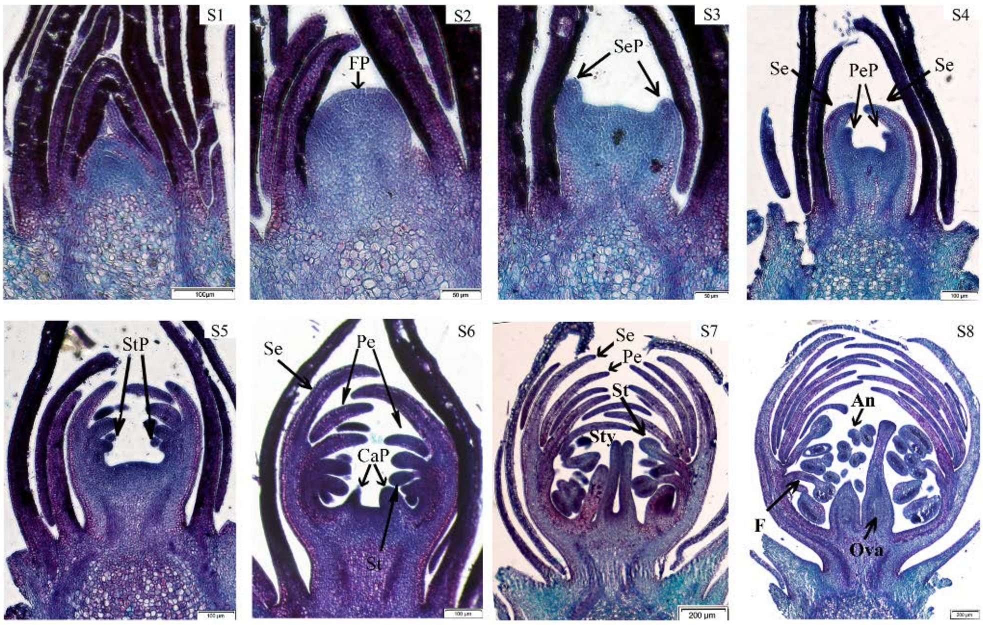

Supplementary Figure 2. Flower of *P. mume*. From figure 1 to figure 3 successively were 'Jiang Mei', 'Sanlun Yudie' and 'Subai Taige'.

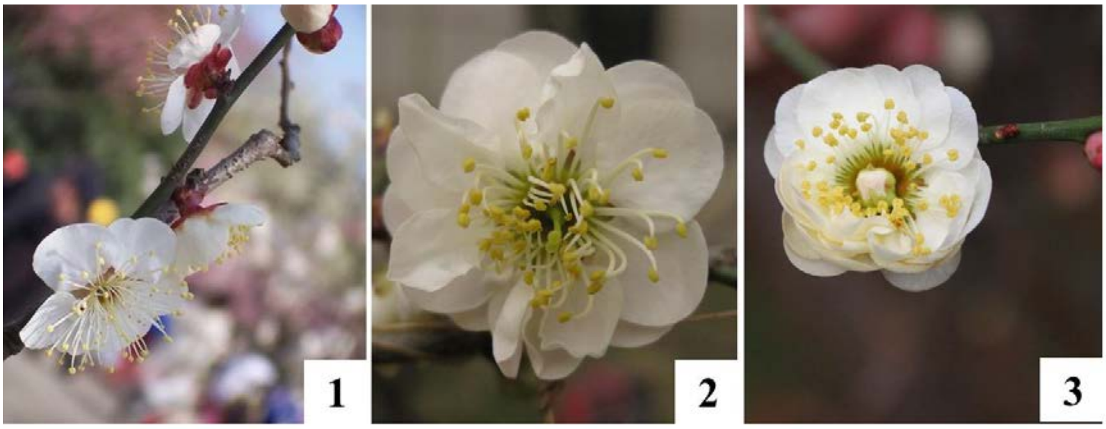

Supplementary Figure 3. The alignment of *TCP* genes in *P. mume* and other species.

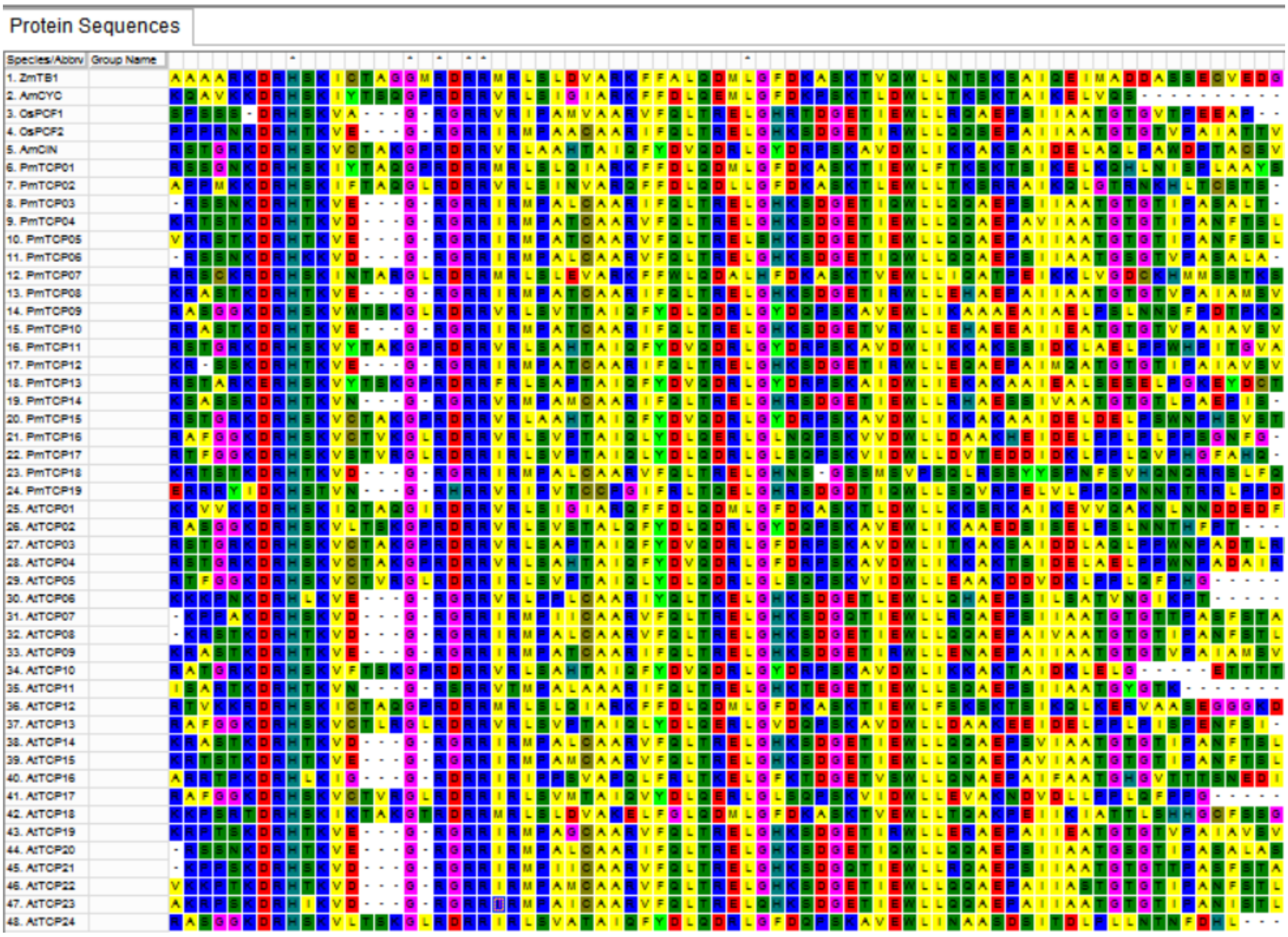

1.2 Supplementary Tables

Supplementary Table 2. Primers used for real-time quantitative RT-PCR.

Gene	Forward primer	Reverse primer
PP2A1	5'AGGGTTCGGCTCGCAATAATAGA3'	5'TGTTAGCAGCAGCATCACGAAT3'
PmTCP01	5'TGATGAGAAGGTTGTGGGCA3'	5'GGGTATGAGGTAGGAGGAGC3'
PmTCP02	5'AAGAGGCTTGTAGTGGAGACA3'	5'TTGTTAATGGCACCATAGTAA3'
PmTCP03	5'CCTCAATTTTGGGTGGTGGG3'	5'ACTGTCCTGAGCCTTGAGAG3'
PmTCP04	5'AGCAACCCAACAACGGCAAGA3'	5'ATGTTGAGGGAGGTGAAGTTT3'
PmTCP05	5'TTCGAATATCCTGCCACCCA3'	5'TGCAATGTACTCCCGTGACT3'
PmTCP06	5'CTCAGTGGGGTTAGGTCTGG3'	5' ATGACCATCCTGTGAGAGCC3'
PmTCP07	5'GAGGCTGCAACAAACACCAA3'	5'CCTTGCCTTTTCCCTCGAAG3'
PmTCP08	5'TCTTATGCTCGCGCCCAAGGA3'	5'GTGCTCCAAGAGCCACCGGAT3'
PmTCP09	5'TTTGTTCCCGACCACCTCAT3'	5'GGCTCCGATGAAGAAGGGTA3'
PmTCP10	5'ATTGACTCCGACCCTCCGCCG3'	5'TGTGGCCCAGCTCCCGGGTTA3'
PmTCP11	5'CACCAACCTAAACCCTTGCC3'	5'CTGTGGCAATGCTTGTGAGT3'
PmTCP12	5'TTGTCGGCTTTTCAACCAGG3'	5'CTAGCACCAGTCCCACTACC3'
PmTCP13	5'TTCAGCATCACCAGAGAAGC3'	5'CTTCTCCTCCATCACCTGCA3'
PmTCP14	5'CAACCGAATCAGCCGCATC3'	5'CCAAGACACAGAGCCTCCT3'
PmTCP15	5'TCTGCAGCATCAACAAGCTC3'	5'GTATCTCCACCACCAGCACT3'
PmTCP16	5'GTTGCCCTCCACATTGTCTT3'	5'CAGGGTCTGGCTATTAGGGT3'
PmTCP17	5'CAACCCGGTAGTGGAGAGAG3'	5'ATGGTCCTTGTTTGGTTGGC3'
PmTCP18	5'GTGGAGGTGGTGGAGGATAC3'	5'CCCTCTATACAACCCGCTGT3'
PmTCP19	5'TGGTTATCCCCGTGGTTTGA3'	5'CTCGCTCCACTACTCCCATT3'

Supplementary Table 2. The sequences of miR159 and miR319.

Family	miRNAs	miRNA sequence (5'-3')
miR159	Pmu-miR159a	TTTGGATTGAAGGGAGCTCTA
	Pmu-miR159b	CTTGGATTGAAGGGAGCTCCA
	Pmu-miR159c	TTGGACTGAAGGGAGCTCCTC
miR319	aly-miR319c	UUGGACUGAAGGGAGCUCCUU
	aly-miR319d	UUGGACUGAAGGGAGCUCCUU
	ath-miR319c	UUGGACUGAAGGGAGCUCCUU
	bdi-miR319b	UUGGACUGAAGGGUGCUCCCU
	cme-miR319c	UUGGACUGAAGGGAGCUCCU
	cme-miR319d	UUGGACUGAAGGGAGCUCCU
	gma-miR319f	UUGGACUGAAGGGGCCUCUU
	gma-miR319g	UUGGACUGAAGGGAGCUCCUUC
	gma-miR319l	UUGGACUGAAGGGAGCUCCUUC
	mes-miR319f	UUGGACUGAAGGGAGCUCCUU
	mes-miR319g	UUGGACUGAAGGGAGCUCCUU
	cpa-miR319	AUUGGACUGAAGGGAGCUCC

2 Supplementary Data

Supplementary Data 1. The CDS sequences of 19 *P. mume TCP* genes.

>PmTCP01
ATGTTTCCTTCTAGCAGCAGCGCTAGTAATTACCAGCTGCCTTTTCCATGTGATACCAAC
CAGCAGCCGCAGCTGCTCGAAAAGTCCTCCACAAATATCATTGATCAACATGGTCATG
AAAACCCTAACTCAATATCACATGATGACCATCATATTCGTCAGTACTATTCACACTACT
CTGAAGATCATCAGCAGCAGGCACCTAATAATTTTCTTGAACACGACGGGCTGCTTTTG
AGCTACTTGCTATCTCAGCAGCAGCTCTTGGTTGGCAGCTCCAGTCCCAATATGAACAG
TGCTACTTCTCATCATGTTCAAGTTCATGATACTACTGAAATCAGCGTTGTGGCTTCCAA
TTCAAACAAAAAAGTAATGGATCGTGTTGACGAGGGTCGGACTGCCCCAGCTGCAACT
TCGAAAGGGTGCTCTTCAAAGAAGAAGAGTAATACATCAAATGGAGAAAGCAAGAAC
CCGAAGGCTCCTCGAAAGAGATCAAGCGGGAATAAAGACAGGCACAGCAAGATCTAC
ACAGCTCAAGGCCCAAGAGACAGAAGAATGAGGCTGTCTCTTCAAATTGCCCGCAAG
TTCTTTGATCTTCAAGACATGCTGGGGTTTGATAAAGCCAGCAAAACCATCGAGTGGCT
ATTCACAAAGTCCAAGACTTCCATCAAGGAACTCAAGCAGCACCTCAACATCTCACCC
CTGGCAGCTTACAGCTGTAGCACCAATGCCAACGCTACTTCTACCAGCACAATCACTG
CAAAGATGAATTCGTCCACTTCTGAGAATAGCGAAGTTGCATCCAAGATTCAGGAGAC
TGCAAATGGCGACGTACACTCGATTGGGACATTGGAGAGAGATAAAAAGAACAGGAA
GTTATGTGTGGTTGCGAGAGAATCGAGGGTCGAGGCAAGAGCAAGGGCAAGGGAGAG
AACAAGGGAGAAGATGATGAGGACCAGATCAGGTTTGGATCAATATCAGAATTTAAGA
AAACAAAGCCCTGATCATGATCAAGTACCACAAAACCCTAATAATGATCAATTCTCGGG
CGGTCACTACTTATGGTCCTCTAATTGTCCTAATGATCAATTGTTTGAACCTGGAATGAT
GATGAATTCCATGGTGATGAGCATCAATCATCATGATGAGAAGGTTGTGGGCAGCACA
ACAATTCCCAGTGGAGCAAATTCCGAGGATTATGATTTTCCAAGTTTTCCTGGGAATTG
GGGCCAAATCAACAATTCCAAGAACATTACAGGTAATAACGTGCTGCAAGTAGATCCA
AACCCTAGTAACTCATCAACTATGGCTACTGCTCCTCCTACCTCATACCCCCTAGAACA
AAAGCCTAGTTCAGTTTTCATGTCCACCTCATCAAGCATGCACGAACAAAACCCTAGTT
CAATCTTCGTCACAACCTTGATTGCTGAAGACCAAAATCCCACTACTTCTTCAAATTTT
GGGACCAATTCAAGTATTGTTTTGAAGTCCCATTTCCTTGGAAATTAA
>PmTCP02
ATGTTTTCATCTTGCAGTAGCAATATTAATAGTAATTCTGTAAGTCCTTTTCAACCTTACT
TTCCCTTGTCTTCTTCAAATTACCACCCTCCTCCTCCTCCCCCTCCTCCTCCTTTTCCTTG
TGTGAACCAAGAGGCTTGTAGTGGAGACATTTTCCTTCACCACAATATTCAAGGGTACC
CCATCTCTGGTCAATTTCCACTTCATAACAATGCCTTAATGGCACCTCCTCTTCCTCAAA
GCTTGACCCATTTGGGAGTTTCATCAAACACTGTACCTCCTAGCATAAACGCTGATGAT
CATCATCACTACCATAATTACTATGGTGCCATTAACAATGATAATATTTTTCCTCATTTCCT
TCATTCTTCTAGAGAAGATATAGTAGCGCCGCCGATGAAGAAAGATCGGCACAGCAAG
ATCTTCACTGCTCAGGGCTTGAGGGACCGGAGGGTTAGACTGTCCATCAATGTTGCCC
GTCAATTCTTTGACCTCCAAGACCTGCTAGGGTTTGACAAGGCAAGTAAAACCCTAGA
ATGGCTTCTCACCAAGTCAAGAAGGGCCATCAAACAGCTTGGGACTCGGAACAAACA
CTTGACCTGCAGTACTAGTACTGGGCGTTCCAAGAGTTTGACTTCGAGTTCAGAGTGT
GATGACGATGACGTCGATTCAGACACCAACGAGGTGGAAAACGTAGCAAGCAAAGAA
AAAGAGGTGATGTTGATGATGAAGAAAAAGATGAAAGAATCTGAATCTGCTAATGTTT

ATGGCACGAAAGACTCGAGGGCAAGGGCAAGAGCAAGGGCAAGGGAAAGGACAAG
AGAGAAATTAATGTGCACTGGGAGCAGGCCTCAGATGTTGAACCAATTGAAGTTATTC
AATGAGCTTGATCACCATCAATCTAACAATAATTGTAAAACAATGTCATCATCTTCAGCA
AAGGCCAATATTGGAGATCATCCTGAGAATCAGGAACTGGGCTCCCTCCTAGCTAATCA
ATTAGCTCATCATCATGAGGACGATCCGTCTGTTCATGTCATCAAAAGAAACAAGTTGA
AGCAGTATTCATCAGTTTATTCCAATTATAATCAGCAAAAGTATCTCAATGTGGTGTCAG
CAGAAAATACAGATCAGAGTTCCAACAGCCACATCCAATTTCCCAATGCATTTCAAAAT
TGGGACACCAACGGCGCCTTCCCATGCCCTAACATTCATTGTGCCATTACCAGCATCAA
TCTTTCTACCGGAAATTAA
>PmTCP03
ATGGGCCTAGAGAGCCTACAAGAGTGGGTTAGAAGCAAAGCCATTCATGAGATGCCAG
GTGTCGGAATCTCAAGCATCCAGCTTGGCAACAAAGAAGAGGGGAAGAAGCAGTTGG
CGCCCAAGAGAAGCTCAAACAAAGACAGACACACGAAGGTTGAAGGCAGGGGAAGG
AGGATACGGATGCCTGCTCTGTGTGCAGCAAGAATTTTTCAATTGACCAGAGAATTGG
GCCACAAATCTGATGGGGAAACCATACAGTGGCTTTTGCAGCAGGCCGAACCGTCGAT
AATTGCAGCCACCGGGACCGGTACGATACCAGCATCGGCTTTAACAGCGGCAGGAGGC
TCTGTTTCACAGCAGGGGACTTCTCTATCAGCTGGATTGCACCAAAAGATTGATGAATT
GGGGGGTCCAGTATTGGGTCAGGGAGTAGGACCAGTTGGGCAATGGTAGGTGGGAA
TTTGGGGAGACCCCATGTGGCCACTGGGCTATGGCCCCCTGTCAGTAGCTTTGGTTTCC
AGTCATCATCTGGTCCATCAACAACAAATCTGGGCAGTGAGAGTTCAAATTACATGCA
AAAGATTGGCTTTCCTGGCTTTGACTTGCCTGTCTCCAACATGGGTCCTATGAGTTTCA
CCTCAATTTTGGGTGGTGGGAGTAACCAACAGCTTCCTGGCTTGGAGCTTGGGTTGTC
TCAGGATGGTCATATTGGGGTTTTGAACTCACAAGCCTTGAGCCAGATTTACCAGCAG
ATGGGGCATGCTAGAGTACACCAGCACCAGCACCAGCACCAGCACCAGCAACCTCCT
GCTAAGGATGACTCTCAAGGCTCAGGACAGTAG
>PmTCP04
ATGGAAGCTGAAAATGGGATCCGCTGCAGGCCCAATTTTCCACTACAATTTCTAGACA
AAAACCAAGATGATGACATCCAAGAAGCGCCAGGAGACCCGGGTTCCAACCAGAACC
GACCCGGCTCCTCCCTGACCCAAGAGCAGCAACCCAACAACGGCAAGAAGCCTCCTC
CGAAGAGGACCTCCACCAAGGACAGGCACACAAAAGTGGACGGGCGAGGCCGGCGG
ATCCGTATGCCGGCCACGTGTGCCGCTAGGGTTTTCCAGCTCACGAGGGAGCTAGGGC
ACAAGTCGGACGGTGAGACCATTGAGTGGCTTCTCCAGCAGGCCGAACCCGCCGTCA
TCGCAGCCACCGGGACCGGCACCATTCCCGCAAACTTCACCTCCCTCAACATCTCGTT
GCGGAGCTCCGGCTCTAGCATGTCGGCGCCGTCGCACTACTTGAGGCCAAACAGCAGT
AATTATTTCATGAACTTCAATCCCAACAACTTGGGTGCAGCAGCTGCGTCGCAGCTGAT
TATGGACGAGAACTCGCAGCGCGGCAGGATTTTGTTCCCCGGCGTTGGGTTGTTGTCG
TCTGAGAGCTCCCCGTCGTCGTCTATGTTGTTGAATTTCAACAATGCCATGTTGGAAGC
CAAGCGCGAGTTACGTGATCAGGGCGGTTCTCTCGGTGAGGTGTCCGAGGGCGGCGA
GGCCAGCACCATGGGAAGGAAGCGAATTAGGCCGGAGCAAGATTTGTTATCGTCTTCG
TCGTCCTCGTCCTCGTCATCACAAAGTAGTCATCATCAGATGGGGATATCGAACAGCTA
CATGTTGCAATCAGCCAGCACGGGGTCAATTCCGGCGAGTCAAAGTACAATTCCGGCG
ACATTTTGGATGGTGGCAAATAACCCTAGTAGCGGCAGCGGAGGCGGCGGTCATCATG
TCCATGAGCCTCCCATTAATCCCATGTGGACAATTCCTAGTATTAATAATGGTATTAACA
GCAATATGTATAGGGGGGCTTCGGTGTCTTCTGGGGGTGGGCATGGTCTGCATTTTATG

AATTTTGCTCCACCTCCAATGGCGGCCATATTACCTAGCCACATGGGATCAACTCCTCA
CGGCGGTGGAGGCTCAATTGCCGACAGCCACTTGGGTGTGATGGCTGCGCTCAACGC
GTACAGGCCGATTCTCGGTGGGGTCATGGCGGAGCCACCGGGTGGGAATAATGGACAG
CATTCACACCATGGAGCAGACGAGGGACATGATCGTGACAGCAATGCTAGGTAG
>PmTCP05
ATGGGCTCCATCTCAATTCAAGCTGGCCTCACCTCTACTCATACACCGACAACAACTTC
TGCTACTGTTTCTGCACAACCACCAGCTCCTTCCTCCTCTTCTTTGTCTTCTTCCTCCTC
CTCCTCTCCCTCCTCCACCTCCACCTCCACCGCTCCTCCTCCCCACTTGGTCGACGCCT
CCCTCGCCATCGCCACCAGATCCGACTCCCACCCCTCAACCCGACCAGCCCCGCTCCT
CGACTCCGCCAAGAAAAACCAAATTCATCAGCAGCAATTGACAATTTCACCCACCAGT
ACTACTACTGCGACACCACCAGCACCACCAGCCGCAGCAGCAGCAACCCAAGTAGTG
AAACGATCCACCAAGGATCGGCACACGAAAGTCGAAGGCCGAGGCCGCCGCATACGA
ATGCCGGCCACCTGCGCCGCCAGAGTCTTCCAGTTGACGAGAGAATTGAGCCACAAG
TCCGACGGCGAGACAATCGAGTGGCTCCTCCAGCAAGCCGAGCCTGCGATCATCGCCG
CCACCGGAACGGGGACCATCCCGGCCAATTTCTCCAGTTTGAACATATCCCTGCGTAGC
AGCGGATCAACGCTCTCCGCTCCGCCTTCCAAGTCCGCCTCTCATTCCTTTCCCGGCGC
CTTGGCTCTTGCCCATCACCCTCACTACGACGAGGGTTTTCCCCATTCGGGTTTGTTGG
GGTTTCATCAACAGCAGCATCATCACCATCATCATCAGCAGCATCAGTCTCCTCTTATGA
CCGCGGATCAAATTGCAGAAGCGCTTCCAAGCGGCGGCGGAGACAGCGGCGGTGGAG
ACTCTACCGACAGTTATATGAGAAAGCGCTTCAGAGAAGATCTGTTCAAGGATGATAAT
CAAGGGCGAGGCGAAGGCGGCGGAGGCAGCAGCGGAGGAGGAGGGTCGCCGTCTGG
GAATAAGGCTTTTAAGAGCAGCGGAGGCTTACAATTGCAAAAACAACAACAGGGGTC
GGAGGGGGAGGCAGGACCTTCAGGGCTGCTGAGGCCAACTTCGAATATCCTGCCACC
CACTGCCATGTGGGCAGTGGCACCCGCCCCGAACAGCGGTGCTGCTGGCAGCACATTC
TGGATGCTGCCAGTAACCGCTGGTGCTGGTGGGCCCTCAATGCCCACCGGGACGTCCG
GCGCTGGTCCTTCCGAGGCTGCACAGATGTGGACGTTTCCGTCCGCACCTGCCAGTCA
CGGGAGTACATTGCAAGCGCCATTGCATTTCATGCCGAGGTTTAACCTTCCAAGCAGTC
TTGAATTCCAGCAAGCCGGCAGCCGAGGGAGCCCTCTCCAATTGGGTTCCATGCTAAT
GCAGCAACAGCCATCACAGCATCTTGGGCTTGGAGTCGCCGACTCGAATTTAGGCATG
TTGGCTGCTCTGAATGCTTCTTACTCTAGAGGTGGTTTGAATATGAATTCTGAGCACCA
CCAGCAGAATCATCCATTGGATCATCATCATCAACAACAACAACACCAGCCTCAAACTA
CTGATAGTGGAGATGAGGCCCCAAATAGTTCTCAATGA
>PmTCP06
ATGGCAGAGAACAAGCCTGCGCAAATCAAGGACTTGCAGATTCTGAAAGCAAACAAA
GATGATGAAAACAAGAAGCACCTGGCGCCCAAGAGGAGCTCCAACAAGGACAGGCA
CAAAAAGGTGGATGGTAGAGGGAGAAGAATACGGATGCCTGCCTTGTGTGCAGCTAG
GGTTTTTCAATTGACCAGAGAATTGGGTCACAAGTCTGATGGGGAAACAATTCAGTGG
CTGCTGCAGCAAGCTGAGCCATCAATTATAGCTGCCACTGGCTCTGGAACTGTACCTGC
TTCTGCTCTTGCAGCCGCAGGGTCCTCTGTTTCTGAACAGGGGAGCTCTGTTTCCTCTG
TTTCTTCTGGGTTGCATACAAGGATGGAAGGACTAATAAGACCAAGTGTTGGGTCTGA
AGGCAGTGCCAACTGGGCATTTCATATGGGCAGATCTAATGTAGCAAGTGGGGTTTGG
CCCTTGCCCTTTCTCAGTGGGGTTAGGTCTGGCTTTGATCAAAATTCTGGTCAGGCAAC
AGTCAATTTTGGGAGTGAAAACTTGAACATCATGCAGCATAAGTTTGGGTTTCATGGGT
TTGATGTTCCTGGTATGAATTTGGGGTCAATGGGTTTTCCCACAATTCTAAGTGGAAGC

AACCAGCAAGTTCCAGGTTTAGAGCTTGGGCTCTCACAGGATGGTCATATTGGGGTGG
TGAGTTCTCAAGCTTTGACCCAATTTCACCAGCACAAAGGGCAGAGGCCAGGTGGTG
TAGCTTCCTTCAATCAGCAGCAGCAGCATCAGCAGCCATCTGATACGAATGAATGA
>PmTCP07
ATGTTTCCCTCAAACTCAAACAATAATCTCAACAATACTGGCAACGAATTACCCGTGTC
TTATCCTCATGTCGACCAATCCTTTTTCCACAGCCGGCCTTTTCTCCATGAAATCACTAC
ATTAAACCCCAATTCCCTTCATCCCAATCTCAATTCCAAACAAGAAGAAGAACAACGA
CAACAAGAAGGCCCTCATCACCATCCTTTGTCTTTCTTCTACTTCCCCTCTCCTTTAGAA
GACGACGATGTTTTACTCTTCCAACAACACCATCACTATGACCATCAGCATGACCATGT
CCATGACATGCCGCTCCATGACTCACAACAAGCACCACCCCTTACTACTATGAGGGAA
GCTGTTGCTGCTAACAACACCTTAGCCGATGATCATCGTCAGACCACCACCACTTCCAC
TACTCTCAATATTAAAATGGTGGATTGGGATTCCAACAAAAATCATGGCGAGATGATGA
ATATGGATCAGCCACAGATCCCGAGAAGAAGGTCTTGCAAGAGAGACCGCCACAGCA
AGATAAATACTGCCCGAGGCCTCAGGGACCGCAGAATGAGATTGTCGCTTGAAGTTGC
CCGCAAGTTCTTCTGGTTGCAAGACGCACTGCACTTTGACAAGGCCAGCAAAACCGTT
GAGTGGTTGCTCATTCAGGCCACACCTGAAATCAAGAAACTAGTAGGAGACTGCAAA
CACATGATGAGCTCTACCAAAAGCACATCTCCTGCCACTTCTGAGTCATGCGAAGTGAT
ATCTGGCATAGACGAGGCTGCAACAAACACCAACATTCACATTAATATTGATGGGGGA
AATGATGGTGATGATAAACTAATACAAAGCTGCGAGATCCAGCCTTCAGCCAAAGAGA
GAAAGGTTGCCCGCCGGCAATTGTCAAGGAAGACCGCATTTCACCCTCTTTCAAAAGC
TTCGAGGGAAAAGGCAAGGGCAAGGGCAAGGGAAAAAGCAAGAGAAAAGCAGCGG
ACGCATCAAAGAGTAGTTGATGTTGATGATCAATCTAAGAAGCAAAGAGGTGATCAGG
AAAACCTGAGCCGATTGGGTTCTTGGAGCCCCTTTGAAACTGGGGAAGAATCAGCTG
GGACTCAAAGCCACAACAACAACAACATAAGCATCAATTCATTGGAGGGGCTGGTTCA
TCACGAGATTGAAGAACCAATGAGCAGCTGCCAAGTAGGAGATCATCCAGACTTGGTT
GTTGATCATGGAACTACACATGATCCTTTGGTGATTATGGGAAAGTGGAGCCCCCCTTC
AATATTCAGTTCTCTGCAGCAAAACATTGGAATTTCCCAAGAGGTAACTTCCAACAAC
AAGTTTTGA
>PmTCP08
ATGACCATGACTGAGAAACGAGAACTTGAACGAGACCAAGCCACAACGTCCGTCGAT
CTCAGAATCAACGGCGGAGAAGAATCCGACTCAGAAGAGCCCGCCGGCCCATCCCAA
GCTCTTATGCTCGCGCCCAAGGACGAGCGGGACGTAGCTATGCCTGTGGCGGTGCACG
CGCCGAAGAGAGCGTCGACGAAGGACCGGCACACGAAAGTGGAGGGACGGGGCCGG
AGGATCCGAATGCCCGCCACGTGCGCGGCTCGGATCTTCCAGCTGACCCGGGAGCTAG
GGCACAAGTCCGACGGCGAGACCATCCGGTGGCTCTTGGAGCACGCCGAGCCGGCCA
TCATCGCCGCCACCGGCACCGGAACCGTGCCTGCGATCGCGATGTCCGTGAATGGGGC
GCTCAAAATCCCCACGTCCGCTCCAGACCCGAGACCCGGCGAGGACCCGCCCGACAA
GAAAAAGCGCAAGCGGAACTCAAACAGCGAGTACGTGGACTTGAACGACGGCGTTTC
GGCCTCGGCGGGTTTAGCCCCTTTAACAACGGAACGACATCACCAACAGCCACCGAC
AGCAGCGGTGCAAGCGGTGGTACCTCAAGGCGTGCTCCCCATGTGGGCCATACCGTCA
AACGGCGTCGTTCCCGGAGCTTTCTTCATGGTCCCTTCCGCTTCGACCCAGCCTCACAT
ATTTACATTTCCAACCACCGTTGCCGCCGCTCCGTTTATCAACATTTCGGCCCGACCCAT
ATCCTCTTTCGTGGGGCCCAGTTCGGCTTCTGCAACGACCCATATGGCCGCTTCCACCG
CCCCTCAGACGCTCAGAGACTTCTCTTTGGAAATTTACGACAAGAAGGAGCTTCAATT

CATGTCTGGCTCTTCAAACCATTGA
>PmTCP09
ATGGAGGTGGAGGAGATTCAAGCCCAAGCCTGTAAGTTCCCAAGAATTGGAAATGGC
AGTAGCAGAGCCACTAACCCAGCTGCCGACGATGAAGATCAAGACCCATCGTGCCTAG
ACTTCAAGAGGGATACCACTGCGGACGCCGGCAACCGCCTCCGCGGGTGGCACCACT
CCCGCATCATCCGCGTCTCGAGGGCCTCCGGCGGCAAAGATCGGCACAGCAAGGTGT
GGACTTCAAAGGGACTCAGAGACCGGAGGGTACGGTTGTCTGTAACCACAGCCATTC
AGTTCTATGATCTTCAAGATCGGCTGGGTTACGACCAACCCAGCAAAGCAGTGGAGTG
GCTGATCAAAGCTGCTGCTGAGGCCATAGCTGAGCTTCCATCTCTCAACAATTCCTTCC
CTGACACTCCCAAGCAGCTCAGTGATGAGAAGAGGGCGAGTTGTGAGCATGGGTTTG
ATTCAGCTGAGGTTGAGCTCGAGGGGCACGGCCATGGCGACCCCAATTATCATCACCA
TCAGAACCAGAACCAGACTCAGAACCAAAGCCAGTACCTTTCTCTGTCCAAATCTGCT
TGTTCTAGTAATTCCGAGACCAGCAAGGGATCCGGGCTGTCTCTCTCCCGGTCCGAGAT
TCGGGTGAACCGGGTCAAGGCCCGGGAGCGTGCCCGGGAGAGAGCAGCCAAGGATA
AGGAGAAGGAGAGCAACGAGTCAGCTTCACACATTGCTCACCACCCCCAGCAGAACA
GCAGCAACTTGAACAACTCCATTTCTCAAAGCGCTTCGTTCACCGAGCTTCTCACTGG
TGGCATCGGTACCAACAGCAGCAACAACAACAATAGTCCAACCGCAGCTGCTCATCAG
CAGCAGAACCACGGCGGCGGCGGCGGCGAGCCCATTTTGTTCCACAAGGCAGCAGCG
GCAGGAGGAGGGGGACCGATGGATTACTTCAGTTCCGGACTTCTCGGGCTGTCCTCGT
CGACCCGAACCCACCACTCGTCCGGGTTCCCGGGTCAAATCCAGTTGGGGATGAACTC
CATTCCGCAGACAATGTCGGTTGTGTCCCCGTTCAGTGTGTCTGGGGATCATCATCACA
ACCACAACCCGGAGCTTCAGCATTTCTCGTTTGTTCCCGACCACCTCATCCCCGTTACG
ACGTCGTCGCAGCCGGGCAATGGCGGCGACTACAATCTGAATTTTTCGATCTCTTCTTC
CGGCGGCCTTGCTGGTTTCAATAGGGGGACCCTTCAGTCCAATTCTTCCTCATCACCGT
CTCTTTTGCCTCACCACCTTCAGAGGTTTTCTCCCATAGACGGATCATCCAATGTACCCT
TCTTCATCGGAGCCGCGGCGGCTCCAACCATGGAGAACCACCATCACCACCACCACCA
CCATCAGCAACACCAACAGCAGTTCCCAGCTGGGTTTGATCGCCGCTTGCAACATCCC
TACGGCGACGGAAGCCGGCATTCAGACCACAAGGGAAAAGCAAAGAACTGA
>PmTCP10
ATGACGTCGTATTTGGAGGATCAAGACGACGACGGAGGCACCTCCGATCTCAGCACCA
GCACCGGCGACCCCGAAGACAACAACAACAACGGAAACGGGGTCGTTTCGACGCAA
CCCAACTTCGACGAGACGACGGCGTTTCAGCAGCTGAAGGAGGAGCCCATTGACTCC
GACCCTCCGCCGCAGGCTCACTCCATCGGCATGGTTCCGGTGGCGATGCAAATGCCCA
TGTCCGTGGCTGTACCCGTTTCCAACCCGACCCGACGGGCCTCTACCAAGGACCGCCA
CACGAAAGTCGAGGGACGCGGGCGGAGGATCCGAATGCCCGCCACGTGCGCGGCCCG
GATCTTCCAGCTAACCCGGGAGCTGGGCCACAAGTCCGACGGTGAAACCGTCCGGTG
GCTCCTTGAACACGCCGAGGAAGCCATCATCGAGGCCACCGGCACCGGCACCGTTCC
CGCCATCGCCGTCTCCGTCGACGCCGTTTCGCAGTCGTCGGGTCTGGCTCCGGTGGGG
CCCGCGGCGCCACAAGGCCTGGTTCCGGTCTGGGCCGTGGGCGGCGCCGGGCTGATG
GTCCCAGCGAATGCTTTCTGGATGGGCCCGGTGGGCTCGGGTGGGGGACCGTCCGGCC
CACAACCGCAGATATGGGCCCTGTCGCCGACGGTGACGCCGGTTTTCAACGTGGCTGG
GGCTACCCGGCCCGTATCGAGTTTTGTGGCGAATAACGGAGGAGGAGTGGAGGTTCGG
GCTCCGTCGCCGGCACTGTCAAACTCAGCGGTGAGTACGAGCACGGTGGGGCCGAGG
GCGGCGAAGAGGTCGTCGACGACAATGGCGCCGAGCGTGAGCTCGTCGAGTAACAAC

AGTAACGGTAGCGGGAGTGGCGCGAGCAAGGCTCAGATGCTGAGAGATTTTTCTCTTG
AAATTTACGACAAGCAGGAGTTGCAGTTCATGGGTCGACCCGTCGGCTCGCCAACAAC
TCATCAACATCAAACCCAGTGA

>PmTCP11

ATGGGAATGAAGAGCACTGGAGGAGGAGGAGGAGGAGAGATTATACAAGTTCAAGGA
GGCCACATTGTTCGATCCACCGGCCGCAAAGACCGCCACAGCAAGGTCTACACCGCCA
AAGGCCCCCGAGACCGCCGCGTCCGGTTGTCTGCCCACACCGCCATCCAATTCTACGA
TGTTCAAGACCGCCTCGGCTATGACCGCCCCAGCAAAGCGGTTGACTGGCTCATCAAG
AAAGCAAAATCCTCCATTGACAAGCTTGCTGAGCTTCCTCCTTGGCACCCTATCACTGG
TGTTGCAGCAAACAATGCTGAGCCCGATCAATCCAATCCAAATGAAATGGTGATTGCA
GGAGGAGCAGAGGAAACGGAGTCCTCTGGCTACAATTTCCACCAGCTGCAGAGGCAA
ATGGGTGAGAATAACAACAACCAAGCAAATAATGTTTCAAGCTTCAACATTCCTCCATC
TCTAGACTCAGACACCATAGCTGACACCATGAAATCATTCTTCCCCACAAGCTCAGCA
GCCAATTCATCCATCAATTTCCAAAGCTACCCAGCGGATCATGATCTGATTTCAAGAAC
CACCAACCTAAACCCTTGCCAAGACCTTGGCCTCAGCAATCCCAAGGGGACACCAAC
CACAACAACGATCAGATTCAGACCCTTTTCGCCGCGGGGGCGGGAGGATCAACAGTG
GGATTTGACACTAGTTATCAGAGAATGGTGGCATGGAGCAATCAGAACAGAGGAGGCG
ATGGTGGATTTGTATTCAACTCACACTCACAAGCATTGCCACAGCAGGCATATGCTCAT
GCTCAGGGGGCAGAGAACACAGCAGCAGCAGCAGCCAATGATTCACCATTCTTCAATC
TTTGGCACCCGCTTCGCGTCTGATGGCGGCTTGCCAGTGTTCTGCATCCCCACGCGTAT
TGACGCTGAGGAGGCCGATAATGGCGGTGTTTCAGATCGACCATCATCCACTTCCTCTC
CCAATTCCACCCACCATTGA

>PmTCP12

ATGGAACCGAATCAAAGGCAGAGCCTTGAAGAAAGCAACGAGCTTGAACAGCAGAG
CAATAGCAGTAGCAACGACAACACCACCTCAGCATCCGACCCATCAGTGGCGGACCCT
CCGGAGAAAATATTTCCCTTGACGGCGCCGACGATGAAAGAAGAGCTGACGGACACA
GTGCAAGAACTGGACGAAGGATCTCTGCCTATGGGTCTTATTCAGGTGCCGGTTCCTAC
GTCGTCGGAGAAACAAGTGGTGGCGGCAAAGAGGTCGTCGAAGGACCGCCACACGA
AGGTGGAGGGCCGGGGCCGGAGGATCCGAATGCCCGCCACGTGTGCGGCCAGGATTT
TTCAACTGACCCGAGAACTTGGCCACAAGTCCGACGGCGAAACCATAAGGTGGCTGC
TCGAGCAGGCCGAGCCTGCTATTATGCAGGCCACGGGCACAGGCACAATCCCCGCCAT
TGCCGTCTCCGTCGGTGGCACTCTAAAGATACCCACAACGTCACCGGCGAGACCCAAC
GGTGAGATTACTGAAATACCCAGAAAGCGACGGAAGAGAGGGTCGAACAGTGAGTTC
GTTGATGTGCATGAGCAGAGCTCTGTGTCTAGTGGGTTGGCCCCAATGAGCTATGGCG
GCGGTGGCGGTGGCGGTGGGGCACATGGGTTGGTGCCCATGTGGCAAGTTGGGGCAA
CTGGTGCAGCTGGGCCTTTCTTCATGTTCCCAAACAACGGGGCTGTGAACCCAAACCA
GCCCCAGCTTTGGGCTGTTCCAGCTGCGGATGCTGCAACCCCGATTTTCAATTTCCAAG
CAAGACCCATCTCCAATTTCTTGTCGGCTTTTCAACCAGGTGTTCACGTTGTTGGTGGG
GATGTGCAGTTGCAGGCCTCTTCGGGTTCGATTTCGAGCGGTGCAACTTCGGGTTCGG
GGGGGAGCTGTTCGTCTAGTTTGGGACCCAGTTTGGGCTCCGCCTCAGGAACCAGAGC
TAATAAGAACACCATTTCTACTGGTAGTGGGACTGGTGCTAGTGCTGCTGCTGCTGCTG
CTAGTGCCACAACAACTCAGATGTTGAGGGATTTTTCTTTGGAGATTTATGATAAGAGA
GAGCTTCAGTTCATGGGTGCAAATTCACAAACACCATACTCTAAGCCTTAG

>PmTCP13

ATGAGTCACCTGCAAGACATTCTGCGTCTGCAAATGTTACAACAGAGAGGATCAAAGA
ATGAAGATCAAGATCAACCAGAAGATGTTCAAGAAGAAGAACAACAATCACAGAAGA
GACTAATTGGTCAATATCAGCATGTTCAAGAACCACCAAACTATGGACCATTAAATGGC
AAGATGTTGAATACCCACATAGCAAAATCCTCCAGGAAAAGTTGTTACTCAATGTCTTC
TTCTTCTTCACATTTGGCATCTGAACAAGCGAAAATCAACAATGCGAGATATGGGAAG
ATTGTAAAAGTTCATGGAGGCCACATTGTCCGATCCACTGCAAGAAAAGAAAGGCACA
GCAAGGTGTACACTTCCAAAGGTCCTAGAGACCGGAGGTTCCGGCTGTCAGCTCCGA
CTGCTATACAGTTCTATGATGTCCAAGACCGCCTTGGCTACGACCGGCCAAGTAAGGCC
ATTGATTGGCTCATTGAGAAGGCCAAGGCAGCAATTGAGGCTCTCTCTGAGTCTGAAC
TACCTGGAAAAGAGTATGACTGTACTAATATCAACAACTCTGCTCAACAGACAGAGCA
AGACATTGGAGAAGAGAGTATGCGTCAGTTTCAGCATCACCAGAGAAGCTATGGTGGT
GAACCAGAAAAACTGAACAATGTGAACAGTTTTAAAGAACCAGTTCTTGATCACTACC
AGTTAAGTTCAATGAACTATGCTGAAGAAGCCCTCAATTCTGGCTCAAGTTTAACAGAT
TCCAAAATGGAGGTGGCTTGGTTTCAAAGTTTGTTGGCTTGGAATTATAATGCAGGTGA
TGGAGGAGAAGGCTGTCCTTTCAATTCATCCCATGTATATCTGCAATAG
>PmTCP14
ATGGGCTCAGAGATGGCCCTTGCCCCTCTACGCAACCACGAAGACCCGACCCTCATCC
CCACTGCCGTTTTGTCCTCCGCCGCCCTAGAAACGGCGTCGTCCCGTCAGCACTCCCA
GCAATCCATACAATCTCTACAGCCCAAGACACATAACCAGCTCTCTCAGACCCGAAAG
TCCGCGTCTTCCAGGGACCGCCACACCAAAGTCAACGGCCGGGGACGGCGAGTCCGG
ATGCCGGCCATGTGCGCCGCCCGGATCTTCCAGCTGACCCGGGAGCTCGGCCACCGCT
CCGACGGGGAGACCATCGAGTGGCTCCTCCGCCACGCCGAGTCCTCCATCGTCGCCGC
CACCGGTACCGGCACTCTCCCGGCCGAGCCGATTTCGACCTCCGCCCCCGCCGTCTCC
TCCCAAGTGCCCTCCCTCGCTTGCCGGGCCCACCCGCTCAGCTCCCTCAATGGCGGCG
GGCAGTTCATGTACCCGCTGGTGTCGTCGGCTCATCAGGCTCAACCGAATCAGCCGCA
TCAGCAGCCGAGCATTCGGCTGGACTTGTGCCAGCCGGCGGGGTTGGACTACACCGA
GTACCGGCAGCACATGCCGTTCACGTCGTTGCTGCTGCATCCGGCGGAGAATGAGGAG
GAGGAGGAGGAGGATGGGCAGCAGGAGGAGGCTCTGTGTCTTGGGGATGGATAG
>PmTCP15
ATGGGCGACACCCACCACCACCACCACCCCCAAGCAACGACGTCGTCCAGACTGGGG
ATAAGGCCGTCTTCGGGGCTCAGCGCCGACATCGTGGAGGTGGTTCGAGGCAGCCAC
ATTGTCCGGTCCACGGGTCGAAAAGACCGGCATAGCAAGGTCTGCACTGCAAAAGGC
CCAAGAGACCGCCGCGTCCGGCTCGCTGCTCACACCGCCATTCAATTCTACGACGTCC
AGGACCGCCTCGGCTACGACCGGCCCAGCAAGGCCGTCGATTGGCTCATCAAGAAGG
CCAAAGCCGCCATAGACGAGCTCGACGAGCTTCCCTCATGGAACCCACATTCAGTTTC
GACCACAACAGCGTCTACGGCGGTTACAGCCATGGAGGCCCAGAACCCAACAACCAC
TGGCTTCCACTGCTTTGCGGCGGTGGACGCAATTGGTTCTGCTAATCGAAGAGCAACA
ATGGTGGGGAGTGGAGTTTCAGAGCAAATTGTTCAAAATCAAAACCCACTTACCAACT
CGACTTTACTCCCACCATCGCTGGACTCTGACGCCATTGCAGACACTATCAAGTCCTTC
TTCCCAATGGGTGCTTCTGCAGGTGCAGCCGAGGCTCCATCGTCGACGATACAGTTTC
AGAACTACCCACCAGATTTACTGTCTAGAACAAGTAGCCAGAGCCAAGATCTACGCCT
TTCTCTGCATTCTTTCCAAGACCCAATTCTTCTGCAGCATCAACAAGCTCAAGCTCAGC
ATCACCAGGCTCAAACCCATCAAAATGAGCAAACCCTCTTCTCGGGAACACAGCAAC
AAAACCCACTTGGGTTTGATGGGTGGACAGAGCATCATCACCAGCAGCAGCAAGCAG

AGATGAACAGATTTCAGAGAATGGTAGCTTGGAATAGTGCTGGTGGTGGAGATACTGG
TAATGGTGGTGGATCGTCATCATCAGCTGGGTTTGTATTCAACTCGCTGCTCCCAACCC
AACAGAGTACTAGTTCTCTGCAGCCATCGCTGTTTGGCCAAGGCCACTTCTTTTCTTCT
CAGAGGGGACCCCTTCAGTCCAGTAACTCGCCTTCAGTTCGTGCTTGGATGATGGACC
AACAAAACCAACAATCGATTTCTCATGACCATCATCATCATCATCAAATCTCGCCAACC
ATTCATCATCATAATCAGTCTTCGTCTTCCATTTCCAACATGGGATTCGCCTCAGGAGGC
GGATTCCCCGGCTTCCACATCCCGGCACGAATTCACGGCGAGGAGGAACACGACGGC
ATCTCCGACAAGCCGTCCTCTGCTTCCTCCAATTCTCGCCATTGA
>PmTCP16
ATGATTAAGAGTCACAATGAAGCAGATTTACAAGAAGCAGCAGGCAATTCAAGCCGTG
ATGATCAAGCCAATAAATTCTCAACAAAAGCTAATGATTTGTCGCGTCCATCGACACCA
TGGCTACGGTTGAAGGATCCAAGGATTGTGCGTGTGTCCAGAGCTTTTGGAGGAAAGG
ACAGGCACAGCAAGGTTTGCACCGTAAAAGGGCTTCGAGATCGGCGGGTGAGGCTTT
CTGTACCCACTGCTATACAGTTGTATGACCTTCAAGAAAGGCTTGGTCTTAACCAGCCT
AGCAAAGTTGTCGATTGGTTGCTTGACGCCGCGAAGCATGAAATTGATGAACTTCCTC
CGCTGCCGCTGCCACCATCTGGGAATTTTGGCCTAAATCACCCATCATTAGTCCTCACT
TCATCTCATGGTGTCCAAACCCATGCTCATGCCCAATTATCTCATGATAATGGAGAAGGT
CCTAGTGGTGGAATTGCACCGGCCAGATCACATTTTTGGAGCACAAATTCGGATGCTAT
TTGGAGAGGGAAATCAAAAGAAATTGCAAGAGACACAACAAATGAGGAGGAAGAAG
AAAATCAGAAAGATATAAGCACTGGATCAGACCAAAAGGAAGAAGGAACTGTTGATG
GTAATTCATCATCAAACAACTTCTTAACCAGAATTAGCACCAACCATCCATTCTTTCCAG
GTCTTGTCAATAATGCCATGCCTTATGCTTACCATAATTGGGATCATAATCAGCCTTCAA
ATTTCCCACTATCTCAATTAGGAAGCCATGGATTCCCATCCCAAACTGCAGATCTCCAC
AACTTCATTAACGTCGTCTCGTTGCCCTCCACATTGTCTTTATCTACAACACAATCTCAT
TTCCCTTCACATAATGCTGCGGCTGCAGCAGAGATCGATCCGAGACAATTCAACCACAT
GCATATGTTAAGCTCAAGCAGTACTTCTCAGAACCTCTTGCCAAATTCTCTTTCACCAA
CTCTATACCCTAATAGCCAGACCCTGAGAGCACCCCATTTGAGCATGATGACTAAGCTT
GTGCGTTCTTCAAACAACACTACTGGAAGTGATCATCATCAGCCAAATACAGACCAGG
AGTCCCCTTCTAGATGA
>PmTCP17
ATGATATCAAATTCAAGGGAAAAGGGTTTCCAAGCAAAGCAAGAGGGCCACAACAAC
ACCAATAATGATGGAAACAGTAGTAATTTCAACAAGGAATCATCATCAAGTACTACTAC
TTCAAGACAATGGTCTGGATTTAGAAATCCAAGGATTGTACGTGTCTCGCGCACCTTCG
GAGGAAAAGACAGGCACAGCAAGGTTTCCACGGTGAGGGGATTGAGGGACAGGAGA
ATTAGGCTCTCAGTACCAACAGCCATTCAATTATATGACCTTCAAGACAGGCTTGGACT
TAGCCAGCCTAGTAAGGTAATAGACTGGCTGCTTGATGTTACTGAAGATGATATTGATA
AGCTCCCACCTCTCCAAGTTCCTCATGGATTTGCTCATCAATTTCATCAGCAAATGCTAA
ATCCTCATCATTCTCATGATCAAGGTCACCAGTCTAACAATTCTCTTGCTGCTGCTCCTT
TCTTTGATGTAAATTCTTCATTTATGGAGGCAGATCATCAAGCTCATCAAGTAGTTCATG
ACCATCAAAGAAGTTCTACTAGTACTAATGTGGGTGATCGGAAAGGCAAGTCAATCAA
AACACACGATGAACAAGATGATGATGATCAAAATCATCATCAAGATGGCAATATTGGGG
GTGGACAACTCTTGGCTCAGAAGCTATTTCCCCAAGGCAATCATCCTTCCTCCATACCT
GGCCTGCTAAACAATGCCATGGCATACAATTACTATCATAATTATTCAGAGCCTTCAAGT
TTATCTCTATCTCAATTTGGTGGCCATGGATTTCCACCAGTGCCCCAAATAGATCATCAT

AGCCATATGATGAGCAATGCCTTATCATTTTCAACTTCAATGCCATCTGGATCTCAATTG
TTCTTCTGTCCATCAACAGCAACACCCTCCCTTTTCGGTCCATATCCTCCGTATATTACC
AACCCGGTAGTGGAGAGAGGTACTAATACTAGTGAGCCAAGATCACAAGCCAACCATT
TCCAATTTTTGAGCTCATCAAATTCACCAAATCTCCTACCAAATGCTCTCATGTCTTCTC
TTCAGTCTTTGAAATCCTATCCAACATCGGTCAATCCCAAGCAGCTCCATTCGAATTCA
CAGGATAACAATGGAAGCCAACCAAACAAGGACCATAATTAA
>PmTCP18
ATGGAAGGAGGTGATCAAGATCATCAACTCCACCACCACCACCACCATCACTACCAAC
AACAGCAACATCACCACCGTCCAAACTTCCCCTTCCAGCTGCTCGAAAAGAAGGAGT
TAGATCAAGAAGCCGCCTCCTGCTCTAACTCGACCTCCCCCTACCCTTCTCTAGCCATC
ACCACTGTGGACCCTGCGACCGCCATCACCACCACCACCACAACAACCTCCACTCTCC
AAGCTTCCGCCGAGCCCTCCAAAAAGCCTCCTCCCAAGCGGACCTCCACCAAGGACC
GCCACACGAAAGTCGACGGACGCGGCCGTCGCATTCGAATGCCGGCCCTCTGCGCTG
CAAGAGTCTTCCAGCTCACCCGCGAGCTCGGCCACAACTCCGGCTCGAGCATGTCGGT
GCCGTCGCAGTTAAGATCTTCTTATTACAGCCCCAATTTCTCGGTTCACCAGAACCAGC
GCCGGAGCCTCTTTCAGGGCATCGGCCTCTCGTCCTCGGACAGCTCGTCGACTCTGCT
AAACTTTCAAACCAACAGCATGCACGCCTCCATGTTGCAGGCCAAGCAGGAGCTGCG
CGACACGGTGTCGTTGGATCTCTCGGAGACGGCGTCGGGGGAGGGGAGCATGGGAGG
GAGGAAGCGGAGGCCGCCGGAGCAGGACTTGAATCAAATGGGTGGGGGCGGCGGTG
GAGGTGGTGGAGGATACTTGTTGCAGTCTAGCACCGGCGCAGTCCCGGCCAGCCACC
ACCACAGCCAGATTCCGGCAAATTTTTGGATGGTGGCGAATTCTAATAACCAGGTTATG
AGTGGAGACCCTATTTGGACTTTCCCAGCTTCGGTCAACAACAGCGGGTTGTATAGAG
GGACCATGCCAGGTGGGTTACATTTCATGAATTTTCCTGCTCCTATGACCCTATTGCCTA
GTCAGCAACAATTGGGAGGCTCTGGCGGAGGCGGTGGAGGTAACGACGGCGATAACA
TGAGTGACGGGCAGTTAAATATGCTCGCTGGGCTTAACCCATACCGGCACATGTCCAGT
ACGGGTGTTTCAGAGTCCCAGCAAGCAAGTGGGTCCCATTCGCACCACGGGGGAGAC
GATCGGCATGATAGTACTAGTCACCATTCATAG
>PmTCP19
ATGGAGATCAATCAAACCTTACCCACCAACACACTCGCCATCACAGACCCACCAGAGA
ACCCTTCTTTACAAACTCAACCGCCACAACAAGAAGAGCAGCAACAGCTAGAACGGC
GTCGTTACATTGACAAGCATTCCACAGTGAACGGCCGTCACAGGCGGGTCAGAATACC
CGTGACCTGTTGCCCCGGTATTTTCCGGCTGACCCAAGAACTGGGTCACCGATCCGAC
GGCGACACCATCCAGTGGCTCCTCTCCCAGGTCCGACCCGAGCTCGTTCTCCCACCAC
AACCTAACAACAGGACCCGTCGGCTTCCACCCGACCCGGTTCCCCAACCCAATTGCAG
ATACCCGGGTGCAGCAGAAGATTGGTTGGATCACAAGGCAGTGGCTCGTCTTCCGAGC
GTCACTGTGAGGGCTACTGTGGTTCAAGCATCCACTGTGTTCTTCGACACTCCAGCGA
CTCTAGATAAAGCAGAGAGACTTGTTGCTGGTGCAGCTGCATATGGATCGCAATTGGTT
GTGTTTCCTGAAGCATTTGTTGGTGGTTATCCCCGTGGTTTGATGTTTGATTCTGCAACG
GCAACTCTTTCGCCGGAGGAGAAGCAGGCCTTCGAGAAGTACTATGCCTCAGCCATTG
ATGTGCCTGGTCCTGAAGTGGACAGGCTAGCAAAAATTGCAAGTAAATATAAAGTTCA
CTTAGTAATGGGAGTAGTGGAGCGAGTTGGATTCTATCTCTGTAGCACAGTGTTATTTTT
CGATTCATTTGGCCAGTGTCTTGGAAAACATCCCAAGCTACTACCACTGGCGTCAGAAT
CTCCAGTATGGTGTTCTGGACCAAAATTGCCAGTGTCTGTGTATGACACTGAAATTGGG
AGAATAGGTGGCCTTGTTTGTTGGGACAACAGAATGCCAGATCTAAGAACTCAATTATA

TGGCAAAGGCATTGAAATATATTGTGCACCCACAGCTGAAGCAAGGGAGATTTGGCGC
TCATCCATGACCCATATTGCCCTAGAAGGTGGCTGCTTTGTTCTATCTGCAAACCAGTTC
TGCAGACGAAAAGACTATCCACTGCCACTGGAATGTGTTTCTGGAGATTCAAATGATG
CGACATCCTTGGATATCATATGTGCTGGTGGGAGTGTTATTGTTTCCCCATCAGGAACCA
TATTGGCTGGACCTAATTACCAAGGAGAATCCCTTATCTCAGCTGATCTAGATCTTGTAG
AGATTGCTCGAGCAAAACTAGAATTTGGCGGAGTTGGGCTTGGGCACAACGCAGGGC
CAAATGCTGTTGGTTGGAGGAGGACAAGCATACCGAATCCCGATTTGTTTGCTGCAAC
CGTGAAAACAGAAGTTTCTGATCATGCAAATGTGCTGTATGCATGA

Supplementary Data 2. The genomic sequences of 19 *P.mume TCP* genes.

>PmTCP01
ATGTTTCCTTCTAGCAGCAGCGCTAGTAATTACCAGCTGCCTTTTCCATGTGATACCAAC
CAGCAGCCGCAGCTGCTCGAAAAGTCCTCCACAAATATCATTGATCAACATGGTCATG
AAAACCCTAACTCAATATCACATGATGACCATCATATTCGTCAGTACTATTCACACTACT
CTGAAGATCATCAGCAGCAGGCACCTAATAATTTTCTTGAACACGACGGGCTGCTTTTG
AGCTACTTGCTATCTCAGCAGCAGCTCTTGGTTGGCAGCTCCAGTCCCAATATGAACAG
TGCTACTTCTCATCATGTTCAAGTTCATGATACTACTGAAATCAGCGTTGTGGCTTCCAA
TTCAAACAAAAAAGTAATGGATCGTGTTGACGAGGGTCGGACTGCCCCAGCTGCAACT
TCGAAAGGGTGCTCTTCAAAGAAGAAGAGTAATACATCAAATGGAGAAAGCAAGAAC
CCGAAGGCTCCTCGAAAGAGATCAAGCGGGAATAAAGACAGGCACAGCAAGATCTAC
ACAGCTCAAGGCCCAAGAGACAGAAGAATGAGGCTGTCTCTTCAAATTGCCCGCAAG
TTCTTTGATCTTCAAGACATGCTGGGGTTTGATAAAGCCAGCAAAACCATCGAGTGGCT
ATTCACAAAGTCCAAGACTTCCATCAAGGAACTCAAGCAGCACCTCAACATCTCACCC
CTGGCAGCTTACAGCTGTAGCACCAATGCCAACGCTACTTCTACCAGCACAATCACTG
CAAAGATGAATTCGTCCACTTCTGAGAATAGCGAAGTTGCATCCAAGATTCAGGAGAC
TGCAAATGGCGACGTACACTCGATTGGGACATTGGAGAGAGATAAAAAGAACAGGAA
GTTATGTGTGGTTGCGAGAGAATCGAGGGTCGAGGCAAGAGCAAGGGCAAGGGAGAG
AACAAGGGAGAAGATGATGAGGACCAGATCAGGTTTGGATCAATATCAGAATTTAAGA
AAACAAAGCCCTGATCATGATCAAGTACCACAAAACCCTAATAATGATCAATTCTCGGG
CGGTCACTACTTATGGTCCTCTAATTGTCCTAATGATCAATTGTTTGAACCTGGAATGAT
GATGAATTCCATGGTGATGAGCATCAATCATCATGATGAGAAGGTTGTGGGCAGCACA
ACAATTCCCAGTGGAGCAAATTCCGAGGATTATGATTTTCCAAGTTTTCCTGGGAATTG
GGGCCAAATCAACAATTCCAAGAACATTACAGGTAATAACGTGCTGCAAGTAGATCCA
AACCCTAGTAACTCATCAACTATGGCTACTGCTCCTCCTACCTCATACCCCCTAGAACA
AAAGCCTAGTTCAGTTTTCATGTCCACCTCATCAAGCATGCACGAACAAAACCCTAGTT
CAATCTTCGTCACAACCTTGATTGCTGAAGACCAAAATCCCACTACTTCTTCAAATTTT
GGGACCAATTCAAGTATTGTTTTGAAGTCCCATTTCCTTGGAAATTAA

>PmTCP02
ATGTTTTCATCTTGCAGTAGCAATATTAATAGTAATTCTGTAAGTCCTTTTCAACCTTACT
TTCCCTTGTCTTCTTCAAATTACCACCCTCCTCCTCCTCCCCCTCCTCCTCCTTTTCCTTG

TGTGAACCAAGAGGCTTGTAGTGGAGACATTTTCCTTCACCACAATATTCAAGGGTACC
CCATCTCTGGTCAATTTCCACTTCATAACAATGCCTTAATGGCACCTCCTCTTCCTCAAA
GCTTGACCCATTTGGGAGTTTCATCAAACACTGTACCTCCTAGCATAAACGCTGATGAT
CATCATCACTACCATAATTACTATGGTGCCATTAACAATGATAATATTTTTCCTCATTTCCT
TCATTCTTCTAGAGAAGATATAGTAGCGCCGCCGATGAAGAAAGATCGGCACAGCAAG
ATCTTCACTGCTCAGGGCTTGAGGGACCGGAGGGTTAGACTGTCCATCAATGTTGCCC
GTCAATTCTTTGACCTCCAAGACCTGCTAGGGTTTGACAAGGCAAGTAAAACCCTAGA
ATGGCTTCTCACCAAGTCAAGAAGGGCCATCAAACAGCTTGGGACTCGGAACAAACA
CTTGACCTGCAGTACTAGTACTGGGCGTTCCAAGAGTTTGACTTCGAGTTCAGAGTGT
GATGACGATGACGTCGATTCAGACACCAACGAGGTGGAAAACGTAGCAAGCAAAGAA
AAAGAGGTGATGTTGATGATGAAGAAAAAGATGAAAGAATCTGAATCTGCTAATGTTT
ATGGCACGAAAGACTCGAGGGCAAGGGCAAGAGCAAGGGCAAGGGAAAGGACAAG
AGAGAAATTAATGTGCACTGGGAGCAGGCCTCAGATGTTGAACCAATTGAAGTTATTC
AATGAGCTTGATCACCATCAATCTAACAATAATTGTAAAACAATGTCATCATCTTCAGCA
AAGGCCAATATTGGAGATCATCCTGAGAATCAGGAACTGGGCTCCCTCCTAGCTAATCA
ATTAGCTCATCATCATGAGGACGATCCGTCTGTTCATGTCATCAAAAGAAACAAGTTGA
AGCAGTATTCATCAGTTTATTCCAATTATAATCAGCAAAAGTATCTCAATGTGGTGTCAG
CAGAAAATACAGATCAGAGTTCCAACAGCCACATCCAATTTCCCAATGCATTTCAAAAT
TGGGACACCAACGGCGCCTTCCCATGCCCTAACATTCATTGTGCCATTACCAGCATCAA
TCTTTCTACCGGTATATATCTTTATATTGTCCAAAAGTATAAACCCTTTTGCTCTTTTTGTT
CCTGATTTTAGATTTAATAATCATGATCTCAAATCTGTAGGAAATTAA
>PmTCP03
ATGGGCCTAGAGAGCCTACAAGAGTGGGTTAGAAGCAAAGCCATTCATGAGATGCCAG
GTGTCGGAATCTCAAGCATCCAGCTTGGCAGTTGAGTCCCTTACAAGAGAAGAGATGG
CATCATATATTGGTTGCTGCTGCAATTATTATAGGAATGCAATGCAGTGCAGTGCAGGAG
GAGAAATTATAATAATGAAGGGAGAGGAAAGGAAGGGAGAGGGGAATCCATGGATGG
ATGGATGGATGGATGGACCATGGCGTGGTCCATATGCTATGACTCCATGGAATGTGGGA
AATGGGCTTTTGATGTGTCTGATGATGATCTGATCTGACTGATGGGCAGATGAGATGAG
ATGAGGTGGTGAAAATATGAAATTGGCAGTTAAAGTTGCTTTCATATCTGCGTAGTCAA
AACTAGCTACAGTGTCAAAAGGAGTAAGAGAAAAAGTAGGAAAGAAAGGACAGTTT
GGAGAGAGAGAGAGAGAGAGAGAGAGAGAGGAGACTCAAACAATGACTCACAGGAGG
AGGGAGTAAAAAAAAACCAAACCCAGTCTACCTAGAGTTAAATTTTGACGATGGGGTA
CATTTGGATTTTAATATTTTTATGGTTTTGTCTTCTCATTCTTTGCCAAAAACAGTGAAA
ACTCATGAAAGTCTAGGACTTTTCAGGTCACCTTCTTCAATCAAAGAGGACCCCTTTTT
CTTTTTGCCTTATTTTTTTTTCCCTGTTCATGGATCCCAAGGGCTCAAAGCAGACACAA
GAGATACCCAGCTTCTTGAGCCTCCCACAGCCTCAGCAACAGCAGCAACAACAACAG
CAGCAGCAGCAGCAGCAGCAGCCCAACATGAGTGAGAACAAACCTGCTGAAATCAAA
GATTTCCAGATTGTGATTGCAGACAAAGAAGAGGGGAAGAAGCAGTTGGCGCCCAAG

AGAAGCTCAAACAAAGACAGACACACGAAGGTTGAAGGCAGGGGAAGGAGGATACG
GATGCCTGCTCTGTGTGCAGCAAGAATTTTTCAATTGACCAGAGAATTGGGCCACAAA
TCTGATGGGGAAACCATACAGTGGCTTTTGCAGCAGGCCGAACCGTCGATAATTGCAG
CCACCGGGACCGGTACGATACCAGCATCGGCTTTAACAGCGGCAGGAGGCTCTGTTTC
ACAGCAGGGGACTTCTCTATCAGCTGGATTGCACCAAAAGATTGATGAATTGGGGGG
TCCAGTATTGGGTCAGGGAGTAGGACCAGTTGGGCAATGGTAGGTGGGAATTTGGGGA
GACCCCATGTGGCCACTGGGCTATGGCCCCCTGTCAGTAGCTTTGGTTTCCAGTCATCA
TCTGGTCCATCAACAACAAATCTGGGCAGTGAGAGTTCAAATTACATGCAAAAGATTG
GCTTTCCTGGCTTTGACTTGCCTGTCTCCAACATGGGTCCTATGAGTTTCACCTCAATTT
TGGGTGGTGGGAGTAACCAACAGCTTCCTGGCTTGGAGCTTGGGTTGTCTCAGGATGG
TCATATTGGGGTTTTGAACTCACAAGCCTTGAGCCAGATTTACCAGCAGATGGGGCATG
CTAGAGTACACCAGCACCAGCACCAGCACCAGCACCAGCAACCTCCTGCTAAGGATG
ACTCTCAAGGCTCAGGACAGTAG
>PmTCP04
ATGGAAGCTGAAAATGGGATCCGCTGCAGGCCCAATTTTCCACTACAATTTCTAGACA
AAAACCAAGATGATGACATCCAAGAAGCGCCAGGAGACCCGGGTTCCAACCAGAACC
GACCCGGCTCCTCCCTGACCCAAGAGCAGCAACCCAACAACGGCAAGAAGCCTCCTC
CGAAGAGGACCTCCACCAAGGACAGGCACACAAAAGTGGACGGGCGAGGCCGGCGG
ATCCGTATGCCGGCCACGTGTGCCGCTAGGGTTTTCCAGCTCACGAGGGAGCTAGGGC
ACAAGTCGGACGGTGAGACCATTGAGTGGCTTCTCCAGCAGGCCGAACCCGCCGTCA
TCGCAGCCACCGGGACCGGCACCATTCCCGCAAACTTCACCTCCCTCAACATCTCGTT
GCGGAGCTCCGGCTCTAGCATGTCGGCGCCGTCGCACTACTTGAGGCCAAACAGCAGT
AATTATTTCATGAACTTCAATCCCAACAACTTGGGTGCAGCAGCTGCGTCGCAGCTGAT
TATGGACGAGAACTCGCAGCGCGGCAGGATTTTGTTCCCCGGCGTTGGGTTGTTGTCG
TCTGAGAGCTCCCCGTCGTCGTCTATGTTGTTGAATTTCAACAATGCCATGTTGGAAGC
CAAGCGCGAGTTACGTGATCAGGGCGGTTCTCTCGGTGAGGTGTCCGAGGGCGGCGA
GGCCAGCACCATGGGAAGGAAGCGAATTAGGCCGGAGCAAGATTTGTTATCGTCTTCG
TCGTCCTCGTCCTCGTCATCACAAAGTAGTCATCATCAGATGGGGATATCGAACAGCTA
CATGTTGCAATCAGCCAGCACGGGGTCAATTCCGGCGAGTCAAAGTACAATTCCGGCG
ACATTTTGGATGGTGGCAAATAACCCTAGTAGCGGCAGCGGAGGCGGCGGTCATCATG
TCCATGAGCCTCCCATTAATCCCATGTGGACAATTCCTAGTATTAATAATGGTATTAACA
GCAATATGTATAGGGGGGCTTCGGTGTCTTCTGGGGGTGGGCATGGTCTGCATTTTATG
AATTTTGCTCCACCTCCAATGGCGGCCATATTACCTAGCCACATGGGATCAACTCCTCA
CGGCGGTGGAGGCTCAATTGCCGACAGCCACTTGGGTGTGATGGCTGCGCTCAACGC
GTACAGGCCGATTCTCGGTGGGGTCATGGCGGAGCCACCGGGTGGGAATAATGGACAG
CATTCACACCATGGAGCAGACGAGGGACATGATCGTGACAGCAATGCTAGGTAG
>PmTCP05
ATGGGCTCCATCTCAATTCAAGCTGGCCTCACCTCTACTCATACACCGACAACAACTTC

TGCTACTGTTTCTGCACAACCACCAGCTCCTTCCTCCTCTTCTTTGTCTTCTTCCTCCTC
CTCCTCTCCCTCCTCCACCTCCACCTCCACCGCTCCTCCTCCCCACTTGGTCGACGCCT
CCCTCGCCATCGCCACCAGATCCGACTCCCACCCCTCAACCCGACCAGCCCCGCTCCT
CGACTCCGCCAAGAAAAACCAAATTCATCAGCAGCAATTGACAATTTCACCCACCAGT
ACTACTACTGCGACACCACCAGCACCACCAGCCGCAGCAGCAGCAACCCAAGTAGTG
AAACGATCCACCAAGGATCGGCACACGAAAGTCGAAGGCCGAGGCCGCCGCATACGA
ATGCCGGCCACCTGCGCCGCCAGAGTCTTCCAGTTGACGAGAGAATTGAGCCACAAG
TCCGACGGCGAGACAATCGAGTGGCTCCTCCAGCAAGCCGAGCCTGCGATCATCGCCG
CCACCGGAACGGGGACCATCCCGGCCAATTTCTCCAGTTTGAACATATCCCTGCGTAGC
AGCGGATCAACGCTCTCCGCTCCGCCTTCCAAGTCCGCCTCTCATTCCTTTCCCGGCGC
CTTGGCTCTTGCCCATCACCCTCACTACGACGAGGGTTTTCCCCATTCGGGTTTGTTGG
GGTTTCATCAACAGCAGCATCATCACCATCATCATCAGCAGCATCAGTCTCCTCTTATGA
CCGCGGATCAAATTGCAGAAGCGCTTCCAAGCGGCGGCGGAGACAGCGGCGGTGGAG
ACTCTACCGACAGTTATATGAGAAAGCGCTTCAGAGAAGATCTGTTCAAGGATGATAAT
CAAGGGCGAGGCGAAGGCGGCGGAGGCAGCAGCGGAGGAGGAGGGTCGCCGTCTGG
GAATAAGGCTTTTAAGAGCAGCGGAGGCTTACAATTGCAAAAACAACAACAGGGGTC
GGAGGGGGAGGCAGGACCTTCAGGGCTGCTGAGGCCAACTTCGAATATCCTGCCACC
CACTGCCATGTGGGCAGTGGCACCCGCCCCGAACAGCGGTGCTGCTGGCAGCACATTC
TGGATGCTGCCAGTAACCGCTGGTGCTGGTGGGCCCTCAATGCCCACCGGGACGTCCG
GCGCTGGTCCTTCCGAGGCTGCACAGATGTGGACGTTTCCGTCCGCACCTGCCAGTCA
CGGGAGTACATTGCAAGCGCCATTGCATTTCATGCCGAGGTTTAACCTTCCAAGCAGTC
TTGAATTCCAGCAAGCCGGCAGCCGAGGGAGCCCTCTCCAATTGGGTTCCATGCTAAT
GCAGCAACAGCCATCACAGCATCTTGGGCTTGGAGTCGCCGACTCGAATTTAGGCATG
TTGGCTGCTCTGAATGCTTCTTACTCTAGAGGTGGTTTGAATATGAATTCTGAGCACCA
CCAGCAGAATCATCCATTGGATCATCATCATCAACAACAACAACACCAGCCTCAAACTA
CTGATAGTGGAGATGAGGCCCCAAATAGTTCTCAATGA
>PmTCP06
ATGGCAGAGAACAAGCCTGCGCAAATCAAGGACTTGCAGATTCTGAAAGCAAACAAA
GATGATGAAAACAAGAAGCACCTGGCGCCCAAGAGGAGCTCCAACAAGGACAGGCA
CAAAAAGGTGGATGGTAGAGGGAGAAGAATACGGATGCCTGCCTTGTGTGCAGCTAG
GGTTTTTCAATTGACCAGAGAATTGGGTCACAAGTCTGATGGGGAAACAATTCAGTGG
CTGCTGCAGCAAGCTGAGCCATCAATTATAGCTGCCACTGGCTCTGGAACTGTACCTGC
TTCTGCTCTTGCAGCCGCAGGGTCCTCTGTTTCTGAACAGGGGAGCTCTGTTTCCTCTG
TTTCTTCTGGGTTGCATACAAGGATGGAAGGACTAATAAGACCAAGTGTTGGGTCTGA
AGGCAGTGCCAACTGGGCATTTCATATGGGCAGATCTAATGTAGCAAGTGGGGTTTGG
CCCTTGCCCTTTCTCAGTGGGGTTAGGTCTGGCTTTGATCAAAATTCTGGTCAGGCAAC
AGTCAATTTTGGGAGTGAAAACTTGAACATCATGCAGCATAAGTTTGGGTTTCATGGGT
TTGATGTTCCTGGTATGAATTTGGGGTCAATGGGTTTTCCCACAATTCTAAGTGGAAGC

AACCAGCAAGTTCCAGGTTTAGAGCTTGGGCTCTCACAGGATGGTCATATTGGGGTGG
TGAGTTCTCAAGCTTTGACCCAATTTCACCAGCACAAAGGGCAGAGGCCAGGTGGTG
TAGCTTCCTTCAATCAGCAGCAGCAGCATCAGCAGCCATCTGATACGAATGAATGA
>PmTCP07
ATGTTTCCCTCAAACTCAAACAATAATCTCAACAATACTGGCAACGAATTACCCGTGTC
TTATCCTCATGTCGACCAATCCTTTTTCCACAGCCGGCCTTTTCTCCATGAAATCACTAC
ATTAAACCCCAATTCCCTTCATCCCAATCTCAATTCCAAACAAGAAGAAGAACAACGA
CAACAAGAAGGCCCTCATCACCATCCTTTGTCTTTCTTCTACTTCCCCTCTCCTTTAGAA
GACGACGATGTTTTACTCTTCCAACAACACCATCACTATGACCATCAGCATGACCATGT
CCATGACATGCCGCTCCATGACTCACAACAAGCACCACCCCTTACTACTATGAGGGAA
GCTGTTGCTGCTAACAACACCTTAGCCGATGATCATCGTCAGACCACCACCACTTCCAC
TACTCTCAATATTAAAATGGTGGATTGGGATTCCAACAAAAATCATGGCGAGATGATGA
ATATGGATCAGCCACAGATCCCGAGAAGAAGGTCTTGCAAGAGAGACCGCCACAGCA
AGATAAATACTGCCCGAGGCCTCAGGGACCGCAGAATGAGATTGTCGCTTGAAGTTGC
CCGCAAGTTCTTCTGGTTGCAAGACGCACTGCACTTTGACAAGGCCAGCAAAACCGTT
GAGTGGTTGCTCATTCAGGCCACACCTGAAATCAAGAAACTAGTAGGAGACTGCAAA
CACATGATGAGCTCTACCAAAAGCACATCTCCTGCCACTTCTGAGTCATGCGAAGTGAT
ATCTGGCATAGACGAGGCTGCAACAAACACCAACATTCACATTAATATTGATGGGGGA
AATGATGGTGATGATAAACTAATACAAAGCTGCGAGATCCAGCCTTCAGCCAAAGAGA
GAAAGGTTGCCCGCCGGCAATTGTCAAGGAAGACCGCATTTCACCCTCTTTCAAAAGC
TTCGAGGGAAAAGGCAAGGGCAAGGGCAAGGGAAAAAGCAAGAGAAAAGCAGCGG
ACGCATCAAAGAGTAGTTGATGTTGATGATCAATCTAAGAAGCAAAGAGGTGATCAGG
AAAACCTGAGCCGATTGGGTTCTTGGAGCCCCTTTGAAACTGGGGAAGAATCAGCTG
GGACTCAAAGCCACAACAACAACAACATAAGCATCAATTCATTGGAGGGGCTGGTTCA
TCACGAGATTGAAGAACCAATGAGCAGCTGCCAAGTAGGAGATCATCCAGACTTGGTT
GTTGATCATGGAACTACACATGATCCTTTGGTGATTATGGGAAAGTGGAGCCCCCCTTC
AATATTCAGTTCTCTGCAGCAAAACATTGGAATTTCCCAAGAGGTAACTTCCAACAAC
AAGTTTTGA
>PmTCP08
ATGACCATGACTGAGAAACGAGAACTTGAACGAGACCAAGCCACAACGTCCGTCGAT
CTCAGAATCAACGGCGGAGAAGAATCCGACTCAGAAGAGCCCGCCGGCCCATCCCAA
GCTCTTATGCTCGCGCCCAAGGACGAGCGGGACGTAGCTATGCCTGTGGCGGTGCACG
CGCCGAAGAGAGCGTCGACGAAGGACCGGCACACGAAAGTGGAGGGACGGGGCCGG
AGGATCCGAATGCCCGCCACGTGCGCGGCTCGGATCTTCCAGCTGACCCGGGAGCTAG
GGCACAAGTCCGACGGCGAGACCATCCGGTGGCTCTTGGAGCACGCCGAGCCGGCCA
TCATCGCCGCCACCGGCACCGGAACCGTGCCTGCGATCGCGATGTCCGTGAATGGGGC
GCTCAAAATCCCCACGTCCGCTCCAGACCCGAGACCCGGCGAGGACCCGCCCGACAA
GAAAAAGCGCAAGCGGAACTCAAACAGCGAGTACGTGGACTTGAACGACGGCGTTTC

GGCCTCGGCGGGTTTAGCCCCTTTAACAACGGAACGACATCACCAACAGCCACCGAC
AGCAGCGGTGCAAGCGGTGGTACCTCAAGGCGTGCTCCCCATGTGGGCCATACCGTCA
AACGGCGTCGTTCCCGGAGCTTTCTTCATGGTCCCTTCCGCTTCGACCCAGCCTCACAT
ATTTACATTTCCAACCACCGTTGCCGCCGCTCCGTTTATCAACATTTCGGCCCGACCCAT
ATCCTCTTTCGTGGGGCCCAGTTCGGCTTCTGCAACGACCCATATGGCCGCTTCCACCG
CCCCTCAGACGCTCAGAGACTTCTCTTTGGAAATTTACGACAAGAAGGAGCTTCAATT
CATGTCTGGCTCTTCAAACCATTGA
>PmTCP09
ATGGAGGTGGAGGAGATTCAAGCCCAAGCCTGTAAGTTCCCAAGAATTGGAAATGGC
AGTAGCAGAGCCACTAACCCAGCTGCCGACGATGAAGATCAAGACCCATCGTGCCTAG
ACTTCAAGAGGGATACCACTGCGGACGCCGGCAACCGCCTCCGCGGGTGGCACCACT
CCCGCATCATCCGCGTCTCGAGGGCCTCCGGCGGCAAAGATCGGCACAGCAAGGTGT
GGACTTCAAAGGGACTCAGAGACCGGAGGGTACGGTTGTCTGTAACCACAGCCATTC
AGTTCTATGATCTTCAAGATCGGCTGGGTTACGACCAACCCAGCAAAGCAGTGGAGTG
GCTGATCAAAGCTGCTGCTGAGGCCATAGCTGAGCTTCCATCTCTCAACAATTCCTTCC
CTGACACTCCCAAGCAGCTCAGTGATGAGAAGAGGGCGAGTTGTGAGCATGGGTTTG
ATTCAGCTGAGGTTGAGCTCGAGGGGCACGGCCATGGCGACCCCAATTATCATCACCA
TCAGAACCAGAACCAGACTCAGAACCAAAGCCAGTACCTTTCTCTGTCCAAATCTGCT
TGTTCTAGTAATTCCGAGACCAGCAAGGGATCCGGGCTGTCTCTCTCCCGGTCCGAGAT
TCGGGTGAACCGGGTCAAGGCCCGGGAGCGTGCCCGGGAGAGAGCAGCCAAGGATA
AGGAGAAGGAGAGCAACGAGTCAGCTTCACACATTGCTCACCACCCCCAGCAGAACA
GCAGCAACTTGAACAACTCCATTTCTCAAAGCGCTTCGTTCACCGAGCTTCTCACTGG
TGGCATCGGTACCAACAGCAGCAACAACAACAATAGTCCAACCGCAGCTGCTCATCAG
CAGCAGAACCACGGCGGCGGCGGCGGCGAGCCCATTTTGTTCCACAAGGCAGCAGCG
GCAGGAGGAGGGGGACCGATGGATTACTTCAGTTCCGGACTTCTCGGGCTGTCCTCGT
CGACCCGAACCCACCACTCGTCCGGGTTCCCGGGTCAAATCCAGTTGGGGATGAACTC
CATTCCGCAGACAATGTCGGTTGTGTCCCCGTTCAGTGTGTCTGGGGATCATCATCACA
ACCACAACCCGGAGCTTCAGCATTTCTCGTTTGTTCCCGACCACCTCATCCCCGTTACG
ACGTCGTCGCAGCCGGGCAATGGCGGCGACTACAATCTGAATTTTTCGATCTCTTCTTC
CGGCGGCCTTGCTGGTTTCAATAGGGGGACCCTTCAGTCCAATTCTTCCTCATCACCGT
CTCTTTTGCCTCACCACCTTCAGAGGTTTTCTCCCATAGACGGATCATCCAATGTACCCT
TCTTCATCGGAGCCGCGGCGGCTCCAACCATGGAGAACCACCATCACCACCACCACCA
CCATCAGCAACACCAACAGCAGTTCCCAGCTGGGTTTGATCGCCGCTTGCAACATCCC
TACGGCGACGGAAGCCGGCATTCAGACCACAAGGGAAAAGCAAAGAACTGA
>PmTCP10
ATGACGTCGTATTTGGAGGATCAAGACGACGACGGAGGCACCTCCGATCTCAGCACCA
GCACCGGCGACCCCGAAGACAACAACAACAACGGAAACGGGGTCGTTTCGACGCAA
CCCAACTTCGACGAGACGACGGCGTTTCAGCAGCTGAAGGAGGAGCCCATTGACTCC

GACCCTCCGCCGCAGGCTCACTCCATCGGCATGGTTCCGGTGGCGATGCAAATGCCCA
TGTCCGTGGCTGTACCCGTTTCCAACCCGACCCGACGGGCCTCTACCAAGGACCGCCA
CACGAAAGTCGAGGGACGCGGGCGGAGGATCCGAATGCCCGCCACGTGCGCGGCCCG
GATCTTCCAGCTAACCCGGGAGCTGGGCCACAAGTCCGACGGTGAAACCGTCCGGTG
GCTCCTTGAACACGCCGAGGAAGCCATCATCGAGGCCACCGGCACCGGCACCGTTCC
CGCCATCGCCGTCTCCGTCGGTGGGACCCNNNNNNNNNNNNNNNNNNNNNNNNNNNNNNN
NNNNNNNNNNNNNNNNNNNNNNNNNNNNNNNNNNNNNNNNNNNNNNNNNNNNNNNNNNNN
NNNNNNNNNNNNNNNNNNNNNNNNNNNNNNNNNNNNNNNNNACGCCGTTTCGCAGTCGTC
GGGTCTGGCTCCGGTGGGGCCCGCGGCGCCACAAGGCCTGGTTCCGGTCTGGGCCGT
GGGCGGCGCCGGGCTGATGGTCCCAGCGAATGCTTTCTGGATGGGCCCGGTGGGCTCG
GGTGGGGGACCGTCCGGCCCACAACCGCAGATATGGGCCCTGTCGCCGACGGTGACG
CCGGTTTTCAACGTGGCTGGGGCTACCCGGCCCGTATCGAGTTTTGTGGCGAATAACG
GAGGAGGAGTGGAGGTTCGGGCTCCGTCGCCGGCACTGTCAAACTCAGCGGTGAGTA
CGAGCACGGTGGGGCCGAGGGCGGCGAAGAGGTCGTCGACGACAATGGCGCCGAGC
GTGAGCTCGTCGAGTAACAACAGTAACGGTAGCGGGAGTGGCGCGAGCAAGGCTCAG
ATGCTGAGAGATTTTTCTCTTGAAATTTACGACAAGCAGGAGTTGCAGTTCATGGGTCG
ACCCGTCGGCTCGCCAACAACTCATCAACATCAAACCCAGTGA
>PmTCP11
ATGGGAATGAAGAGCACTGGAGGAGGAGGAGGAGGAGAGATTATACAAGTTCAAGGA
GGCCACATTGTTCGATCCACCGGCCGCAAAGACCGCCACAGCAAGGTCTACACCGCCA
AAGGCCCCCGAGACCGCCGCGTCCGGTTGTCTGCCCACACCGCCATCCAATTCTACGA
TGTTCAAGACCGCCTCGGCTATGACCGCCCCAGCAAAGCGGTTGACTGGCTCATCAAG
AAAGCAAAATCCTCCATTGACAAGCTTGCTGAGCTTCCTCCTTGGCACCCTATCACTGG
TGTTGCAGCAAACAATGCTGAGCCCGATCAATCCAATCCAAATGAAATGGTGATTGCA
GGAGGAGCAGAGGAAACGGAGTCCTCTGGCTACAATTTCCACCAGCTGCAGAGGCAA
ATGGGTGAGAATAACAACAACCAAGCAAATAATGTTTCAAGCTTCAACATTCCTCCATC
TCTAGACTCAGACACCATAGCTGACACCATGAAATCATTCTTCCCCACAAGCTCAGCA
GCCAATTCATCCATCAATTTCCAAAGCTACCCAGCGGATCATGATCTGATTTCAAGAAC
CACCAACCTAAACCCTTGCCAAGACCTTGGCCTCAGCAATCCCAAGGGGACACCAAC
CACAACAACGATCAGATTCAGACCCTTTTCGCCGCGGGGGCGGGAGGATCAACAGTG
GGATTTGACACTAGTTATCAGAGAATGGTGGCATGGAGCAATCAGAACAGAGGAGGCG
ATGGTGGATTTGTATTCAACTCACACTCACAAGCATTGCCACAGCAGGCATATGCTCAT
GCTCAGGGGGGTACCCTTCAGTCCAGTTTTTCTCCATCAGTTTCAGCTCGGGCCTGGA
ACGATTCTTCTATCTTTGGCACACAGCAGAGAACACAGCAGCAGCAGCAGCCAATGAT
TCACCATTCTTCAATCTTTGGCACCCGCTTCGCGTCTGATGGCGGCTTGCCAGTGTTCT
GCATCCCCACGCGTATTGACGCTGAGGAGGCCGATAATGGCGGTGTTTCAGATCGACC
ATCATCCACTTCCTCTCCCAATTCCACCCACCATTGA
>PmTCP12

ATGGAACCGAATCAAAGGCAGAGCCTTGAAGAAAGCAACGAGCTTGAACAGCAGAG
CAATAGCAGTAGCAACGACAACACCACCTCAGCATCCGACCCATCAGTGGCGGACCCT
CCGGAGAAAATATTTCCCTTGACGGCGCCGACGATGAAAGAAGAGCTGACGGACACA
GTGCAAGAACTGGACGAAGGATCTCTGCCTATGGGTCTTATTCAGGTGCCGGTTCCTAC
GTCGTCGGAGAAACAAGTGGTGGCGGCAAAGAGGTCGTCGAAGGACCGCCACACGA
AGGTGGAGGGCCGGGGCCGGAGGATCCGAATGCCCGCCACGTGTGCGGCCAGGATTT
TTCAACTGACCCGAGAACTTGGCCACAAGTCCGACGGCGAAACCATAAGGTGGCTGC
TCGAGCAGGCCGAGCCTGCTATTATGCAGGCCACGGGCACAGGCACAATCCCCGCCAT
TGCCGTCTCCGTCGGTGGCACTCTAAAGATACCCACAACGTCACCGGCGAGACCCAAC
GGTGAGATTACTGAAATACCCAGAAAGCGACGGAAGAGAGGGTCGAACAGTGAGTTC
GTTGATGTGCATGAGCAGAGCTCTGTGTCTAGTGGGTTGGCCCCAATGAGCTATGGCG
GCGGTGGCGGTGGCGGTGGGGCACATGGGTTGGTGCCCATGTGGCAAGTTGGGGCAA
CTGGTGCAGCTGGGCCTTTCTTCATGTTCCCAAACAACGGGGCTGTGAACCCAAACCA
GCCCCAGCTTTGGGCTGTTCCAGCTGCGGATGCTGCAACCCCGATTTTCAATTTCCAAG
CAAGACCCATCTCCAATTTCTTGTCGGCTTTTCAACCAGGTGTTCACGTTGTTGGTGGG
GATGTGCAGTTGCAGGCCTCTTCGGGTTCGATTTCGAGCGGTGCAACTTCGGGTTCGG
GGGGGAGCTGTTCGTCTAGTTTGGGACCCAGTTTGGGCTCCGCCTCAGGAACCAGAGC
TAATAAGAACACCATTTCTACTGGTAGTGGGACTGGTGCTAGTGCTGCTGCTGCTGCTG
CTAGTGCCACAACAACTCAGATGTTGAGGGATTTTTCTTTGGAGATTTATGATAAGAGA
GAGCTTCAGTTCATGGGTGCAAATTCACAAACACCATACTCTAAGCCTTAG
>PmTCP13
ATGAGTCACCTGCAAGACATTCTGCGTCTGCAAATGTTACAACAGAGAGGATCAAAGA
ATGAAGATCAAGATCAACCAGAAGATGTTCAAGAAGAAGAACAACAATCACAGAAGA
GACTAATTGGTCAATATCAGCATGTTCAAGAACCACCAAACTATGGACCATTAAATGGC
AAGATGTTGAATACCCACATAGCAAAATCCTCCAGGAAAAGTTGTTACTCAATGTCTTC
TTCTTCTTCACATTTGGCATCTGAACAAGCGAAAATCAACAATGCGAGATATGGGAAG
ATTGTAAAAGTTCATGGAGGCCACATTGTCCGATCCACTGCAAGAAAAGAAAGGCACA
GCAAGGTGTACACTTCCAAAGGTCCTAGAGACCGGAGGTTCCGGCTGTCAGCTCCGA
CTGCTATACAGTTCTATGATGTCCAAGACCGCCTTGGCTACGACCGGCCAAGTAAGGCC
ATTGATTGGCTCATTGAGAAGGCCAAGGCAGCAATTGAGGCTCTCTCTGAGTCTGAAC
TACCTGGAAAAGAGTATGACTGTACTAATATCAACAACTCTGCTCAACAGACAGAGCA
AGACATTGGAGAAGAGAGTATGCGTCAGTTTCAGCATCACCAGAGAAGCTATGGTGGT
GAACCAGAAAAACTGAACAATGTGAACAGTTTTAAAGAACCAGTTCTTGATCACTACC
AGTTAAGTTCAATGAACTATGCTGAAGAAGCCCTCAATTCTGGCTCAAGTTTAACAGAT
TCCAAAATGGAGGTGGCTTGGTTTCAAAGTTTGTTGGCTTGGAATTATAATGCAGGTGA
TGGAGGAGAAGGCTGTCCTTTCAATTCATCCCATGTATATCTGCAATAG
>PmTCP14
ATGGGCTCAGAGATGGCCCTTGCCCCTCTACGCAACCACGAAGACCCGACCCTCATCC

CCACTGCCGTTTTGTCCTCCGCCGCCCTAGAAACGGCGTCGTCCCGTCAGCACTCCCA
GCAATCCATACAATCTCTACAGCCCAAGACACATAACCAGCTCTCTCAGACCCGAAAG
TCCGCGTCTTCCAGGGACCGCCACACCAAAGTCAACGGCCGGGGACGGCGAGTCCGG
ATGCCGGCCATGTGCGCCGCCCGGATCTTCCAGCTGACCCGGGAGCTCGGCCACCGCT
CCGACGGGGAGACCATCGAGTGGCTCCTCCGCCACGCCGAGTCCTCCATCGTCGCCGC
CACCGGTACCGGCACTCTCCCGGCCGAGCCGATTTCGACCTCCGCCCCCGCCGTCTCC
TCCCAAGTGCCCTCCCTCGCTTGCCGGGCCCACCCGCTCAGCTCCCTCAATGGCGGCG
GGCAGTTCATGTACCCGCTGGTGTCGTCGGCTCATCAGGCTCAACCGAATCAGCCGCA
TCAGCAGCCGAGCATTCGGCTGGACTTGTGCCAGCCGGCGGGGTTGGACTACACCGA
GTACCGGCAGCACATGCCGTTCACGTCGTTGCTGCTGCATCCGGCGGAGAATGAGGAG
GAGGAGGAGGAGGATGGGCAGCAGGAGGAGGCTCTGTGTCTTGGGGATGGATAG
>PmTCP15
ATGGGCGACACCCACCACCACCACCACCCCCAAGCAACGACGTCGTCCAGACTGGGG
ATAAGGCCGTCTTCGGGGCTCAGCGCCGACATCGTGGAGGTGGTTCGAGGCAGCCAC
ATTGTCCGGTCCACGGGTCGAAAAGACCGGCATAGCAAGGTCTGCACTGCAAAAGGC
CCAAGAGACCGCCGCGTCCGGCTCGCTGCTCACACCGCCATTCAATTCTACGACGTCC
AGGACCGCCTCGGCTACGACCGGCCCAGCAAGGCCGTCGATTGGCTCATCAAGAAGG
CCAAAGCCGCCATAGACGAGCTCGACGAGCTTCCCTCATGGAACCCACATTCAGTTTC
GACCACAACAGCGTCTACGGCGGTTACAGCCATGGAGGCCCAGAACCCAACAACCAC
TGGCTTCCACTGCTTTGCGGCGGTGGACGCAATTGGTTCTGCTAATCGAAGAGCAACA
ATGGTGGGGAGTGGAGTTTCAGAGCAAATTGTTCAAAATCAAAACCCACTTACCAACT
CGACTTTACTCCCACCATCGCTGGACTCTGACGCCATTGCAGACACTATCAAGTCCTTC
TTCCCAATGGGTGCTTCTGCAGGTGCAGCCGAGGCTCCATCGTCGACGATACAGTTTC
AGAACTACCCACCAGATTTACTGTCTAGAACAAGTAGCCAGAGCCAAGATCTACGCCT
TTCTCTGCATTCTTTCCAAGACCCAATTCTTCTGCAGCATCAACAAGCTCAAGCTCAGC
ATCACCAGGCTCAAACCCATCAAAATGAGCAAACCCTCTTCTCGGGAACACAGCAAC
AAAACCCACTTGGGTTTGATGGGTGGACAGAGCATCATCACCAGCAGCAGCAAGCAG
AGATGAACAGATTTCAGAGAATGGTAGCTTGGAATAGTGCTGGTGGTGGAGATACTGG
TAATGGTGGTGGATCGTCATCATCAGCTGGGTTTGTATTCAACTCGCTGCTCCCAACCC
AACAGAGTACTAGTTCTCTGCAGCCATCGCTGTTTGGCCAAGGCCACTTCTTTTCTTCT
CAGAGGGGACCCCTTCAGTCCAGTAACTCGCCTTCAGTTCGTGCTTGGATGATGGACC
AACAAAACCAACAATCGATTTCTCATGACCATCATCATCATCATCAAATCTCGCCAACC
ATTCATCATCATAATCAGTCTTCGTCTTCCATTTCCAACATGGGATTCGCCTCAGGAGGC
GGATTCCCCGGCTTCCACATCCCGGCACGAATTCACGGCGAGGAGGAACACGACGGC
ATCTCCGACAAGCCGTCCTCTGCTTCCTCCAATTCTCGCCATTGA
>PmTCP16
ATGATTAAGAGTCACAATGAAGCAGATTTACAAGAAGCAGCAGGCAATTCAAGCCGTG
ATGATCAAGCCAATAAATTCTCAACAAAAGCTAATGATTTGTCGCGTCCATCGACACCA

TGGCTACGGTTGAAGGATCCAAGGATTGTGCGTGTGTCCAGAGCTTTTGGAGGAAAGG
ACAGGCACAGCAAGGTTTGCACCGTAAAAGGGCTTCGAGATCGGCGGGTGAGGCTTT
CTGTACCCACTGCTATACAGTTGTATGACCTTCAAGAAAGGCTTGGTCTTAACCAGCCT
AGCAAAGTTGTCGATTGGTTGCTTGACGCCGCGAAGCATGAAATTGATGAACTTCCTC
CGCTGCCGCTGCCACCATCTGGGAATTTTGGCCTAAATCACCCATCATTAGTCCTCACT
TCATCTCATGGTGTCCAAACCCATGCTCATGCCCAATTATCTCATGATAATGGAGAAGGT
CCTAGTGGTGGAATTGCACCGGCCAGATCACATTTTTGGAGCACAAATTCGGATGCTAT
TTGGAGAGGGAAATCAAAAGAAATTGCAAGAGACACAACAAATGAGGAGGAAGAAG
AAAATCAGAAAGATATAAGCACTGGATCAGACCAAAAGGAAGAAGGAACTGTTGATG
GTAATTCATCATCAAACAACTTCTTAACCAGAATTAGCACCAACCATCCATTCTTTCCAG
GTCTTGTCAATAATGCCATGCCTTATGCTTACCATAATTGGGATCATAATCAGCCTTCAA
ATTTCCCACTATCTCAATTAGGAAGCCATGGATTCCCATCCCAAACTGCAGATCTCCAC
AACTTCATTAACGTCGTCTCGTTGCCCTCCACATTGTCTTTATCTACAACACAATCTCAT
TTCCCTTCACATAATGCTGCGGCTGCAGCAGAGATCGATCCGAGACAATTCAACCACAT
GCATATGTTAAGCTCAAGCAGTACTTCTCAGAACCTCTTGCCAAATTCTCTTTCACCAA
CTCTATACCCTAATAGCCAGACCCTGAGAGCACCCCATTTGAGCATGATGACTAAGCTT
GTGCGTTCTTCAAACAACACTACTGGAAGTGATCATCATCAGCCAAATACAGACCAGG
AGTCCCCTTCTAGATGA

>PmTCP17

ATGATATCAAATTCAAGGGAAAAGGGTTTCCAAGCAAAGCAAGAGGGCCACAACAAC
ACCAATAATGATGGAAACAGTAGTAATTTCAACAAGGAATCATCATCAAGTACTACTAC
TTCAAGACAATGGTCTGGATTTAGAAATCCAAGGATTGTACGTGTCTCGCGCACCTTCG
GAGGAAAAGACAGGCACAGCAAGGTTTCCACGGTGAGGGGATTGAGGGACAGGAGA
ATTAGGCTCTCAGTACCAACAGCCATTCAATTATATGACCTTCAAGACAGGCTTGGACT
TAGCCAGCCTAGTAAGGTAATAGACTGGCTGCTTGATGTTACTGAAGATGATATTGATA
AGCTCCCACCTCTCCAAGTTCCTCATGGATTTGCTCATCAATTTCATCAGCAAATGCTAA
ATCCTCATCATTCTCATGATCAAGGTCACCAGTCTAACAATTCTCTTGCTGCTGCTCCTT
TCTTTGATGTAAATTCTTCATTTATGGAGGCAGATCATCAAGCTCATCAAGTAGTTCATG
ACCATCAAAGAAGTTCTACTAGTACTAATGTGGGTGATCGGAAAGGCAAGTCAATCAA
AACACACGATGAACAAGATGATGATGATCAAAATCATCATCAAGATGGCAATATTGGGG
GTGGACAACTCTTGGCTCAGAAGCTATTTCCCCAAGGCAATCATCCTTCCTCCATACCT
GGCCTGCTAAACAATGCCATGGCATACAATTACTATCATAATTATTCAGAGCCTTCAAGT
TTATCTCTATCTCAATTTGGTGGCCATGGATTTCCACCAGTGCCCCAAATAGATCATCAT
AGCCATATGATGAGCAATGCCTTATCATTTTCAACTTCAATGCCATCTGGATCTCAATTG
TTCTTCTGTCCATCAACAGCAACACCCTCCCTTTTCGGTCCATATCCTCCGTATATTACC
AACCCGGTAGTGGAGAGAGGTACTAATACTAGTGAGCCAAGATCACAAGCCAACCATT
TCCAATTTTTGAGCTCATCAAATTCACCAAATCTCCTACCAAATGCTCTCATGTCTTCTC
TTCAGTCTTTGAAATCCTATCCAACATCGGTCAATCCCAAGCAGCTCCATTCGAATTCA

CAGGATAACAATGGAAGCCAACCAAACAAGGACCATAATTAA

>PmTCP18

ATGGAAGGAGGTGATCAAGATCATCAACTCCACCACCACCACCACCATCACTACCAAC
AACAGCAACATCACCACCGTCCAAACTTCCCCTTCCAGCTGCTCGAAAAGAAGGAGT
TAGATCAAGAAGCCGCCTCCTGCTCTAACTCGACCTCCCCCTACCCTTCTCTAGCCATC
ACCACTGTGGACCCTGCGACCGCCATCACCACCACCACCACAACAACCTCCACTCTCC
AAGCTTCCGCCGAGCCCTCCAAAAAGCCTCCTCCCAAGCGGACCTCCACCAAGGACC
GCCACACGAAAGTCGACGGACGCGGCCGTCGCATTCGAATGCCGGCCCTCTGCGCTG
CAAGAGTCTTCCAGCTCACCCGCGAGCTCGGCCACAAGTCTGACGGAGAAACCATCG
AGTGGCTTCTCCAGCAGGCCGANNNNNNNNNNNNNNNNNNNNNNNNNNNNNNNNN
NNNNNNNNNNNNNNNNNNNNNNNNNNNNNNNNNNNNNNNNNNNNNNNNNNNNNN
NNNNNNNNNNNNNNNNNNNNNNNNNNNNNNNNNNNNNNNNNNNNNNNNNNNNNN
NNNNNNNNNNNNNNNNNNNNNNNNNNNNNNNNNNNNNNNNNNNNNNNNNNNNNN
NNNNNNNNNNNNNNNNNNNNNNNNNNNNNNNNNNNNNNNNNNNNNNNNNNNNNN
NNNNNNNNNNNNNNNNNNNNNNNNNNNNNNNNNNNNNNNNNNNNNNNNNNNNNN
NNNNNNNNNNNNNNNNNNNNNNNNNNNNNNNNNNNNNNNNNNNNNNNNNNNNNN
NNNNNNNNNNNNNNNNNNNNNNNNNNNNNNNNNNNNNNNNNNNNNNNNNNNNNN
NNNNNNNNNNNNNNNNNNNNNNNNNNNNNNNNNNNNNNNNNNNNNNNNNNNNNN
NNNNNNNNNNNNNNNNNNNNNNNNNNNNNNNNNNNNNNNNNNNNNNNNNNNNNN
NNNNNNNNNNNNNNNNNNNNNNNNNNNNNNNNNNNNNNNNNNNNNNNNNNNNNN
NNNNNNNNNNNNNNNNNNNNNNNNNNNNNNNNNNNNNNNNNNNNNNNNNNNNNN
NNNNNNNNNNNNNNNNNNNNNNNNNNNNNNNNNNNNNNNNNNNNNNNNNNNNNN
NNNNNNNNNNNNNNNNNNNNNNNNNNNNNNNNNNNNNNNNNNNNNNNNNNNNNN
NNNNNNNNNNNNNNNNNNNNNNNNNNNNNNNNNNNNNNNNNNNNNNNNNNNNNN
NNNNNNNNNNNNNNNNNNNNNNNNNNNNNNNNNNNNNNNNNNNNNNNNNNNNNN
NNNNNNNNNNNNNNNNNNNNNNNNNNNNNNNNNNNNNNNNNNNNNNNNNNNNNN
NNNNNNNNNNNNNNNNNNNNNNNNNNNNNNNNNNNNNNNNNNNNNNNNNNNNNN
NNNNNNNNNNNNNNNNNNNNNNNNNNNNNNNNNNNNNNNNNNNNNNNNNNNNNN
NNNNNNNNNNNNNNNNNNNNNNNNNNNNNNNNNNNNNNNNNNNNNNNNNNNNNN
NNNNNNNNNNNNNNNNNNNNNNNNNNNNNNNNNNNNNNNNNNNNNNNNNNNNNN
NNNNNNNNNNNNNNNNNNNNNNNNNNNNNNNNNNNNNNNNNNNNNNNNNNNNNN
NNNNNNNNNNNNNNNNNNNNNNNNNNNNNNNNNNNNNNNNNNNNNNNNNNNNNN
NNNNNNNNNNNNNNNNNNNNNNNNNNNNNNNNNNNNNNNNNNNNNNNNNNNNNN
NNNNNNNNNNNNNNNNNNNNNNNNNNNNNNNNNNNNNNNNNNNNNNNNNNNNNN
NNNNNNNNNNNNNNNNNNNNNNNNNNNNNNNNNNNNNNNNNNNNNNNNNNNNNN
NNNNNNNNNNNNNNNNNNNNNNNNNNNNNNNNNNNNNNNNNNNNNNNNNNNNNN
NNNNNNNNNNNNNNNNNNNNNNNNNNNNNNNNGGGCGCGCCCGGTCCACCGGTGA
CCGCCGCACGGGGCCCCGGCACAACCCCCGCCAATTTCACATCGCTCAACATCTCGCT

CCGCAGCTCCGGCTCGAGCATGTCGGTGCCGTCGCAGTTAAGATCTTCTTATTACAGCC
CCAATTTCTCGGTTCACCAGAACCAGCGCCGGAGCCTCTTTCAGGGCATCGGCCTCTC
GTCCTCGGACAGCTCGTCGACTCTGCTAAACTTTCAAACCAACAGCATGCACGCCTCC
ATGTTGCAGGCCAAGCAGGAGCTGCGCGACACGGTGTCGTTGGATCTCTCGGAGACG
GCGTCGGGGGAGGGGAGCATGGGAGGGAGGAAGCGGAGGCCGCCGGAGCAGGACTT
GAATCAAATGGGTGGGGGCGGCGGTGGAGGTGGTGGAGGATACTTGTTGCAGTCTAG
CACCGGCGCAGTCCCGGCCAGCCACCACCACAGCCAGATTCCGGCAAATTTTTGGATG
GTGGCGAATTCTAATAACCAGGTTATGAGTGGAGACCCTATTTGGACTTTCCCAGCTTC
GGTCAACAACAGCGGGTTGTATAGAGGGACCATGCCAGGTGGGTTACATTTCATGAAT
TTTCCTGCTCCTATGACCCTATTGCCTAGTCAGCAACAATTGGGAGGCTCTGGCGGAGG
CGGTGGAGGTAACGACGGCGATAACATGAGTGACGGGCAGTTAAATATGCTCGCTGGG
CTTAACCCATACCGGCACATGTCCAGTACGGGTGTTTCAGAGTCCCAGCAAGCAAGTG
GGTCCCATTCGCACCACGGGGGAGACGATCGGCATGATAGTACTAGTCACCATTCATAG
>PmTCP19
ATGGAGATCAATCAAACCTTACCCACCAACACACTCGCCATCACAGACCCACCAGAGA
ACCCTTCTTTACAAACTCAACCGCCACAACAAGAAGAGCAGCAACAGCTAGAACGGC
GTCGTTACATTGACAAGCATTCCACAGTGAACGGCCGTCACAGGCGGGTCAGAATACC
CGTGACCTGTTGCCCCGGTATTTTCCGGCTGACCCAAGAACTGGGTCACCGATCCGAC
GGCGACACCATCCAGTGGCTCCTCTCCCAGGTCCGACCCGAGCTCGTTCTCCCACCAC
AACCTAACAACAGGACCCGTCGGCTTCCACCCGACCCGGTTCCCCAACCCAATTGCAG
ATACCCGGGTGCAGCAGAAGATTGGTTGGATCACAAGGCAGTGGCTCGTCTTCCGAGC
GTCACTGTGAGGGCTACTGTGGTTCAAGCATCCACTGTGTTCTTCGACACTCCAGCGA
CTCTAGGTAGCCACTTTGCCCAACTCTCTCAAATCGGTTGCTTTTGTTTTCATATTTGGG
GTCTTGTTGAGGTTTCTGGGGTTTATGGGATGCTTAAGATCTTTGTTTTCTTTGTATTCTA
TGATTTGGTTAAGTGGGACCAGGATTTGTGGGTCAAAATATGTTCTTTCTGTATGAAGA
TTGGGAGATTAAGGGCCCACTACCCTCCTCCTTCCTTAAGATTGTGGACCTGAGGTTTT
GTTATGTACTCTGGGACCATGTCCTTGTAGGCCATTGCAAGTTCATATTATATTTTTTGAT
CCCCAAATATTTTAATTTCCTAACCATTTACCATTTGGGTTTCTGTCATTTGTGGGTTGTT
GGCAGATAAAGCAGAGAGACTTGTTGCTGGTGCAGCTGCATATGGATCGCAATTGGTT
GTGTTTCCTGAAGCATTTGTTGGTGGTTATCCCCGTGGTTTGATGTTTGATTCTGCAACG
GCAACTCTTTCGCCGGAGGAGAAGCAGGCCTTCGAGAAGTACTATGCCTCAGCCATTG
ATGTGCCTGGTCAGATTCCTTCCACTGAAGATGAAGTTTTCGTTTGAATGTGGTCTTCT
GCATAATATTACCTTCTTGCTTGTTCCGAGTGTTAGCATGTGTGACATTTAGAATTGTTG
ATAATCTTTCCCTCTATGTTTAGAAGCACTCTTAACAATTTCAAGCTGGTTTATATCAAG
TCTCTGGCAGTCAAAAATTTTGGTATATACTTTGTAAGAAGTTAAGAACTATTGTAAATC
TTTTCTCAGATGCACATAGCTGTTTGTACCTTTATATGTGGTTGTCTTGACCCCCGGCTA
AAGTTGGAAGCTTGTGTAAGTTTAGCTTTCTGATAAACTATTGATTTGTTTTATCTAGTC
AATGTCTTTGAATTTTCAGTTTTATGATAAATCATTGATTTACTTTATCTGTGGCTGTTGA

AATTCTATGTCATACATATAATACATTCTATCTTTTTATTTGAATAATGAAATTATCTTTG
GTGTTTGATATGCAGGTCCTGAAGTGGACAGGCTAGCAAAAATTGCAAGTAAATATAA
AGTTCACTTAGTAATGGGAGTAGTGGAGCGAGTTGGATTCTATCTCTGTAGCACAGTGT
TATTTTTCGATTCATTTGGCCAGTGTCTTGGAAAACATCCCAAGCTACTACCACTGGCG
TCAGAATCTCCAGTATGGTGTTCTGGACCAAAATTGCCAGTGTCTGTGTATGACACTGA
AATTGGGAGAATAGGTGGCCTTGTTTGTTGGGACAACAGAATGCCAGATCTAAGAACT
CAATTATATGGCAAAGGTAAATATGCTATCATACATAAACACTCTTGCTTAGTGTTTTCA
ACTTTTCAACCAAAATCACCATGACCATCCAACATAAGAAAAGGTTCCACTTATCTGCC
GCAGCCATGGTCATGATTTCAAGTCCACTAGTTACTTTATCACCTTTAGAATACTACGAT
TGTTTTCTTTAACATGGGACTCACTGGAATTTTAGTCCAAGAGTTCTTGGTTTCTGATCT
TAGTTTCTTGCCCCTATATCATTAGGCATTGAAATATATTGTGCACCCACAGCTGAAGCA
AGGGAGATTTGGCGCTCATCCATGACCCATATTGCCCTAGAAGGTGGCTGCTTTGTTCT
ATCTGCAAACCAGTTCTGCAGACGAAAAGACTATCCACTGCCACTGGAATGTGTTTCT
GGAGATTCAAATGATGCGACATCCTTGGATATCATATGTGCTGGTGGGAGTGTTATTGTT
TCCCCATCAGGAACCATATTGGCTGGACCTAATTACCAAGGAGAATCCCTTATCTCAGC
TGATCTAGGTATGAACAATTTGTTTCAGAAACATGTAAATCACAGTTAGGATGATATGCA
AACAGCCATCTCTCTTCCTCCCTCACACACAGAGTTGAGATGATATCTAAACTACTATTT
GATGTTCTTAGATCTTGTAGAGATTGCTCGAGCAAAACTAGAATTTGGCGGAGTTGGGC
TTGGGCACAACGCAGGGCCAAATGCTGTTGGTTGGAGGAGGACAAGCATACCGAATC
CCGATTTGTTTGCTGCAACCGTGAAAACAGAAGTTTCTGATCATGCAAATGTGCTGTAT
GCATGA

Supplementary Data 3. The protein sequences of 19 *P. mume TCP* genes, 24 *A. thaliana TCP* genes and other 5 *TCP* genes.

> PmTCP01
MFPSSSSASNYQLPFPCDTNQQPQLLEKSSTNIIDQHGHENPNSISHDDHHIRQYYSHYSED
HQQQAPNNFLEHDGLLLSYLLSQQQLLVGSSSPNMNSATSHHVQVHDTTEISVVASNSNK
KVMDRVDEGRTAPAATSKGCSSKKKSNTSNGESKNPKAPRKRSSGNKDRHSKIYTAQGPR
DRRMRLSLQIARKFFDLQDMLGFDKASKTIEWLFTKSKTSIKELKQHLNISPLAAYSCSTN
ANATSTSTITAKMNSSTSENSEVASKIQETANGDVHSIGTLERDKKNRKLCVVARESRVEA
RARARERTREKMMRTRSGLDQYQNLRKQSPDHDQVPQNPNNDQFSGGHYLWSSNCPND
QLFEPGMMMNSMVMSINHHDEKVVGSTTIPSGANSEDYDFPSFPGNWGQINNSKNITGN
NVLQVDPNPSNSSTMATAPPTSYPLEQKPSSVFMSTSSSMHEQNPSSIFVTTLIAEDQNPTT
SSNFGTNSSIVLKSHFLGN*

> PmTCP02
MFSSCSSNINSNSVSPFQPYFPLSSSNYHPPPPPPPPPFPCVNQEACSGDIFLHHNIQGYPISG
QFPLHNNALMAPPLPQSLTHLGVSSNTVPPSINADDHHHYHNYYGAINNDNIFPHFLHSSR
EDIVAPPMKKDRHSKIFTAQGLRDRRVRLSINVARQFFDLQDLLGFDKASKTLEWLLTKSR
RAIKQLGTRNKHLTCSTSTGRSKSLTSSSECDDDDVDSDTNEVENVASKEKEVMLMMKK
KMKESESANVYGTKDSRARARARARERTREKLMCTGSRPQMLNQLKLFNELDHHQSNN
NCKTMSSSSAKANIGDHPENQELGSLLANQLAHHHEDDPSVHVIKRNKLKQYSSVYSNY

NQQKYLNVVSAENTDQSSNSHIQFPNAFQNWDTNGAFPCPNIHCAITSINLSTGN*
> PmTCP03
MGLESLQEWVRSKAIHEMPGVGISSIQLGNKEEGKKQLAPKRSSNKDRHTKVEGRGRRIR
MPALCAARIFQLTRELGHKSDGETIQWLLQQAEPSIIAATGTGTIPASALTAAGGSVSQQGT
SLSAGLHQKIDELGGSSIGSGSRTSWAMVGGNLGRPHVATGLWPPVSSFGFQSSSGPSTTN
LGSESSNYMQKIGFPGFDLPVSNMGPMSFTSILGGGSNQQLPGLELGLSQDGHIGVLNSQA
LSQIYQQMGHARVHQHQHQHQHQQPPAKDDSQGSGQ*
> PmTCP04
MEAENGIRCRPNFPLQFLDKNQDDDIQEAPGDPGSNQNRPGSSLTQEQQPNNGKKPPPKR
TSTKDRHTKVDGRGRRIRMPATCAARVFQLTRELGHKSDGETIEWLLQQAEPAVIAATGTG
TIPANFTSLNISLRSSGSSMSAPSHYLRPNSSNYFMNFNPNNLGAAAASQLIMDENSQRGRI
LFPGVGLLSSESSPSSSMLLNFNNAMLEAKRELRDQGGSLGEVSEGGEASTMGRKRIRPE
QDLLSSSSSSSSSSQSSHHQMGISNSYMLQSASTGSIPASQSTIPATFWMVANNPSSGSGGG
GHHVHEPPINPMWTIPSINNGINSNMYRGASVSSGGGHGLHFMNFAPPPMAAILPSHMGS
TPHGGGGSIADSHLGVMAALNAYRPILGGVMAEPPGGNNGQHSHHGADEGHDRDSNAR
*
> PmTCP05
MGSISIQAGLTSTHTPTTTSATVSAQPPAPSSSSLSSSSSSSPSSTSTSTAPPPHLVDASLAIATR
SDSHPSTRPAPLLDSAKKNQIHQQQLTISPTSTTTATPPAPPAAAAATQVVKRSTKDRHTKV
EGRGRRIRMPATCAARVFQLTRELSHKSDGETIEWLLQQAEPAIIAATGTGTIPANFSSLNIS
LRSSGSTLSAPPSKSASHSFPGALALAHHPHYDEGFPHSGLLGFHQQHHHHHHQQHQSP
LMTADQIAEALPSGGGDSGGGDSTDSYMRKRFREDLFKDDNQGRGEGGGGSSGGGGSPS
GNKAFKSSGGLQLQKQQQGSEGEAGPSGLLRPTSNILPPTAMWAVAPAPNSGAAGSTFWM
LPVTAGAGGPSMPTGTSGAGPSEAAQMWTFPSAPASHGSTLQAPLHFMPRFNLPSSLEFQ
QAGSRGSPLQLGSMLMQQQPSQHLGLGVADSNLGMLAALNASYSRGGLNMNSEHHQQN
HPLDHHHQQQQHQPQTTDSGDEAPNSSQ*
> PmTCP06
MAENKPAQIKDLQILKANKDDENKKHLAPKRSSNKDRHKKVDGRGRRIRMPALCAARVF
QLTRELGHKSDGETIQWLLQQAEPSIIAATGSGTVPASALAAAGSSVSEQGSSVSSVSSGLH
TRMEGLIRPSVGSEGSANWAFHMGRSNVASGVWPLPFLSGVRSGFDQNSGQATVNFGSE
NLNIMQHKFGFHGFDVPGMNLGSMGFPTILSGSNQQVPGLELGLSQDGHIGVVSSQALTQ
FHQHKGQRPGGVASFNQQQQHQQPSDTNE*
> PmTCP07
MFPSNSNNNLNNTGNELPVSYPHVDQSFFHSRPFLHEITTLNPNSLHPNLNSKQEEEQRQQ
EGPHHHPLSFFYFPSPLEDDDVLLFQQHHHYDHQHDHVHDMPLHDSQQAPPLTTMREAV
AANNTLADDHRQTTTTSTTLNIKMVDWDSNKNHGEMMNMDQPQIPRRRSCKRDRHSKI
NTARGLRDRRMRLSLEVARKFFWLQDALHFDKASKTVEWLLIQATPEIKKLVGDCKHMM
SSTKSTSPATSESCEVISGIDEAATNTNIHINIDGGNDGDDKLIQSCEIQPSAKERKVARRQL
SRKTAFHPLSKASREKARARAREKAREKQRTHQRVVDVDDQSKKQRGDQENLSRLGSW
SPFETGEESAGTQSHNNNNISINSLEGLVHHEIEEPMSSCQVGDHPDLVVDHGTTHDPLVI
MGKWSPPSIFSSLQQNIGISQEVTSNNKF*
> PmTCP08
MTMTEKRELERDQATTSVDLRINGGEESDSEEPAGPSQALMLAPKDERDVAMPVAVHAPK
RASTKDRHTKVEGRGRRIRMPATCAARIFQLTRELGHKSDGETIRWLLEHAEPAIIAATGTG

TVPAIAMSVNGALKIPTSAPDPRPGEDPPDKKKRKRNSNSEYVDLNDGVSASAGLAPLTTE
RHHQQPPTAAVQAVVPQGVLPMWAIPSNGVVPGAFFMVPSASTQPHIFTFPTTVAAAPFINI
SARPISSFVGPSSASATTHMAASTAPQTLRDFSLEIYDKKELQFMSGSSNH*
> PmTCP09
MEVEEIQAQACKFPRIGNGSSRATNPAADDEDQDPSCLDFKRDTTADAGNRLRGWHHSRI
IRVSRASGGKDRHSKVWTSKGLRDRRVRLSVTTAIQFYDLQDRLGYDQPSKAVEWLIKAA
AEAIAELPSLNNSFPDTPKQLSDEKRASCEHGFDSAEVELEGHGHGDPNYHHHQNQNQT
QNQSQYLSLSKSACSSNSETSKGSGLSLSRSEIRVNRVKARERARERAAKDKEKESNESAS
HIAHHPQQNSSNLNNSISQSASFTELLTGGIGTNSSNNNNSPTAAAHQQQNHGGGGGEPIL
FHKAAAAGGGGPMDYFSSGLLGLSSSTRTHHSSGFPGQIQLGMNSIPQTMSVVSPFSVSG
DHHHNHNPELQHFSFVPDHLIPVTTSSQPGNGGDYNLNFSISSSGGLAGFNRGTLQSNSSS
SPSLLPHHLQRFSPIDGSSNVPFFIGAAAAPTMENHHHHHHHHQQHQQQFPAGFDRRLQH
PYGDGSRHSDHKGKAKN*
> PmTCP10
MTSYLEDQDDDGGTSDLSTSTGDPEDNNNNGNGVVSTQPNFDETTAFQQLKEEPIDSDPP
PQAHSIGMVPVAMQMPMSVAVPVSNPTRRASTKDRHTKVEGRGRRIRMPATCAARIFQLT
RELGHKSDGETVRWLLEHAEEAIIEATGTGTVPAIAVSVDAVSQSSGLAPVGPAAPQGLVP
VWAVGGAGLMVPANAFWMGPVGSGGGPSGPQPQIWALSPTVTPVFNVAGATRPVSSFVA
NNGGGVEVRAPSPALSNSAVSTSTVGPRAAKRSSTTMAPSVSSSSNNSNGSGSGASKAQM
LRDFSLEIYDKQELQFMGRPVGSPTTHQHQTQ*
> PmTCP11
MGMKSTGGGGGGEIIQVQGGHIVRSTGRKDRHSKVYTAKGPRDRRVRLSAHTAIQFYDV
QDRLGYDRPSKAVDWLIKKAKSSIDKLAELPPWHPITGVAANNAEPDQSNPNEMVIAGGA
EETESSGYNFHQLQRQMGENNNNQANNVSSFNIPPSLDSDTIADTMKSFFPTSSAANSSIN
FQSYPADHDLISRTTNLNPCQDLGLSNPKGTPTTTTIRFRPFSPRGREDQQWDLTLVIREW
WHGAIRTEEAMVDLYSTHTHKHCHSRHMLMLRGQRTQQQQQPMIHHSSIFGTRFASDGG
LPVFCIPTRIDAEEADNGGVSDRPSSTSSPNSTHH*
> PmTCP12
MEPNQRQSLEESNELEQQSNSSSNDNTTSASDPSVADPPEKIFPLTAPTMKEELTDTVQELD
EGSLPMGLIQVPVPTSSEKQVVAAKRSSKDRHTKVEGRGRRIRMPATCAARIFQLTRELGH
KSDGETIRWLLEQAEPAIMQATGTGTIPAIAVSVGGTLKIPTTSPARPNGEITEIPRKRRKRGS
NSEFVDVHEQSSVSSGLAPMSYGGGGGGGGAHGLVPMWQVGATGAAGPFFMFPNNGAV
NPNQPQLWAVPAADAATPIFNFQARPISNFLSAFQPGVHVVGGDVQLQASSGSISSGATSGS
GGSCSSSLGPSLGSASGTRANKNTISTGSGTGASAAAAAASATTTQMLRDFSLEIYDKREL
QFMGANSQTPYSKP*
> PmTCP13
MSHLQDILRLQMLQQRGSKNEDQDQPEDVQEEEQQSQKRLIGQYQHVQEPPNYGPLNGK
MLNTHIAKSSRKSCYSMSSSSSHLASEQAKINNARYGKIVKVHGGHIVRSTARKERHSKV
YTSKGPRDRRFRLSAPTAIQFYDVQDRLGYDRPSKAIDWLIEKAKAAIEALSESELPGKEY
DCTNINNSAQQTEQDIGEESMRQFQHHQRSYGGEPEKLNNVNSFKEPVLDHYQLSSMNY
AEEALNSGSSLTDSKMEVAWFQSLLAWNYNAGDGGEGCPFNSSHVYLQ*
> PmTCP14
MGSEMALAPLRNHEDPTLIPTAVLSSAALETASSRQHSQQSIQSLQPKTHNQLSQTRKSASS
RDRHTKVNGRGRRVRMPAMCAARIFQLTRELGHRSDGETIEWLLRHAESSIVAATGTGTL

PAEPISTSAPAVSSQVPSLACRAHPLSSLNGGGQFMYPLVSSAHQAQPNQPHQQPSIRLDLC
QPAGLDYTEYRQHMPFTSLLLHPAENEEEEEEDGQQEEALCLGDG*
> PmTCP15
MGDTHHHHHPQATTSSRLGIRPSSGLSADIVEVVRGSHIVRSTGRKDRHSKVCTAKGPRD
RRVRLAAHTAIQFYDVQDRLGYDRPSKAVDWLIKKAKAAIDELDELPSWNPHSVSTTTAS
TAVTAMEAQNPTTTGFHCFAAVDAIGSANRRATMVGSGVSEQIVQNQNPLTNSTLLPPSLD
SDAIADTIKSFFPMGASAGAAEAPSSTIQFQNYPPDLLSRTSSQSQDLRLSLHSFQDPILLQH
QQAQAQHHQAQTHQNEQTLFSGTQQQNPLGFDGWTEHHHQQQQAEMNRFQRMVAWN
SAGGGDTGNGGGSSSSAGFVFNSLLPTQQSTSSLQPSLFGQGHFFSSQRGPLQSSNSPSVR
AWMMDQQNQQSISHDHHHHHQISPTIHHHNQSSSSISNMGFASGGGFPGFHIPARIHGEEE
HDGISDKPSSASSNSRH*
> PmTCP16
MIKSHNEADLQEAAGNSSRDDQANKFSTKANDLSRPSTPWLRLKDPRIVRVSRAFGGKD
RHSKVCTVKGLRDRRVRLSVPTAIQLYDLQERLGLNQPSKVVDWLLDAAKHEIDELPPLP
LPPSGNFGLNHPSLVLTSSHGVQTHAHAQLSHDNGEGPSGGIAPARSHFWSTNSDAIWRG
KSKEIARDTTNEEEEENQKDISTGSDQKEEGTVDGNSSSNNFLTRISTNHPFFPGLVNNAM
PYAYHNWDHNQPSNFPLSQLGSHGFPSQTADLHNFINVVSLPSTLSLSTTQSHFPSHNAAA
AAEIDPRQFNHMHMLSSSSTSQNLLPNSLSPTLYPNSQTLRAPHLSMMTKLVRSSNNTTGS
DHHQPNTDQESPSR*
> PmTCP17
MISNSREKGFQAKQEGHNNTNNDGNSSNFNKESSSSTTTSRQWSGFRNPRIVRVSRTFGG
KDRHSKVSTVRGLRDRRIRLSVPTAIQLYDLQDRLGLSQPSKVIDWLLDVTEDDIDKLPPL
QVPHGFAHQFHQQMLNPHHSHDQGHQSNNSLAAAPFFDVNSSFMEADHQAHQVVHDH
QRSSTSTNVGDRKGKSIKTHDEQDDDDQNHHQDGNIGGGQLLAQKLFPQGNHPSSIPGLL
NNAMAYNYYHNYSEPSSLSLSQFGGHGFPPVPQIDHHSHMMSNALSFSTSMPSGSQLFFC
PSTATPSLFGPYPPYITNPVVERGTNTSEPRSQANHFQFLSSSNSPNLLPNALMSSLQSLKS
YPTSVNPKQLHSNSQDNNGSQPNKDHN*
> PmTCP18
MEGGDQDHQLHHHHHHHYQQQQHHHRPNFPFQLLEKKELDQEAASCSNSTSPYPSLAIT
TVDPATAITTTTTTTSTLQASAEPSKKPPPKRTSTKDRHTKVDGRGRRIRMPALCAARVFQL
TRELGHNSGSSMSVPSQLRSSYYSPNFSVHQNQRRSLFQGIGLSSSDSSSTLLNFQTNSMH
ASMLQAKQELRDTVSLDLSETASGEGSMGGRKRRPPEQDLNQMGGGGGGGGGGYLLQS
STGAVPASHHHSQIPANFWMVANSNNQVMSGDPIWTFPASVNNSGLYRGTMPGGLHFMN
FPAPMTLLPSQQQLGGSGGGGGGNDGDNMSDGQLNMLAGLNPYRHMSSTGVSESQQAS
GSHSHHGGDDRHDSTSHHS*
> PmTCP19
MEINQTLPTNTLAITDPPENPSLQTQPPQQEEQQQLERRRYIDKHSTVNGRHRRVRIPVTCC
PGIFRLTQELGHRSDGDTIQWLLSQVRPELVLPPQPNNRTRRLPPDPVPQPNCRYPGAAED
WLDHKAVARLPSVTVRATVVQASTVFFDTPATLDKAERLVAGAAAYGSQLVVFPEAFVGG
YPRGLMFDSATATLSPEEKQAFEKYYASAIDVPGPEVDRLAKIASKYKVHLVMGVVERVG
FYLCSTVLFFDSFGQCLGKHPKLLPLASESPVWCSGPKLPVSVYDTEIGRIGGLVCWDNR
MPDLRTQLYGKGIEIYCAPTAEAREIWRSSMTHIALEGGCFVLSANQFCRRKDYPLPLECV
SGDSNDATSLDIICAGGSVIVSPSGTILAGPNYQGESLISADLDLVEIARAKLEFGGVGLGH
NAGPNAVGWRRTSIPNPDLFAATVKTEVSDHANVLYA*

>AtTCP01
MSSSTNDYNDGNNNGVYPLSLYLSSLSGHQDIIHNPYNHQLKASPGHMVSAVPESLIDYM
AFKSNNVVNQQGFEFPEVSKEIKKVVKKDRHSKIQTAQGIRDRRVRLSIGIARQFFDLQDM
LGFDKASKTLDWLLKKSRKAIKEVVQAKNLNNDDEDFGNIGGDVEQEEEKEEDDNGDK
SFVYGLSPGYGEEEVVCEATKAGIRKKKSELRNISSKGLGAKARGKAKERTKEMMAYDN
PETASDITQSEIMDPFKRSIVFNEGEDMTHLFYKEPIEEFDNQESILTNMTLPTKMGQSYNQ
NNGILMLVDQSSSSNYNTFLPQNLDYSYDQNPFHDQTLYVVTDKNFPKGKVWIQDSFVN*
>AtTCP02
MIGDLMKNNNNGDVVDNEVNNRLSRWHHNSSRIIRVSRASGGKDRHSKVLTSKGPRDRR
VRLSVSTALQFYDLQDRLGYDQPSKAVEWLIKAAEDSISELPSLNNTHFPTDDENHQNQT
LTTVAANSLSKSACSSNSDTSKNSSGLSLSRSELRDKARERARERTAKETKERDHNHTSFT
DLLNSGSDPVNSNRQWMASAPSSSPMEYFSSGLILGSGQQTHFPISTNSHPFSSISDHHHHH
PHHQHQEFSFVPDHLISPAESNGGAFNLDFNMSTPSGAGAAVSAASGGGFSGFNRGTLQS
NSTNQHQSFLANLQRFPTSESGGGPQFLFGALPAENHHHNHQFQLYYENGCRNSSEHKGK
GKN*
>AtTCP03
MAPDNDHFLDSPSPPLLEMRHHQSATENGGGCGEIVEVQGGHIVRSTGRKDRHSKVCTA
KGPRDRRVRLSAPTAIQFYDVQDRLGFDRPSKAVDWLITKAKSAIDDLAQLPPWNPADTL
RQHAAAAANAKPRKTKTLISPPPPQPEETEHHRIGEEEDNESSFLPASMDSDSIADTIKSFFP
VASTQQSYHHQPPSRGNTQNQDLLRLSLQSFQNGPPFPNQTEPALFSGQSNNQLAFDSSTA
SWEQSHQSPEFGKIQRLVSWNNVGAAESAGSTGGFVFASPSSLHPVYSQSQLLSQRGPLQS
INTPMIRAWFDPHHHHHHHQQSMTTDDLHHHHPYHIPPGIHQSAIPGIAFASSGEFSGFRIP
ARFQGEQEEHGGDNKPSSASSDSRH*
>AtTCP04
MSDDQFHHPPPPSSMRHRSTSDAADGGCGEIVEVQGGHIVRSTGRKDRHSKVCTAKGPR
DRRVRLSAHTAIQFYDVQDRLGFDRPSKAVDWLIKKAKTSIDELAELPPWNPADAIRLAA
ANAKPRRTTAKTQISPSPPPPQQQQQQQQLQFGVGFNGGGAEHPSNNESSFLPPSMDSDSI
ADTIKSFFPVIGSSTEAPSNHNLMHNYHHQHPPDLLSRTNSQNQDLRLSLQSFPDGPPSLLH
HQHHHHTSASASEPTLFYGQSNPLGFDTSSWEQQSSEFGRIQRLVAWNSGGGGGATDTGN
GGGFLFAPPTPSTTSFQPVLGQSQQLYSQRGPLQSSYSPMIRAWFDPHHHHQSISTDDLNH
HHHLPPPVHQSAIPGIGFASGEFSSGFRIPARFQGQEEEQHDGLTHKPSSASSISRH*
>AtTCP05
MRSGECDEEEIQAKQERDQNQNHQVNLNHMLQQQQPSSVSSSRQWTSAFRNPRIVRVSR
TFGGKDRHSKVCTVRGLRDRRIRLSVPTAIQLYDLQDRLGLSQPSKVIDWLLEAAKDDVD
KLPPLQFPHGFNQMYPNLIFGNSGFGESPSSTTSTTFPGTNLGFLENWDLGGSSRTRARLTD
TTTTQRESFDLDKGKWIKNDENSNQDHQGFNTNHQQQFPLTNPYNNTSAYYNLGHLQQS
LDQSGNNVTVAISNVAANNNNNLNLHPPSSSAGDGSQLFFGPTPPAMSSLFPTYPSFLGAS
HHHHVVDGAGHLQLFSSNSNTASQQHMMPGNTSLIRPFHHLMSSNHDTDHHSSDNESDS
*
>AtTCP06
MVMEPKKNQNLPSFLNPSRQNQDNDKKRKQTEVKGFDIVVGEKRKKKENEEEDQEIQIL
YEKEKKKPNKDRHLKVEGRGRRVRLPPLCAARIYQLTKELGHKSDGETLEWLLQHAEPSI
LSATVNGIKPTESVVSQPPLTADLMICHSVEEASRTQMEANGLWRNETGQTIGGFDLNYGI
GFDFNGVPEIGFGDNQTPGLELRLSQVGVLNPQVFQQMGKEQFRVLHHHSHEDQQQSAE

ENGS*
>AtTCP07
MSINNNNNNNNNNNNDGLMISSNGALIEQQPSVVVKKPPAKDRHSKVDGRGRRIRMPIICA
ARVFQLTRELGHKSDGQTIEWLLRQAEPSIIAATGTGTTPASFSTASVSIRGATNSTSLDHKP
TSLLGGTSPFILGKRVRADEDSNNSHNHSSVGKDETFTTTPAGFWAVPARPDFGQVWSFAG
APQEMFLQQQHHHQQPLFVHQQQQQQAAMGEASAARVGNYLPGHLNLLASLSGGSPGS
DRREEDPR*
>AtTCP08
MDLSDIRNNNNDTAAVATGGGARQLVDASLSIVPRSTPPEDSTLATTSSTATATTTKRSTKD
RHTKVDGRGRRIRMPALCAARVFQLTRELGHKSDGETIEWLLQQAEPAIVAATGTGTIPAN
FSTLSVSLRSSGSTLSAPPSKSVPLYGALGLTHHQYDEQGGGGVFAAHTSPLLGFHHQLQH
HQNQNQNQDPVETIPEGENFSRKRYRSVDLSKENDDRKQNENKSLKESETSGPTAAPMW
AVAPPSRSGAGNTFWMLPVPTTAGNQMESSSNNNTAAGHRAPPMWPFVNSAGGGAGGG
GGAATHFMAGTGFSFPMDQYRGSPLQLGSFLAQPQPTQNLGLSMPDSNLGMLAALNSAY
SRGGNANANAEQANNAVEHQEKQQQSDHDDDSREENSNSSE*
>AtTCP09
MATIQKLEEVAGKDQTLRAVDLTIINGVRNVETSRPFQVNPTVSLEPKAEPVMPSFSMSLA
PPSSTGPPLKRASTKDRHTKVEGRGRRIRMPATCAARIFQLTRELGHKSDGETIRWLLENA
EPAIIAATGTGTVPAIAMSVNGTLKIPTTTNADSDMGENLMKKKRKRPSNSEYIDISDAVSA
SSGLAPIATTTTIQPPQALASSTVAQQLLPQGMYPMWAIPSNAMIPTVGAFFLIPQIAGPSNQ
PQLLAFPAAAASPSSYVAAVQQASTMARPPPLQVVPSSGFVSVSDVSGSNLSRATSVMAPS
SSSGVTTGSSSSIATTTTHTLRDFSLEIYEKQELHQFMSTTTARSSNH*
>AtTCP10
MGLKGYSVGEGGGEIVEVQGGHIIRATGRKDRHSKVFTSKGPRDRRVRLSAHTAIQFYDV
QDRLGYDRPSKAVDWLIKKAKTAIDKLELGETTTTTTRQEPVNTKPESPTLVFQRENNDQ
TQFVAANLDPEDAMKTFFPATTTTNGGGGTNINFQNYPHQDDNNMVSRTTTPPPNLSQDL
GLSLHPFQGNNNTVVVPETNNFTTTHFDTFGRISGWNHHDLTMTSSSSSEHQQQEQEERS
NGGFMVNHHPHHHHHQPSMMTLLNSQQQQVFLGGQQQQQRGTLQSSLFPHSFRSWD
HHQTTSDHHHHQNQASSMFASSSQYGSHGMMMMQGLSFPNTTRLLHGEEATQPNSSSSP
PNSH
L*
>AtTCP11
MIFQNVCRNESNFNAIASESRSQTQFGVSKSSSSGGGCISARTKDRHTKVNGRSRRVTMP
ALAAARIFQLTRELGHKTEGETIEWLLSQAEPSIIAATGYGTKLISNWVDVAADDSSSSSSM
TSPQTQTQTPQSPSCRLDLCQPIGIQYPVNGYSHMPFTAMLLEPMTTTAESEVEIAEEEERR
RRHH*
>AtTCP12
MFPSLDTNGYDLFDPFIPHQTTMFPSFITHIQSPNSHHHYSSPSFPFSSDFLESFDESFLINQF
LLQQQDVAANVVESPWKFCKKLELKKKNEKCVDGSTSQEVQWRRTVKKRDRHSKICTA
QGPRDRRMRLSLQIARKFFDLQDMLGFDKASKTIEWLFSKSKTSIKQLKERVAASEGGGK
DEHLQVDEKEKDETLKLRVSKRRTKTMESSFKTKESRERARKRARERTMAKMKMRLFET
SETISDPHQETREIKITNGVQLLEKENKEQEWSNTNDVHMVEYQMDSVSIIEKFLGLTSDS
SSSSIFGDSEECYTSLSSVRGMSTPREHNTTSIATVDEEKSPISSFSLYDYLCY*
>AtTCP13

MNIVSWKDANDEVAGGATTRREREVKEDQEETEVRATSGKTVIKKQPTSISSSSSSWMKS
KDPRIVRVSRAFGGKDRHSKVCTLRGLRDRRVRLSVPTAIQLYDLQERLGVDQPSKAVDW
LLDAAKEEIDELPPLPISPENFSIFNHHQSFLNLGQRPGQDPTQLGFKINGCVQKSTTTSREE
NDREKGENDVVYTNNHHVGSYGTYHNLEHHHHHHQHLSLQADYHSHQLHSLVPFPSQIL
VCPMTTSPTTTTIQSLFPSSSSAGSGTMETLDPRQMVSHFQMPLMGNSSSSSSQNISTLYSL
LHGSSSNNGGRDIDNRMSSVQFNRTNSTTTANMSRHLGSERCTSRGSDHHM*
>AtTCP14
MQKPTSSILNVIMDGGDSVGGGGGDDHHRHLHHHHRPTFPFQLLGKHDPDDNHQQQPSP
SSSSSLFSLHQHQQLSQSQPQSQSQKSQPQTTQKELLQTQEESAVVAAKKPPLKRASTKDR
HTKVDGRGRRIRMPALCAARVFQLTRELGHKSDGETIEWLLQQAEPSVIAATGTGTIPANF
TSLNISLRSSGSSMSLPSHFRSAASTFSPNNIFSPAMLQQQQQQRGGGVGFHHPHLQGRA
PTSSLFPGIDNFTPTTSFLNFHNPTKQEGDQDSEELNSEKKRRIQTTSDLHQQQQQHQHDQI
GGYTLQSSNSGSTATAAAAQQIPGNFWMVAAAAAAGGGGGNNNQTGGLMTASIGTGGG
GGEPVWTFPSINTAAAALYRSGVSGVPSGAVSSGLHFMNFAAPMAFLTGQQQLATTSNHEI
NEDSNNNEGGRSDGGGDHHNTQRHHHHQQHHHNILSGLNQYGRQVSGDSQASGSLGG
GDEEDQQD*
>AtTCP15
MDPDPDHNHRPNFPLQLLDSSTSSSSTSLAIISTTSEPNSEPKKPPPKRTSTKDRHTKVEGR
GRRIRMPAMCAARVFQLTRELGHKSDGETIEWLLQQAEPAVIAATGTGTIPANFTSLNISLR
SSRSSLSAAHLRTTPSSYYFHSPHQSMTHHLQHQHQVRPKNESHSSSSSSSQLLDHNQMG
NYLVQSTAGSLPTSQSPATAPFWSSGDNTQNLWAFNINPHHSGVVAGDVYNPNSGGSGGG
SGVHLMNFAAPIALFSGQPLASGYGGGGGGGGEHSHYGVLAALNAAYRPVAETGNHNN
NQQNRDGDHHHNHQEDGSTSHHS*
>AtTCP16
MDSKNGINNSQKARRTPKDRHLKIGGRDRRIRIPPSVAPQLFRLTKELGFKTDGETVSWLL
QNAEPAIFAATGHGVTTTSNEDIQPNRNFPSYTFNGDNISNNVFPCTVVNTGHRQMVFPVS
TMTDHAPSTNYSTISDNYNSTFNGNATASDTTSAATTTATTTV*
>AtTCP17
MGIKKEDQKSSLSLLTQRWNNPRIVRVSRAFGGKDRHSKVCTVRGLRDRRIRLSVMTAIQ
VYDLQERLGLSQPSKVIDWLLEVAKNDVDLLPPLQFPPGFHQLNPNLTGLGESFPGVFDL
GRTQREALDLEKRKWVNLDHVFDHIDHHNHFSNSIQSNKLYFPTITSSSSSYHYNLGHLQ
QSLLDQSGNVTVAFSNNYNNNNLNPPAAETMSSLFPTRYPSFLGGGQLQLFSSTSSQPDH
IE*
>AtTCP18
MNNNIFSTTTTINDDYMLFPYNDHYSSQPLLPFSPSSSINDILIHSTSNTSNNHLDHHHQFQ
QPSPFSHFEFAPDCALLTSFHPENNGHDDNQTIPNDNHHPSLHFPLNNTIVEQPTEPSETINL
IEDSQRISTSQDPKMKKAKKPSRTDRHSKIKTAKGTRDRRMRLSLDVAKELFGLQDMLGF
DKASKTVEWLLTQAKPEIIKIATTLSHHGCFSSGDESHIRPVLGSMDTSSDLCELASMWTV
DDRGSNTNTTETRGNKVDGRSMRGKRKRPEPRTPILKKLSKEERAKARERAKGRTMEKM
MMKMKGRSQLVKVVEEDAHDHGEIIKNNNRSQVNRSSFEMTHCEDKIEELCKNDRFAVC
NEFIMNKKDHISNESYDLVNYKPNSSFPVINHHRSQGAANSIEQHQFTDLHYSFGAKPRDL
MHNYQNMY*
>AtTCP19
MESNHEGNAIQVIDQVTTMTHLSDPNPKTKPGMMLMKQEDGYLQPVKTKPAPKRPTSKD

RHTKVEGRGRRIRMPAGCAARVFQLTRELGHKSDGETIRWLLERAEPAIIEATGTGTVPAI
AVSVNGTLKIPTSSPVLNDGGRDGDGDLIKKRRKRNCTSDFVDVNDSCHSSVTSGLAPIT
ASNYGVNILNVNTQGFVPFWPMGMGTAFVTGGPDQMGQMWAIPTVATAPFLNVGARPV
SSYVSNASDAEAEMETSGGGTTQPLRDFSLEIYDKRELQFLGGSGNSSPSSCHET*
>AtTCP20
MDPKNLNRHQVPNFLNPPPPPRNQGLVDDDAASAVVSDENRKPTTEIKDFQIVVSASDKE
PNKKSQNQNQLGPKRSSNKDRHTKVEGRGRRIRMPALCAARIFQLTRELGHKSDGETIQW
LLQQAEPSIIAATGSGTIPASALASSAATSNHHQGGSLTAGLMISHDLDGGSSSSGRPLNWG
IGGGEGVSRSSLPTGLWPNVAGFGSGVPTTGLMSEGAGYRIGFPGFDFPGVGHMSFASILG
GNHNQMPGLELGLSQEGNVGVLNPQSFTQIYQQMGQAQAQAQGRVLHHMHHNHEEHQ
QESGEKDDSQGSGR*
>AtTCP21
MADNDGAVSNGIIVEQTSNKGPLNAVKKPPSKDRHSKVDGRGRRIRMPIICAARVFQLTR
ELGHKSDGQTIEWLLRQAEPSIIAATGTGTTPASFSTASLSTSSPFTLGKRVVRAEEGESGG
GGGGGLTVGHTMGTSLMGGGGSGGFWAVPARPDFGQVWSFATGAPPEMVFAQQQQPAT
LFVRHQQQQQASAAAAAAMGEASAARVGNYLPGHHLNLLASLSGGANGSGRREDDHEP
R*
>AtTCP22
MNQNSSVAEATLQLNSGEKPSPGSIPFISSGQHGNISTSATSSTSTSSGSALAVVKSAVKKPT
KDRHTKVDGRGRRIRMPAMCAARVFQLTRELGHKSDGETIEWLLQQAEPAIIASTGTGTIP
ANFSTLNASLRSGGGSTLFSQASKSSSSPLSFHSTGMSLYEDNNGTNGSSVDPSRKLLNSA
ANAAVFGFHHQMYPPIMSTERNPNTLVKPYREDYFKEPSSAAEPSESSQKASQFQEQELA
QGRGTANVVPQPMWAVAPGTTNGGSAFWMLPMSGSGGREQMQQQPGHQMWAFNPGN
YPVGTGRVVTAPMGSMMLGGQQLGLGVAEGNMAAAMRGSRGDGLAMTLDQHQHQLQ
HQEPNQSQASENGGDDKK*
>AtTCP23
MESHNNNQSNNNTTGSAHLVPSMGPISGSVSLTTTAPNSTTTTVTAAKTPAKRPSKDRHI
KVDGRGRRIRMPAICAARVFQLTRELQHKSDGETIEWLLQQAEPAIIAATGTGTIPANISTLN
ISLRSSGSTLSAPLSKSFHMGRAAQNAAVFGFQQQLYHPHHITTDSSSSSLPKTFREEDLFK
DPNFLDQEPGSRSPKPGSEAPDQDPGSTRSRTQNMIPPMWALAPTPASTNGGSAFWMLPV
GGGGGPANVQDPSQHMWAFNPGHYPGRIGSVQLGSMLVGGQQLGLGVAENNNLGLFSG
GGGDGGRVGLGMSLEQKPQHQVSDHATRDQNPTIDGSP*
>AtTCP24
MEVDEDIELQKHQEQQSRKLQRFSEDNTGLMRNWNNPSSRIIRVSRASGGKDRHSKVLTS
KGLRDRRIRLSVATAIQFYDLQDRLGFDQPSKAVEWLINAASDSITDLPLLNTNFDHLDQN
QNQTKSACSSGTSESSLLSLSRTEIRGKARERARERTAKDRDKDLQNAHSSFTQLLTGGFD
QQPSNRNWTGGSDCFNPVQLQIPNSSSQEPMNHPFSFVPDYNFGISSSSSAINGGYSSRGTL
QSNSQSLFLNNNNNITQRSSISSSSSSSSPMDSQSISFFMATPPPLDHHNHQLPETFDGRLYL
YYGEGNRSSDDKAKERR*
>ZmTB1
MFPFCDSSSPMDLPLYQQLQLSPSSPKTDQSSSFYCYPCSPPFAAADASFPLSYQIGSAAAA
DATPPQAVINSPDLPVQALMDHAPAPATATELGACASGAEGSGASLDRAAAAARKDRHSK
ICTAGGMRDRRMRLSLDVARKFFALQDMLGFDKASKTVQWLLNTSKSAIQEIMADDASS
ECVEDGSSSLSVDGKHNPAEQLGGGGDQKPKGNCRGEGKKPAKASKAAATPKPPRKSAN

NAHQVPDKETRAKARERARERTKEKHRMRWVKLASAIDVEAAAASGPSDRPSSNNLSH
HSSLSMNMPCAAAELEERERCSSALSNRSAGRMQEITGASDVVLGFGNGGYGDGGGNY
YCQEQWELGGVVFQQNSRFY
>AmCYC
MFGKNTYLHLPQVSSSLHSRAATSVVDLNGNEIQLHDMLSGHYLTTANAPVLESTALFNN
NNNFNHDVVNGLNRDPSPTFPTKQAVKKDRHSKIYTSQGPRDRRVRLSIGIARKFFDLQE
MLGFDKPSKTLDWLLTKSKTAIKELVQSKSTKSNSSSPCDDCEEVVSVDSENVTDHSKGK
SLKANNKCKEAMDSHQAAAKESRAKARARARERTKEKMCIKQLNEAIVLRNHQFEVSG
TREAFVHPVFGFHQQNYGNASHENWDQSNLSSQSNQLCAILNQHKFIN
>OsPCF1
MMASSDLILYNLVPAQPLNPSAIPNPNPDLSIAAAEPPSSDGATPRRVRPRKSPSSSDRHSKV
AGRGRRVRIPAMVAARVFQLTRELGHRTDGETIEWLLRQAEPSIIAATGTGVTPEEAPPAAV
AIGSSSVAAAAAAGGHGGAFVHVPYYTALLMQPPNADEPPMASAASASGTTAADENNN
>OsPCF2
MEAQAQDKAEEGEEEGTRQQHAQAGPVGAAGGGGGGGAAAVAMSAIPMNSWLVPKPE
PVEFFGGMAMVRKPPPRNRDRHTKVEGRGRRIRMPAACAARIFQLTRELGHKSDGETIRW
LLQQSEPAIIAATGTGTVPAIATTVDGVLRIPTQSSSSSGPASSAVVDGEESSAKRRRKLQPT
RAVAGASPLATAAPAAYYPVIADPLLQGSGGAAISVPSGLAPITATGAPQGLVPVFAVPATGS
PAVAGGNRMIPQATAVWMVPRPAGAAGAGNQPTQFWAIQSAPQLVNFAGAQFPTAINVAD
FQQQQQQQPVSTTIVQNSNSGEHMHFSGADSHEQQRRGRKEGNSGGVVDHPEEDEDDD
DDEPVSDSSPEE
>AmCIN
MGGAGGGGESSNNTTHNHTTTTTSSRLGLRNSVGGEIVEVQGGHIVRSTGRKDRHSKVC
TAKGPRDRRVRLAAHTAIQFYDVQDRLGYDRPSKAVDWLIKKAKSAIDELAQLPAWDPTA
CSVAATNSSFKHEEEQQQMGIDMQQQQQQNDMMMSNEQQQQQGLHNNAVVSNQDSNF
LPPSLDSDAIADTIKSFFPMGASNEASSTAMQFQSFTTSDLLSRTTSHSHSQDLRLSLQSFQ
DPIMLHHHHNQSQQHQNHHSEQPHSQSHHVLLSGTPLGFDSGWQEHQHQQAAELSRFQR
FAAWNPSGESGGGGASASSYLFNSPPPQAPQPLLQQLLSHNNQFFSQRGPLQSSNTPSVRA
WMDPASAMADHSHHQSNVPIYPSSISGIGFSSGVGGFPGFHIPARIQGDEEEHDGYSDKPSS
ASSESRH
